# Investigation of the role of typhoid toxin in acute typhoid fever in a human challenge model

**DOI:** 10.1038/s41591-019-0505-4

**Published:** 2019-07-03

**Authors:** Malick M. Gibani, Elizabeth Jones, Amber Barton, Celina Jin, Juliette Meek, Susana Camara, Ushma Galal, Eva Heinz, Yael Rosenberg-Hasson, Gerlinde Obermoser, Claire Jones, Danielle Campbell, Charlotte Black, Helena Thomaides-Brears, Christopher Darlow, Christina Dold, Laura Silva-Reyes, Luke Blackwell, Maria Lara-Tejero, Xuyao Jiao, Gabrielle Stack, Christoph J. Blohmke, Jennifer Hill, Brian Angus, Gordon Dougan, Jorge Galán, Andrew J. Pollard

**Affiliations:** 1 Oxford Vaccine Group, Department of Paediatrics, University of Oxford and the NIHR Oxford Biomedical Research Centre, Oxford, UK; 2 Department of Medicine, Imperial College London, London, UK; 3 Nuffield Department of Primary Care Health Sciences, Clinical Trials Unit, University of Oxford, Oxford, UK; 4 Wellcome Sanger Institute, Wellcome Genome Campus, Hinxton, UK; 5 Department of Vector Biology, Liverpool School of Tropical Medicine, Liverpool, UK; 6 Human Immune Monitoring Center, Institute for Immunity, Transplantation and Infection, Stanford University, Stanford, CA USA; 7 Department of Microbial Pathogenesis, Yale University School of Medicine, New Haven, CT USA; 8 Nuffield Department of Medicine, University of Oxford, Oxford, UK; 9 Department of Medicine, University of Cambridge, Hinxton, UK

**Keywords:** Bacteriology, Experimental models of disease, Translational research, Clinical trial design, Clinical trials

## Abstract

*Salmonella* Typhi is a human host-restricted pathogen that is responsible for typhoid fever in approximately 10.9 million people annually^1^. The typhoid toxin is postulated to have a central role in disease pathogenesis, the establishment of chronic infection and human host restriction^2–6^. However, its precise role in typhoid disease in humans is not fully defined. We studied the role of typhoid toxin in acute infection using a randomized, double-blind *S.* Typhi human challenge model^7^. Forty healthy volunteers were randomized (1:1) to oral challenge with 10^4^ colony-forming units of wild-type or an isogenic typhoid toxin deletion mutant (TN) of *S.* Typhi. We observed no significant difference in the rate of typhoid infection (fever ≥38 °C for ≥12 h and/or *S*. Typhi bacteremia) between participants challenged with wild-type or TN *S*. Typhi (15 out of 21 (71%) versus 15 out of 19 (79%); *P* = 0.58). The duration of bacteremia was significantly longer in participants challenged with the TN strain compared with wild-type (47.6 hours (28.9–97.0) versus 30.3(3.6–49.4); *P* ≤ 0.001). The clinical syndrome was otherwise indistinguishable between wild-type and TN groups. These data suggest that the typhoid toxin is not required for infection and the development of early typhoid fever symptoms within the context of a human challenge model. Further clinical data are required to assess the role of typhoid toxin in severe disease or the establishment of bacterial carriage.

## Abstract (web summary)

Typhoid toxin is not essential for the pathogenesis of typhoid fever in healthy humans challenged with *Salmonella* Typhi.

## Main

Several preclinical studies have described the structure and function of the typhoid toxin in vitro and in small-animal models^2–6^. Systemic administration of typhoid toxin to C57BL/6 mice results in the reproduction of many characteristic symptoms of typhoid fever; other studies have suggested that typhoid toxin may contribute to the establishment of chronic infection^4,5^. It remains unclear how the surrogate end points of illness in mice—such as lethargy, weight loss, behavioral and motor changes^3,8^—are representative of acute typhoid fever in humans. The toxin is also encoded by >40 clade B non-typhoidal *Salmonella* (NTS) serovars that display a broad host range and a distinct clinical phenotype to *S*. Typhi and Paratyphi^9–11^, although some typhoid toxin-expressing NTS serovars appear to cause an enteric fever-like syndrome^12^. Importantly, no previous studies have characterized the role of typhoid toxin in a human model of disease.

**Fig. 1:**
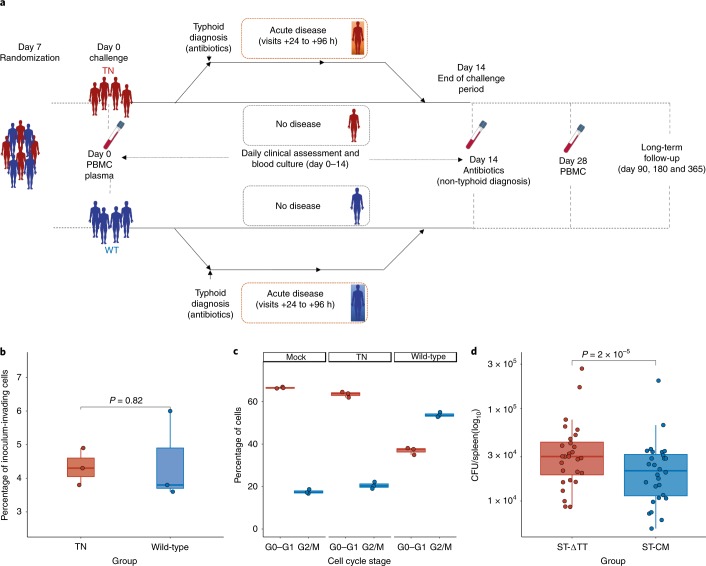
Trial design. **a**, Schematic of trial design (Supplementary Information); comparison of wild-type and TN strains. **b**, Cellular invasion assessed using a gentamicin protection assay. MOI = 50, *n* = 3 independent replicates, two-sided Mann–Whitney U-test. **c**, Induction of cell cycle arrest in Henle-407 cells infected with the wild-type or TN strain (MOI = 50). Intoxicated cells show a larger proportion of cells in the G2/M phase of the cell cycle. *n* = 3 independent replicates. **d**, Comparison of the bacterial loads of *S*. Typhi Quailes typhoid toxin-null mutant (ST-ΔTT, ∆pltB, ∆pltA, ∆cdtB) with *S*. Typhi Quailes typhoid toxin catalytic mutant (ST-CM, *cdtB*^H160Q^, *pltB*^S35A^, *pltA*^E133A^). *n* = 28, Wilcoxon signed-rank test. The box plots display the median and IQR, with the upper whiskers extending to the largest value ≤1.5 × IQR from 75th percentile and the lower whiskers extending to smallest values ≤1.5 × IQR from 25th percentile. The Fig. 1a images were sourced from Servier Medical Art and reproduced and adapted under a Creative Commons 3.0 unported license^29^. Source data

We aimed to characterize the role of typhoid toxin in human infection and pathogenesis using an *S*. Typhi human challenge model^7^. This model has previously been used to test novel live-attenuated (MO1ZH09) (ref. ^13^) and Vi-conjugate (Typbar-TCV) typhoid vaccines^14^. We manufactured two challenge strains of *S*. Typhi to good manufacturing practice (GMP) standards. We used the wild-type *S*. Typhi Quailes strain (genotype 3.0.1 (ref. ^15^)) as the parent strain to generate an isogenic typhoid toxin-deficient knockout strain (TN), as described previously^16^. Whole-genome sequencing confirmed the absence of the typhoid toxin pathogenicity islet in the TN strain (Supplementary Information). The wild-type and TN strains harbored no other differences in relation to known key virulence factors (Supplementary Information). In particular, there were no differences identified in *Salmonella* pathogenicity island 7, a region encoding genes required for expression of the Vi-capsule—a key virulence factor in the pathogenesis of *S*. Typhi. Differences between strains were confined to highly variable regions encoding phage proteins, which were not known to impact on bacterial survival in the environment or persistence in the human host. However, deletion of the entire typhoid toxin pathogenicity island was associated with increased bacterial burden in a mouse model of *S*. Typhi infection, compared with a strain expressing a catalytic mutant of typhoid toxin (*cdtB*^H160Q^ *pltB*^S35A^ *pltA*^E133A^; Fig. 1). Otherwise, the wild-type and TN challenge strain variants displayed comparable phenotypic properties with regards to Vi-capsule expression, cellular invasion, in vitro growth characteristics, antibiotic susceptibility and survival in environmental water and soil samples (data not shown). Cell cycle arrest in vitro was observed with the wild-type but not the TN strain (Fig. 1).

**Extended Data Fig. 1:**
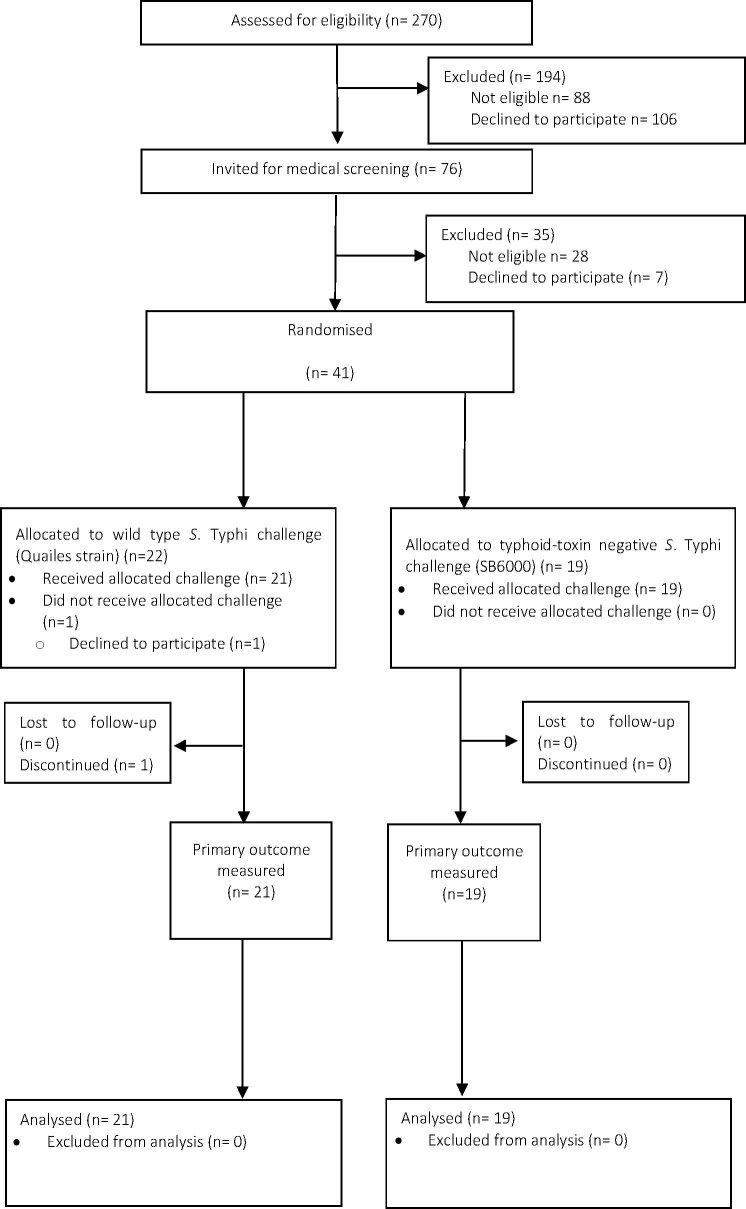
Trial profile. One participant randomized to wild-type *S*. Typhi withdrew before challenge and was excluded from all further analyses, leaving 40 participants in the per-protocol analysis.

**Extended Data Fig. 2:**
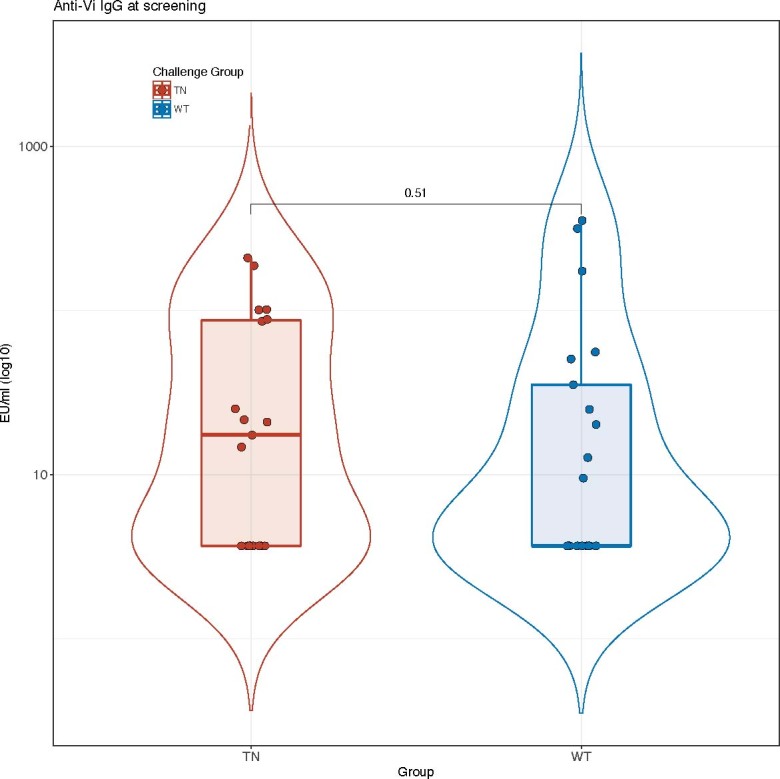
Baseline anti-Vi IgG. *S*. Typhi Quailes strain (*n* = 21). Two-sided Mann–Whitney *U*-test. The box plots represent the median and IQR. The overlaid violin plots illustrate the distribution of the data points and their probability density. Samples below the lower limit of detection of the ELISA (7.4 EU ml^−1^) were assigned a value equating to half the lower limit of detection (3.7 EU ml^−1^)14. The box plots display the median and IQR, with the upper whiskers extending to the largest value ≤1.5 × IQR from the 75th percentile and the lower whiskers extending to the smallest values ≤1.5 × IQR from the 25th percentile. The overlaid violin plots illustrate the distribution of the data points and their probability density^31^. TN *S*. Typhi (*n* = 19). Source data

We enrolled a total of 41 healthy adults (aged 18–60 years) into a randomized, double-blind, human challenge study between 10 April and 1 August 2017. One volunteer withdrew prior to challenge, and 40 completed the challenge protocol (Extended Data Fig. 1). The study was undertaken in a cohort of healthy adult volunteers in a setting non-endemic for typhoid fever (Oxford^14^; see the Life Sciences Reporting Summary). Groups were well matched at baseline (Extended Data Fig. 2 and Supplementary Data). Participants fasted for 90 min before oral challenge with 1–5 × 10^4^ colony-forming units (CFUs) of either wild-type or TN strains administered 2 min after sodium bicarbonate pretreatment (Supplementary Information). Study visits were scheduled for 12 h after challenge, and then daily for 14 d, when daily blood cultures were collected (Fig. 1) (ref. ^7^). Antibiotic treatment (ciprofloxacin 500 mg twice daily) was initiated at typhoid diagnosis or at day 14 for those without illness.

**Fig. 2:**
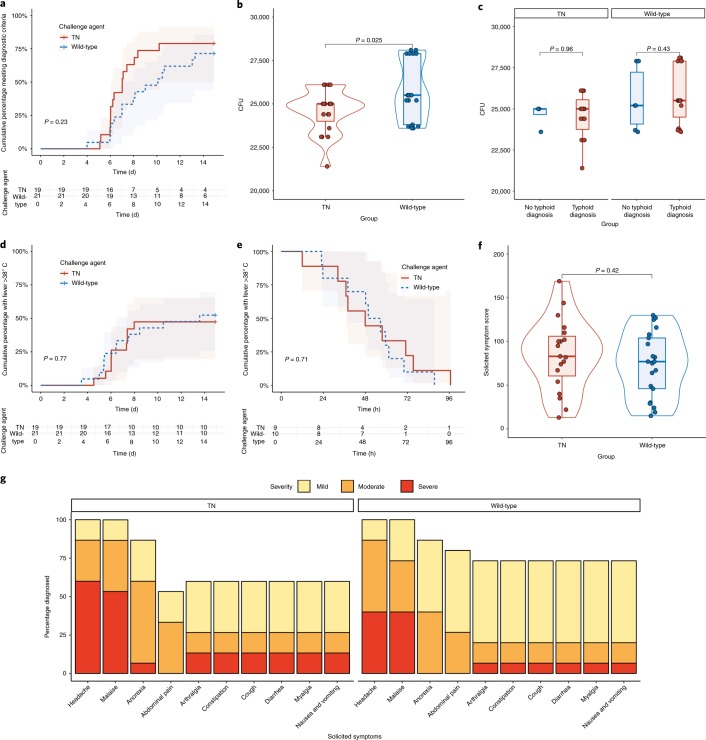
Clinical response to challenge with wild-type and TN *S*. Typhi. **a**, Time to diagnosis after challenge. Cumulative proportion of participants meeting the composite diagnostic end point defined as *S*. Typhi bacteremia and/or fever ≥38 C° persisting ≥12 h. Participants not meeting the diagnostic criteria for typhoid diagnosis were censored at day 14. log-rank test. **b**, Challenge dose administered between wild-type and TN challenge groups. Two-sided Mann–Whitney *U*-test, *n*^TN^ = 19, *n*^Wild-type^ = 21. **c**, Challenge dose administered according to outcome and challenge strain. Mann–Whitney *U*-test. **d**, Time to first fever >38 °C. **e**, Fever clearance time categorized according to study group. Kaplan–Meier survival curve showing the cumulative proportion of participants with any fever >38 °C by challenge group. Participants with no recorded fever were censored at day 14. log-rank test. **f**, Cumulative symptom severity scores in all participants challenged^30^; *n*^TN^ = 19, *n*^Wild-type^ = 21. Two-sided Mann–Whitney *U*-test. **g**, Maximum symptom severity score (day 0–21) in participants diagnosed with typhoid fever according to study group. Percentage of participants reporting one or more events, graded as mild, moderate or severe^30^. The box plots display the median and IQR, with the upper whiskers extending to largest value ≤1.5 × IQR from the 75th percentile and the lower whiskers extending to the smallest values ≤ 1.5 × IQR from the 25th percentile^31^. The overlaid violin plots illustrate the distribution of the data points and their probability density^31^. Source data

Using the primary composite diagnostic end point of fever ≥38 °C for ≥12 h and/or *S*. Typhi bacteremia, we observed no significant difference in the rate of typhoid disease between participants challenged with wild-type or TN strains (15 out of 21 (71%) versus 15 out of 19 (79%); relative risk 1.11 (95% confidence interval (CI) 0.8–1.6); *P* = 0.58; Fig. 2 and Supplementary Data). The attack rate (*n*^diagnosed^/*n*^challenged^) in the wild-type group met the target range of 60–75% and was consistent with earlier studies^7,13,14^. There was no significant difference in the attack rate when we applied alternative diagnostic criteria (Supplementary Data). The challenge dose administered did not impact the outcome of the challenge (Fig. 2). Furthermore, there was no significant difference in time to diagnosis between wild-type and TN groups (median (interquartile range (IQR)) days to diagnosis 7.05 (5.08–8.83) versus 5.25 (5.01–6.14); *P* = 0.23; Fig. 2).

**Extended Data Fig. 3:**
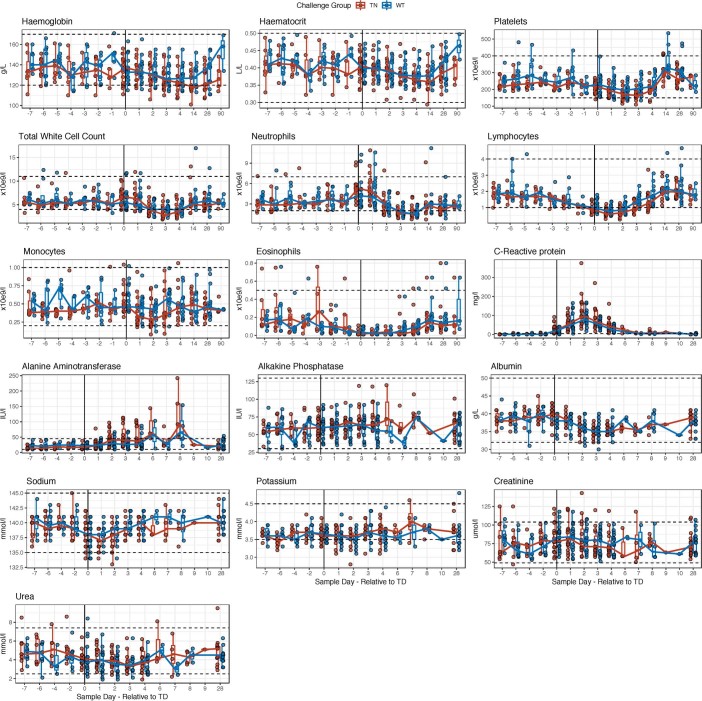
Hematological and biochemical laboratory measures after challenge. Participants challenged with TN (red) and wild-type (blue) *S*. Typhi and diagnosed with typhoid fever, *n*^TN^ = 15, *n*^Wild-type^ = 15. Data are presented relative to the time of typhoid diagnosis (time point 0 = day of diagnosis). The dot plots corresponds to individual values colored according to challenge group (TN, red; wild-type, blue). The solid, colored lines connect the median values at each time point. The dashed lines represent the upper and lower reference limits for the individual parameters measured. The box plots display the median and IQR, with the upper whiskers extending to the largest value ≤1.5 × IQR from the 75th percentile and the lower whiskers extending to the smallest values ≤1.5 × IQR from the 25th percentile^31^, and are colored according to challenge group. Source data

To determine if absence of the typhoid toxin was associated with an altered disease phenotype, we compared the clinical profiles between challenge groups (Fig. 2). Five participants met the prespecified criteria for severe typhoid fever; of these, one participant was randomized to wild-type (1 out of 15; 7%) and four (4 out of 15; 27%; *P* = 0.3) were randomized to TN (Supplementary Data). Two serious adverse events were reported, neither of which was assessed as being related to *S*. Typhi challenge (Supplementary Information). The most common symptoms reported by participants who developed typhoid were headache (30 out of 30; 100%), malaise (30 out of 30; 100%), anorexia (26 out of 30; 87%) and abdominal pain (23 out of 30; 77%; Fig. 2 and Supplementary Information). Fever clearance time was comparable between wild-type and TN groups (median (IQR) hours 53.15 (23.0–87.4) versus 44.92 (12–96.6) hours; *P* = 0.71; Fig. 2). Laboratory abnormalities (elevated C-reactive protein, lymphopenia, neutropenia) were all consistent with the expected presentation of typhoid fever in the field^17^ (Extended Data Fig. 3). Overall, the clinical phenotype was comparable between groups.

**Fig. 3:**
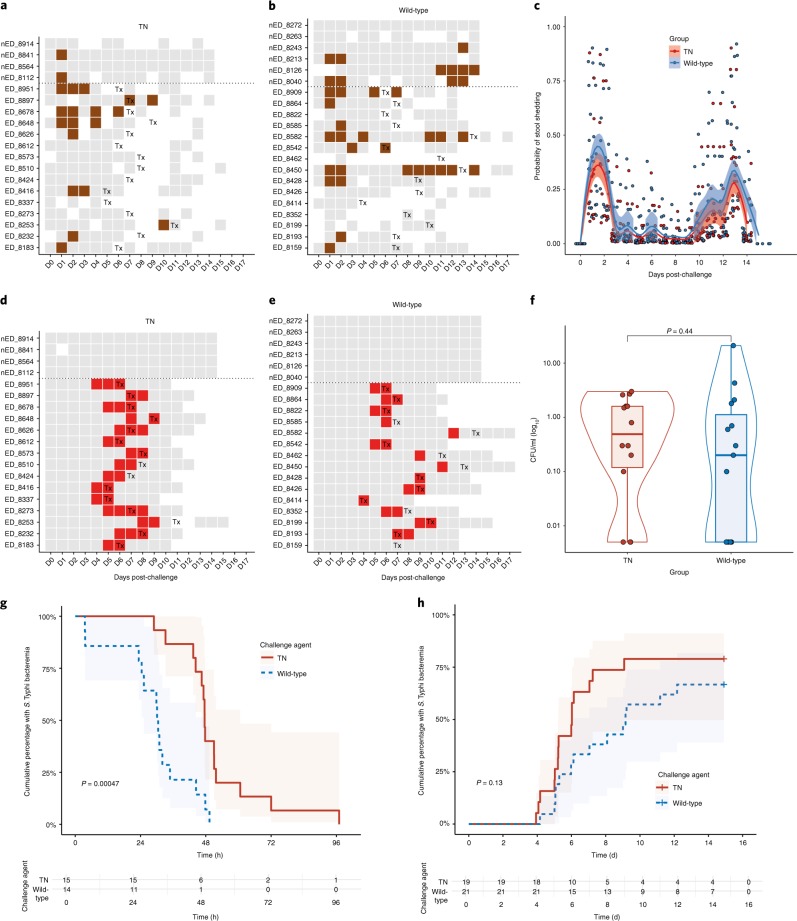
Microbiological response to challenge with wild-type and TN *S*. Typhi. **a**,**b**, Pattern of stool shedding after TN (**a**) and wild-type (**b**) challenge. The rows correspond to individual participants. Gray squares, negative sample; brown squares, positive stool culture; white squares = no sample collected. Tx is the day of treatment initiation. **c**, Probability of stool shedding *S*. Typhi over time after challenge. Samples were classified as culture-positive or culture-negative for *S*. Typhi and combined in mixed effects logistic regression models, as described previously^18^. *n*^Wild-type^ = 21, *n*^TN^ = 19. **d**,**e**, Pattern of bacteremia after TN (**d**) and wild-type (**e**) challenge. Red squares = positive blood culture. Participants above the dotted lines did not meet the composite criteria for typhoid diagnosis. **f**, Quantitative blood culture at time of typhoid diagnosis. Samples with no colonies were assigned an arbitrary value corresponding to half the lower limit of detection (0.05 CFU ml^−1^). *n*^Wild-type^ = 15, *n*^TN^ = 15, two-sided Mann–Whitney *U*-test. **g**,**h**, Kaplan–Meier survival curve showing the cumulative proportion of participants with bacteremia after challenge (**g**) and time to *S*. Typhi bacteremia (**h**). Participants not meeting the diagnostic criteria were censored at day 14. Cumulative proportion of participants with ongoing bacteremia were measured from time of treatment initiation to first persistently negative blood culture, according to challenge group. log-rank test. The box plots display the median and IQR, the upper whiskers extending to the largest value ≤1.5 × IQR from the 75th percentile and the lower whiskers extending to the smallest values ≤1.5 × IQR from the 25th percentile. The overlaid violin plots illustrate the distribution of the data points and their probability density^31^. Source data

**Extended Data Fig. 4:**
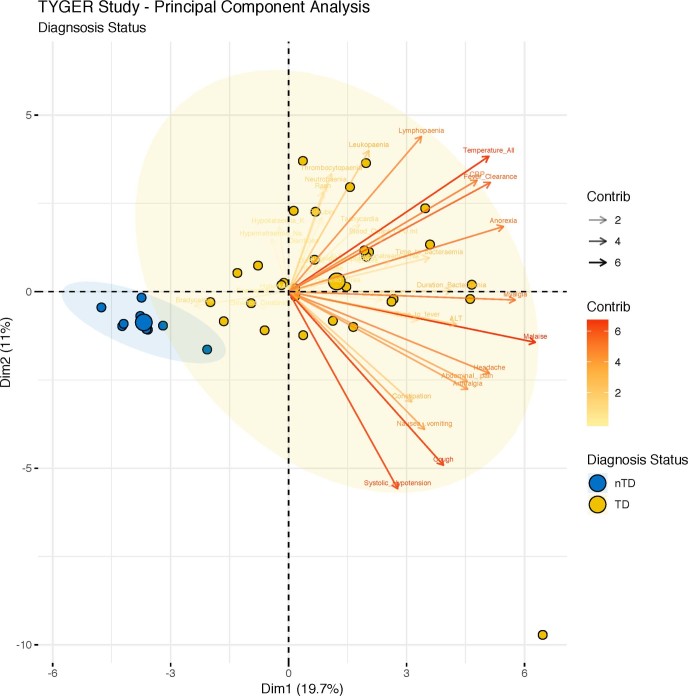
Principal component analysis (PCA) biplot of disease severity by outcome of challenge. Participants challenged with TN (*n* = 19) or wild-type (*n* = 21) *S*. Typhi based on the typhoid severity score. The data points are colored by outcome of challenge (yellow, typhoid diagnosis; blue, no typhoid diagnosis). The ellipses represent the 95% confidence levels for diagnosis status. The biplot arrows represent the contributions of individual variables to a given principal component, scaled according to their relative contribution^36^. Source data

**Extended Data Fig. 5:**
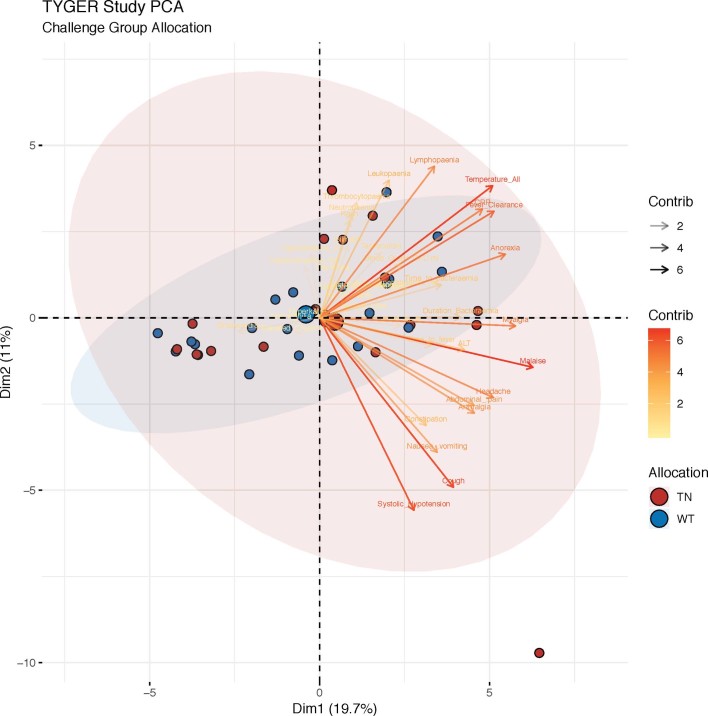
PCA biplot of disease severity by challenge agent. PCA biplot of participants challenged with typhoid fever following challenge with TN (*n* = 19) or wild-type (*n* = 21) *S*. Typhi based on all clinical, microbiological and laboratory measures. The data points are colored by challenge agent allocation. The ellipses represent the 95% confidence levels for challenge agent. The biplot arrows represent the contributions of individual variables to a given principal component, scaled according to their relative contribution^36^. Source data

We next assessed if absence of typhoid toxin was associated with altered microbiological end points (Fig. 3). At least one stool culture was positive for *S*. Typhi in 13 out of 21 (62%) participants challenged with the wild-type strain and 11 out of 19 (58%) challenged with the TN strain. The pattern of stool shedding was comparable between groups, peaking 24–48 h after challenge, followed by a second peak in week 2 (Fig. 3) (ref. ^18^). There was no difference in the probability of shedding over the entire challenge period following challenge with TN compared with wild-type (odds ratio 0.64, 95% CI 0.17–2.47, *P* = 0.51). In a humanized mouse model, infection with a typhoid toxin-deficient strain of *S*. Typhi was associated with an increased bacterial burden compared with the wild-type strain^19^. Consistent with this observation, the duration of bacteremia was significantly longer in participants challenged with the TN strain compared with the wild-type strain (47.6 h (28.9–97.0) versus 30.3 (3.6–49.4); *P* ≤ 0.001; Fig. 3), although circulating quantitative colony counts did not differ (0.2 CFU ml^−1^ (0–21) versus 0.55 CFU ml^−1^ (0–3); *P* = 0.44; Fig. 3). We next performed a principal component analysis of disease severity, using all clinical, microbiological and laboratory measures collected during the course of the challenge study. When all participants were included in the analysis, participants diagnosed with typhoid fever clearly cluster separately from individuals who did not develop disease (Extended Data Fig. 4); however, there was no clustering of participants by challenge group, suggesting that challenge with a typhoid toxin-deficient strain of *S*. Typhi was associated with an indistinguishable clinical phenotype to that caused by wild-type *S*. Typhi (Extended Data Fig. 5).

**Fig. 4:**
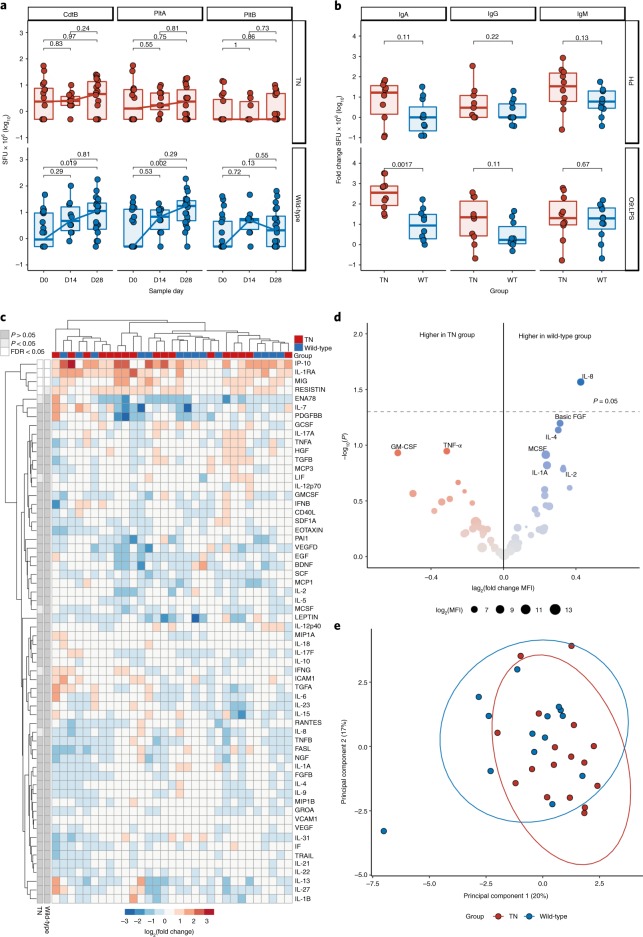
Host response to challenge with wild-type and TN *S*. Typhi. **a**, CdtB-, PltA- and PltB-specific IFN-γ-producing PBMCs at baseline, day 14 and 28 after challenge. *n*^Wild-type^ = 21, *n*^TN^ = 19, Wilcoxon signed-rank test for within-group comparisons on paired samples. **b**, Magnitude of ASC response at typhoid diagnosis. *n*^Wild-type^ = 11, *n*^TN^ = 10; two-sided Mann–Whitney *U*-test. The box plots display the median and IQR. **c**, Plasma cytokine profiles after challenge with wild-type and TN *S*. Typhi. Heatmap showing log_2_ fold change in MFI for each cytokine (rows) and participant (columns) at time of diagnosis relative to baseline. *n*^Wild-type^ = 15, *n*^TN^ = 15. Rows are annotated by significance of cytokine up- or downregulation relative to baseline in each challenge group (white, significant after adjustment for multiple testing; light gray, significant before adjustment; dark gray, non-significant). Two-sided moderated *t*-test with Benjamin–Hochberg correction. Clustering by Euclidean distance. FDR, false discovery rate. **d**, Volcano plots illustrate plasma cytokine up/downregulation at typhoid diagnosis in wild-type and TN challenge groups, with adjustment for baseline. The size of each point reflects average abundance (log_2_MFI) in the plasma. *n*^Wild-type^ = 15, blue; *n*^TN^ = 15, red. **e**, Principal component plot of log_2_ fold change in MFI relative to baseline for each participant. Ellipses are drawn with a 95% confidence level. *n*^Wild-type^ = 15, *n*^TN^ = 15. FGFB, basic fibroblast growth factor; GM-CSF, granulocyte-macrophage colony-stimulating factor; M-CSF, macrophage colony-stimulating factor. Source data

**Extended Data Fig. 6:**
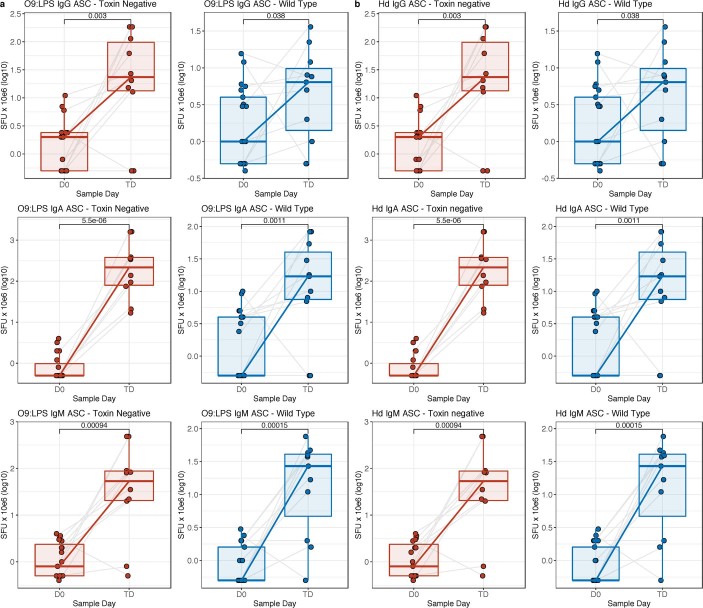
09:LPS- and Hd-specific IgG, IgA and IgM ex vivo ASC responses. **a**, 09:LPS. **b**, Hd. Participants diagnosed with typhoid, illustrated as log_10_ SFUs per 10^6^ PBMCs. D0, baseline; TD, time of diagnosis (samples processed 24–48 h after initiation of treatment). Two-sided matched-pairs Wilcoxon signed-rank test. *n*^Wild-type^ = 11, *n*^TN^ = 10. The box plots display the median and IQR, with the upper whiskers extending to the largest value ≤1.5 × IQR from the 75th percentile and the lower whiskers extending to the smallest values ≤1.5 × IQR from the 25th percentile. Source data

To determine if absence of the typhoid toxin modulated host immune responses to infection, we measured T-cell and antibody-secreting cell (ASC) responses between challenge groups. Interferon-γ (IFN-γ)-producing T-cell responses to peptide pools comprising the typhoid toxin subunits CdtB, PltA and PltB were detectable in participants challenged with the wild-type strain, but not the TN strain, and peaked at day 28 post-challenge (Fig. 4). We observed a significant increase in circulating ASCs specific to the *S*. Typhi surface antigens O9:LPS and Hd at the time of typhoid diagnosis in both challenge groups (Extended Data Fig. 6) (ref. ^13^). The magnitude of the O9:LPS-antigen- and Hd-antigen-specific ASC response at typhoid diagnosis was generally greater in participants challenged with the TN strain. In particular, *S*. Typhi O9:LPS-specific immunoglobulin A ASC responses at the time of typhoid diagnosis were significantly increased in the TN group (Fig. 4).

We next aimed to determine if the presence or absence of typhoid toxin was associated with a distinct plasma cytokine profile, measured using a 62-plex bead-based cytokine platform (Luminex) at baseline and during acute typhoid disease (Fig. 4). At the time of typhoid diagnosis, the plasma cytokines 10 kDa interferon-gamma-induced protein (IP-10), monokine induced by interferon-gamma (MIG) and interleukin-1 receptor antagonist protein (IL-1RA) were significantly increased relative to baseline in both groups (Fig. 4). Hierarchical clustering and principal component analysis showed no separation of challenge groups by cytokine profile during acute typhoid disease (Fig. 4). Following adjustment for multiple testing, linear modeling found no cytokines to be significantly different between groups, although interleukin 8 (IL-8) was marginally downregulated in the toxin-negative but not the wild-type group (Fig. 4).

These data suggest that the typhoid toxin is not essential for *S*. Typhi infection nor the early acute presentation of typhoid fever. This study represents the first application of a typhoid human challenge model to prospectively study the role of a specific virulence factor in the pathogenesis of typhoid fever. Previous trials of live-attenuated *S*. Typhi vaccines have offered insights into the importance of other *Salmonella* genes to human disease (including *aroC/aroD*, *htrA*, *phoP, phoQ*, *ssaV* and *cya*)^20^. Overall, in this study, the clinical presentation was indistinguishable between the TN and wild-type groups. Counterintuitively, there was a trend toward a more severe disease phenotype in the TN group, including a shorter time to diagnosis, higher number of cases meeting the criteria for severe enteric fever, elevated ASC response and prolonged duration of bacteremia. These observations suggest that the typhoid toxin may have an important role in modifying host immune responses to infection.

These data raise questions as to the utility of targeting typhoid toxin in the development of novel therapeutics or vaccine strategies. Notably, currently utilized typhoid vaccines, including Vi capsular polysaccharide/conjugate vaccines, are capable of inducing protection despite targeting a virulence factor that is not strictly necessary for the establishment of enteric fever^21–23^. Antibody and T-cell responses to typhoid toxin components have been detected in patients with typhoid fever^24–27^. Further studies are required to correlate host responses to typhoid toxin with protection against disease and to further characterize its function in the context of natural *S*. Typhi infection.

We acknowledge the limitations of our experimental approach. Due to ethical considerations, this model is not suited to assess the role of typhoid toxin in severe typhoid fever, including typhoid encephalopathy, which has been associated with typhoid toxin in animal models^4,8^. The primary diagnostic criteria minimizes risk to study participants by early treatment initiation^7^, but could mask differences between groups by treating self-limiting disease. The study population may not be generalizable to typhoid endemic countries, owing to differences in prior immune-priming and/or baseline genetic differences^28^.

The absence of experiments showing reversion to virulence after complementation of the typhoid toxin genes in vitro is a limitation of this study. Additional studies with a strain expressing inactive components of typhoid toxin (for example, *cdtB*^H160Q^, *pltB*^S35A^, *pltA*^E133A^) could address whether deletion of typhoid toxin genes is associated with an altered phenotype beyond loss of toxicity. Importantly, the study was underpowered to detect anything other than a large effect size (80% power to detect at 72% relative reduction). Additional in vitro studies and deeper analysis of challenge samples are ongoing to further characterize the potential immunobiological role of typhoid toxin in the pathogenesis of typhoid fever.

These data indicate that typhoid toxin is not essential for the development of early acute typhoid fever within the context of a controlled human infection model. These data highlight some of the benefits and challenges of studying bacterial virulence factors using controlled human infection models, in particular for the screening of potential vaccine and therapeutic targets.

## Methods

### Study design and participants

The OVG2016/03 (TYGER) study was a randomized, double-blind, controlled human infection study comparing the response to challenge with wild-type *S*. Typhi with a typhoid toxin-deficient isogenic mutant strain *S*. Typhi (SB6000). The study was designed as an outpatient challenge study, conducted in a cohort of healthy community adult volunteers in a setting non-endemic for typhoid fever (Oxford).

### Challenge strains

To facilitate comparisons with earlier challenge studies^7,13,14^ and to minimize the risk to study participants, we used the *S*. Typhi Quailes strain (genotype 3.0.1 (ref. ^15^)) as the parent strain to generate the typhoid toxin-deficient knockout strain. Deletion of typhoid toxin subunit genes was carried as described previously^16^.

Briefly, deletion of typhoid toxin subunit genes was carried out using the R6K-derived, suicide vector pSB890. The pSB890 plasmid cannot replicate in *S*. Typhi since it requires the bacteriophage λpir protein to replicate. The plasmid vector also encodes a counterselectable marker *sacB*, which encodes an enzyme that is lethal to bacteria when grown in the presence of sucrose. The pSB890 plasmid vector is maintained in a specially constructed strain of *Escherichia coli*, which encodes the bacteriophage λpir protein. This *E. coli* strain also carries a deletion mutation in the *asd* gene(*∆asd)*, which encodes the aspartate-semialdehyde dehydrogenase required for peptidoglycan synthesis—growth of this strain will only occur in media supplemented with lysine diaminoheptanedioate.

Due to the genomic organization of the typhoid toxin pathogenicity island, the *cdtB* gene was deleted first, followed by simultaneous deletion of the *pltA* and *pltB* genes (encoded immediately adjacent to one another) in the *∆cdtB* strain. Chromosomal DNA fragments encoding sequences upstream and downstream of the target genes were expanded by PCR and cloned into the pSB890 plasmid, maintained in the *E*. *coli* *∆asd* λpir strain. The plasmid vector encoding the cloned sequences was then transferred to *S*. Typhi by conjugation, counterselecting the donor *E. coli* strain by plating the transconjugants in media lacking lysine diaminoheptanedioate (counterselecting for the donor *E*. *coli*). Transconjugants of *S*. Typhi possessing deletions of the toxin integrated into the chromosome were identified by plating in sucrose that counterselects for the plasmid vector. Colonies were screened by PCR to identified mutants carrying the specific deletions.

Challenge strains were manufactured to a GMP standard at the Walter Reed Army Institute of Research (Silver Spring) and stored as a frozen suspension in soya tryptone medium containing 10% sucrose at −80 °C before use.

### Strain characterization

Growth curves of wild-type and toxin-deficient strains of *S*. Typhi were performed in lysogeny broth (LB) using wild-type and TN strains. Isolates were inoculated into 10 ml of LB and grown overnight in a shaking incubator at 200–220 r.p.m. and 37 °C. The following day, cultures were vortexed and diluted 1:10 in fresh LB (100 µl culture + 900 µl LB). The OD600 was read in a cuvette using a mini photospectrometer against an LB-only blank, multiplying by the dilution factor (×10) to give the actual OD of the overnight culture. The dilution required to reduce the culture OD to 0.05 in a 30 ml volume of LB was calculated using the following equation (equation (1)):

$${\mathrm{OD}}1 \times {\mathrm{V}}1 = {\mathrm{OD}}2 \times {\mathrm{V}}2$$

where OD1 is the OD of the overnight culture, V1 is the volume of the overnight culture to be added to the new mix; OD2 is theOD of the new inoculum (0.05) and V2 is the volume of new inocula (30 ml). The calculated volume of overnight cultures (V1) was added to 30 ml of fresh LB, the OD600 read to ensure the OD of the new culture was 0.05 and was subsequently returned to the shaking incubator. Samples of culture were removed from the incubator at regular intervals and the OD600 was measured in a 50:50 mix of the culture and fresh LB against an LB blank. OD600 readings were multiplied by the dilution factor (×2) to give values for the undiluted culture and plotted against time to give the growth curve.

#### Typhoid toxin activity

Activity of the typhoid toxin was assessed using previously published methods^4,6^. Briefly, Henle-407 intestinal epithelial cells were infected with wild-type Quailes or typhoid toxin-deficient SB6000 *S*. Typhi for 1 h. Cells were washed and culture medium containing gentamicin (50 μg ml^−1^) was added and then incubated for 2 h. Cell were then washed and medium containing 5 μg ml^−1^ gentamicin was added and infection continued for 48 h. Cells were collected from dishes by trypsinization (subsequently neutralized with serum-containing media). The cell suspensions were centrifuged for 5 min at 1500 r.p.m., the supernatant discarded and cell pellets resuspended in 0.5 ml of PBS at room temperature. Cell suspensions were slowly added to tubes containing 4 ml of cold 90% ethanol solution with continuous mixing. Cells were kept in fixative for 2 h on ice. The fixed cells were collected by centrifugation and the fixative decanted thoroughly. The pellets were washed once with 5 ml PBS and the cell pellet was resuspended in 1 ml of a solution containing 0.1% Triton X-100, DNase-free ribonuclease A (20 mg ml^−1^) and propidium iodide (20 μg ml^−1^; Molecular Probes) in PBS. The stained cells were analyzed by flow cytometry with a FACStar Plus flow cytometer (BD Biosciences). Intoxicated cells showed a larger proportion of cells in the G2/M phase of the cell cycle and thus exhibited a larger amount of DNA content.

#### Cellular invasion assay

Henle-407 intestinal epithelial cells were infected with wild-type *S*. Typhi Quailes or the toxin-deficient SB6000 derivative for 2 h at three multiplicity of infection (MOI) levels (50,100 and 500). Cells were washed and gentamicin (50 μg ml^−1^) was added to the culture medium. After 2 h, cells were washed again, lysed and colony counts of both strains were determined by plating dilutions of the cell lysates. The invasive ability was expressed as the percentage of the bacterial inoculum that survived gentamicin treatment.

#### Comparison of the bacterial loads of *S*. Typhi Quailes ∆pltB, ∆pltA and ∆cdtB with *S*. Typhi Quailes *cdtB*^H160Q^, *pltB*^S35A^ and *pltA*^E133A^

Strains used for comparison of the bacterial loads of *S*. Typhi Quailes ∆pltB, ∆pltA and ∆cdtB with *S*. Typhi Quailes *cdtB*^H160Q^, *pltB*^S35A^ and *pltA*^E133A^ were derived from *S*. Typhi Quailes and were constructed by standard recombinant DNA techniques as described previously^16^. CmaH^−/−^ bloc3^−/−^ mice, which are susceptible to *S*. Typhi infection^32^, were intraperitoneally infected with equal numbers (10^5^ CFUs) of *S*. Typhi Quailes derivative mutant strains carrying either deletions in the *pltB*, *pltA* and *cdtB* genes (*S*. Typhi Quailes ∆*pltB*, ∆*pltA* and ∆*cdtB*) or expressing an inactivated version of typhoid toxin by virtue of catalytic mutations in its active subunits PltA and CdtB and a mutation in the receptor-binding site of PltB (*S*. Typhi Quailes *cdtB*^H160Q^, *pltB*^S35A^ and *pltA*^E133A^). The strains were alternatively marked by a chloramphenicol (*cmR*) or kanamycin (*kanR*) resistance genes, as indicated, inserted within the *STY4607* gene, which previous studies have shown not to affect virulence^32^. All animal experiments were conducted in accordance with protocols approved by Yale University’s Institutional Animal Care and Use Committee. Seven-to-ten week old cmaH^−/−^ bloc3^−/−^ mice were injected intraperitoneally with 10^5^ CFUs each of the two strains. The inoculum was plated to confirm the equivalent ratio of the bacterial strains. Mice were killed at day 5 post-infection and the CFUs of each strain in the spleens of infected animals were determined by plating on LB plates containing chloramphenicol (30 µg ml^−1^) or kanamycin (50 µg ml^−1^).

Phenotypic characterization of the wild-type and TN strains comprised growth characteristics in liquid culture, agglutination, cellular invasion assays and cell intoxication assays^4^. Whole-genome sequencing using both the MiSeq (Illumina) and PacBio (Pacific Biosciences) platforms was performed by the Wellcome Sanger Institute (Hinxton). DNA for MiSeq sequencing was extracted using the Wizard Genomic DNA purification kit according to the manufacturer’s instructions^33^. Sequence reads were assembled using HGAP v.3 of the SMRT analysis software v.2.3.0 (Supplementary Information).

### Establishment of challenge dose

Challenge agents were prepared in batches for a maximum of six participants at any one time. All work was performed in the containment level 3 facility at the Centre for Clinical Vaccinology and Tropical Medicine (Oxford) in a class II biological safety hood dedicated for challenge agent preparation.

Two GMP master stock vials of typhoid toxin-negative *S*. Typhi TN strain (BPR–1218-00, lot 1977; cell concentration 1 × 10^6^) or wild-type *S*. Typhi Quailes strain (BPR–1218-00, lot 1977; cell concentration 9.8 × 10^5^) were selected at random from stocks stored in a −80 °C freezer. Vials were thawed at room temperature for approximately 10 min and mixed by vortexing. The contents of two GMP master stock vials were transferred to a master stock tube and mixed for 6–10 s by vortexing. A 1:10 dilution in sodium bicarbonate was performed by transferring 1,600 μl from the tube labeled ‘master stock’ to a fresh 50 ml falcon labeled as ‘master stock 1:10’. To create the challenge inoculum of the toxin-negative strain, 1.74 ml from the ‘master stock 1:10 dilution’ was transferred to a sterile culture flask containing 4.2 g sodium bicarbonate dissolved in 240 ml bottled mineral water (‘challenge flask’). The challenge inoculum for the wild-type strain was generated by transferring 1.85 ml from the 1:10 dilution into an equivalent challenge flask. The challenge agents were then prepared by transferring 30 ml from the challenge flask to prelabelled 50 ml falcon tubes, sealed and stored on ice.

The challenge dose was confirmed by pipetting 200 μl from the challenge dose onto six Tryptone Soya Agar plates (code no. PO0163A; Oxoid). The bacterial suspension was spread over the source of the agar using an L-shaped spreader and cultured in an incubator overnight at 37 °C, 5% CO_2_. On the following day, colonies were manually counted and checked by a second operator. The CFUs of the challenge inoculum were calculated by multiplying the mean of the CFU counts for the plates by the dilution factor of the volume plated (×150 for a total challenge inoculum of 30 ml for a plating of 0.2 ml (30/0.2 = ×150)).

Sodium bicarbonate was prepared by dissolving 2.1 g sodium bicarbonate in 120 ml bottled mineral water.

### Participant characteristics

Healthy adults aged 18–60 years, without prior residency in an enteric fever endemic country for ≥6 months, were considered eligible for enrollment. Key exclusion criteria included significant medical, surgical or psychiatric history and gallbladder disease. A full description of the inclusion and exclusion criteria is provided in the Nature Research Reporting Summary.

### Randomization and masking

Participants were randomized 1:1 to challenge with either wild-type strain *S*. Typhi or toxin-negative strain *S*. Typhi (TN) in varying block sizes. Anti-Vi IgG was measured at screening using a commercial ELISA kit (VaccZyme; The Binding Site Ltd) according to the manufacturer’s instructions^14^. Randomization was stratified by anti-Vi IgG measured (low (<7.4 EU ml^−1^) or high (≥7.4 EU ml^−1^)). The exception was a sentinel group of two participants who were randomized 1:1 to receive the wild-type strain or TN knockout strain using a block size of two. Randomization was performed at the prechallenge visit, one week before challenge. We generated a randomization list in STATA v.14.2 (StataCorp), which was implemented in the computerized randomization software Sortition (Nuffield Department of Primary Care, Clinical Trials Unit, University of Oxford), which matched a masked allocation group to each participant. The software generated a randomization number, corresponding to the challenge allocation group. A locked, challenge agent randomization allocation list was maintained by the study statistician and unblinded laboratory team responsible for challenge agent preparation.

The study was conducted double-blind from the time of randomization until participant unblinding, such that participants, and clinical or laboratory staff undertaking follow-up procedures, were unaware of challenge agent allocation. Both wild-type and TN strains were prepared suspended in sodium bicarbonate and had an indistinguishable appearance (transparent, colorless liquid).

### Procedures

Participants fasted for 90 min before challenge. Two minutes before challenge, participants drank a sodium bicarbonate solution (2.1 g 120 ml^−1^) to neutralize stomach acid. The oral challenge inoculum was administered suspended in sodium bicarbonate (0.53 g 30 ml^−1^) and was kept on ice before administration within 3 h of preparation. Participants were observed for 90 min post-challenge. The challenge dose administered was 1–5 × 10^4^ CFUs calculated as described previously^7,13,14^. Participants attended the clinical site 12 h after challenge and then daily for 14 d, as described previously^7^. Daily visits comprised continued consent check, oral temperature measurement, heart rate and blood pressure measurement and sample collection, as outlined in the study protocol.

Solicited symptoms and twice-daily temperature measurements were recorded in an electronic diary for 21 d after challenge. Symptoms were categorized as not present, mild, moderate or severe (Supplementary Information).

Antibiotic treatment was initiated on fulfillment of composite diagnostic criteria or at day 14 for those without illness. First-line treatment was oral ciprofloxacin 500 mg twice daily for 14 d.

### Outcomes

The primary objective of this study was to compare the proportion of participants meeting the composite diagnostic end point for typhoid fever (attack rate) following oral challenge with (1–5) × 10^4^ CFUs wild-type *S*. Typhi Quailes strain, compared to challenge with (1–5) × 10^4^ CFUs of a typhoid toxin-deficient isogenic mutant of *S*. Typhi Quailes strain SB6000 (TN). The composite diagnostic end point for typhoid fever was defined as a temperature ≥38 °C persisting for ≥12 h and/or *S*. Typhi bacteremia collected ≥72 h after oral challenge.

Secondary end points were: mode of diagnosis; time to typhoid diagnosis; time to first temperature ≥38 °C; fever clearance time; time to bacteremia; duration of bacteremia; and quantitative blood culture (for definitions, see the Life Sciences Reporting Summary). Descriptive end points included: severe adverse events; solicited symptom profiles; proportion of participants meeting the criteria for severe enteric fever; hematological and biochemical measures; plasma cytokine profiles; pattern of bacteremia; and pattern of stool shedding (see Life Sciences Reporting Summary).

Stool samples for culture, blood samples for culture (10 ml), and hematological and biochemical testing were processed by the local hospital’s accredited pathology laboratory as described previously^7^.

### Criteria for severe enteric fever

Severe enteric fever was defined as participants meeting any of the following criteria: oral temperature >40 °C; systolic blood pressure <85 mmHg; significant lethargy or confusion; gastrointestinal bleeding; gastrointestinal perforation; or any grade 4 laboratory abnormality^34^.

### Ex vivo ASC enzyme-linked immune absorbent spot (ELISpot)

Ex vivo IgG-, IgA- and IgM-producing ASC responses against O- and H-antigen were measured at baseline and 24–48 h after typhoid diagnosis in those meeting the diagnostic criteria as described previously^13^.

Multiscreen filtration ELISpot plates (catalog no. MAHAS4510; Merck Millipore) were coated with *S*. Typhi O9:LPS, *S*. Typhi Hd antigen (University of Maryland) and Pan goat anti-human immunoglobulin (catalog no. H17000; Invitrogen) each at a final concentration of 10 μg ml^−1^ in carbonate-bicarbonate buffer and incubated overnight at 4 °C. Plates were blocked with 200 μl per well of R10 medium for 1 h before use at 37 °C, 5% CO_2_. Peripheral blood mononuclear cells (PBMCs) were separated using ACCUSPIN tubes (Sigma-Aldrich), counted and resuspended in R10 media. PBMCs at a concentration of 2.5 × 10^5^ were added in duplicate to the ELISpot plate (100 μl per well) and incubated overnight at 37 °C, 5% CO_2_. Plates were washed four times with PBS-0.25% Tween, once with PBS and soaked with PBS for 5 min. Goat anti-human IgG, IgA and IgM secondary antibodies conjugated to alkaline phosphatase (catalog nos. 401442, 401132 and 401902, respectively; Sigma-Aldrich) were diluted to 1:5,000 in PBS/FBS and incubated for 4 h at room temperature. After incubation, the plates were washed five times with PBS-0.25% Tween and four times with dsH_2_O. Alkaline phosphatase substrate (catalog no. 170-6432; Bio-Rad) was added at 50 μl per well, allowed to develop over approximately 10 min and stopped with dsH_2_O as spots began to develop.

ELISpot plates were read using an automated ELISpot reader (ELR03/ELR030408215; Autoimmun Diagnostika) and the AID ELISpot software v.5.0. Study- and antigen-specific count settings for spot intensity, size and gradient were applied to the plate counts and manually verified to remove artifacts. Raw counts (spots per 2.5 × 10^5^ PBMCs) were averaged across duplicate wells and multiplied by four to give the number of spot-forming units (SFUs) per 10^6^ PBMCs.

### Fluorospots

Measurements were taken from frozen PBMCs collected at baseline, and on day 14 and 28 post-challenge. Precoated plates (catalog no. FSP-010308-10; Mabtech) were blocked before adding 50 µl per well toxin peptide pools consisting of 15-mer sequences with 11-amino acid overlaps and covering the sequence of proteins CdtB, PltA and PltB (thinkpeptides). The peptides were dissolved in 100% DMSO (Sigma-Aldrich) and arranged in three pools. Concentration was adjusted at 0.6 mg ml^−1^ and used in the fluorospot assay at a final concentration of 3 µg ml^−1^ of each peptide. DMSO and concanavalin A (Sigma-Aldrich) were used as negative and positive controls, respectively. After defrosting and resting for 1 h, 50 µl per well of PBMCs were added to the peptide wells at a concentration of 4 × 10^6^ cells ml^−1^ in triplicate and incubated overnight at 37 °C, 5% CO_2_, 95% humidity. Detection of spots was carried out according to the manufacturer’s instructions (Mabtech) and analyzed with the iSpot EliSpot reader (Autoimmun Diagnostika).

### Plasma cytokine analysis

Plasma was isolated from heparinized blood by centrifugation. Protease inhibitor was added in a 1:40 dilution before storage at −80 °C. Longitudinal cytokine quantification was carried out for all 40 challenged participants by the Human Immune Monitoring Center at Stanford University using a 62-plex Luminex system (brain-derived neurotrophic factor, beta-nerve growth factor, CD40 ligand, epidermal growth factor, ENA-78 (CXCL5), eotaxin, fibroblast growth factor 2, granulocyte-colony-stimulating factor, granulocyte-macrophage colony-stimulating factor, GRO-α (CXCL1), hepatocyte growth factor, IFN-α, IFN-β, IFN-γ, IL-1-α, IL-1-β, IL-10, IL-12p40, IL-12p70, IL-13, IL-15, IL-17A, IL-17F, IL-18, IL-1RA, IL-2, IL-21, IL-22, IL-23, IL-27, IL-31, IL-4, IL-5, IL-6, IL-7, IL-8, IL-9, IP-10, leptin, leukemia inhibitory factor, macrophage colony-stimulating factor 1, monocyte chemoattractant protein 1 (MCP-1), MCP-3, MIG, macrophage inflammatory protein-1-α (MIP-1-α), MIP-1-β, plasminogen activator inhibitor 1, platelet-derived growth factor subunit B, RANTES, resistin, stem cell factor, stromal cell-derived factor 1, soluble Fas ligand, soluble intercellular adhesion molecule 1, soluble vascular cell adhesion protein 1, transforming growth factor-α (TGF-α), TGF-β, tumor necrosis factor-α (TNF-α), TNF-β, tumor necrosis factor ligand superfamily member 10, vascular endothelial growth factor A (VEGF-A) and VEGF-D). Samples were run in duplicate and the mean fluorescence intensity (MFI) of duplicates was used for analysis. To minimize plate-to-plate variation, samples across time points for each individual were run on the same plates and each plate contained an equal mix of individuals allocated to wild-type or toxin-negative challenge. Control beads (CHEX 1–4) and control sera were used per plate.

The MFI of all samples were examined by principal component analysis to confirm consistency between duplicates and identify outliers. One participant was excluded on this basis. Duplicates were then averaged and the MFI quantile normalized. Significance testing was performed using linear modeling in limma v3.34.9 (ref. ^35^), incorporating plate, dose and sex as covariates. *P* values were corrected for multiple testing using the Benjamin–Hochberg correction. Hierarchal clustering was carried out based on Euclidean distance.

### Sample size

The sample size was dictated primarily by the number of participants that could be feasibly enrolled within the time frame and budget of the study; therefore, it represents a convenience sample. Assuming typhoid toxin is central to the clinical presentation of acute typhoid fever, it was anticipated that the attack rate following challenge with the TN strain would be reduced compared with the wild-type strain, although the effect size was unknown. Assuming an attack rate of 65% following wild-type challenge (as observed in previous studies) and 50% attack rate following TN challenge, and accounting for a 10% dropout, 20 participants in each group had 95% CIs for attack rate of 41–85% in the wild-type group and 27–73% in the TN group. Twenty participants per arm provided 95% power to detect an absolute reduction in attack rate of 55% (65% with the wild-type strain versus 10% with the TN strain, corresponding to an 85% relative risk reduction) and 80% power to detect an absolute reduction in attack rate of 47% (65% with the *S*. Typhi wild-type strain versus 18% with the *S*. Typhi toxin-negative strain, corresponding to a 72% relative risk reduction) based on Fisher’s exact test with 5% alpha.

### Statistical considerations

Attack rates and 95% CIs were calculated for each challenge group for the per-protocol population (that is, participants who completed the 14-d challenge period) as the primary end point. All participants were included in the analyses if they were successfully challenged on day 0 and had at least one post-challenge assessment. The difference in attack rate (and other categorical variables) between naïve and rechallenge groups was tested using Fisher’s exact test. Time-to-event data were summarized using the Kaplan–Meier method, with participants censored at day 14. Group comparisons were performed using a log-rank test. Continuous variables were compared using the Mann–Whitney *U*-test for unpaired samples and the Wilcoxon signed-rank test for paired samples. All statistical tests were two-sided.

Paired samples across time points were compared using the Wilcoxon signed-rank test. Comparisons between groups were performed using the Mann–Whitney *U*-test. ELISpot/FluoroSpot data were log_10_-transformed to approximate a normal distribution; wells with no spots were assigned an arbitrary value of 0.5, corresponding to half the lower limit of detection. Raw counts were averaged across replicate wells. The number of background spots detected in blank wells were subtracted from the test samples to give the final cell count per sample.

Clinical data were recorded on a web-based database (OpenClinica Enterprise v3.13). Symptom and ELISpot were extracted using Microsoft Excel. Data analysis was performed using R v.3.4.4. Variables were normalized by *z*-score before inclusion in the principal component analysis, which was performed using the FactoMineR package v1.41 (ref. ^36^).

### Approvals

The OVG2016/03 study was sponsored by the University of Oxford (Clinical Trials & Research Governance). Ethical approvals for the primary protocol, and any study amendments, were obtained from the South Central-Oxford A Research Ethics Committee (16/SC/0358). In the UK, legislation governing the deliberate release of genetically modified organisms is currently provided by the Environmental Protection Act 1990, sections 111 and 112 (ref. ^37^), and the Genetically Modified Organisms (Deliberate Release) Regulations 2002 (ref. ^38^). Approvals for deliberate release of the genetically modified strain of *S*. Typhi were obtained from the United Kingdom Department for Environment, Food & Rural Affairs (16/R48/01) (ref. ^39^). The study was registered with clinicaltrials.gov (NCT03067961) and was performed according to the provisions of the Declaration of Helsinki (2013) and Good Clinical Practice guidelines. This work is licensed under the Creative Commons Attribution 4.0 International License.

### Reporting Summary

Further information on research design is available in the Nature Research Reporting Summary linked to this article.

## Online content

Any methods, additional references, Nature Research reporting summaries, source data, statements of code and data availability and associated accession codes are available at 10.1038/s41591-019-0505-4.

## Supplementary information

Supplementary Information Supplementsary Method Tables 1–3, Supplementary Figs. 1 and 2; Details of strain genotyping

Reporting summary

Supplementary Tables Supplementary Data Tables 1–6

Study Protocol Study protocol

## Data Availability

The datasets generated and/or analyzed during the current study are attached. Any additional data are available from the corresponding author. No participant identifiable information will be disclosed. The raw sequence reads for the wild-type and TN strains used in the challenge are available under accession nos. ERS3381923 and ERS3381927.

## Source data

Source Data Fig. 1 Statistical Source Data
Source Data Fig. 2 Statistical Source Data
Source Data Fig. 3 Statistical Source Data
Source Data Fig. 4 Statistical Source Data
Source Data Extended Data Fig. 2 Statistical Source Data
Source Data Extended Data Fig. 3 Statistical Source Data
Source Data Extended Data Fig. 4 Statistical Source Data
Source Data Extended Data Fig. 5 Statistical Source Data
Source Data Extended Data Fig. 6 Statistical Source Data
