# Supplementary material for: Investigation of the role of typhoid toxin in acute typhoid fever in a human challenge model
Source: Nat Med. 2019 Jul 3;25(7):1082–8. doi: 10.1038/s41591-019-0505-4 (PMC6892374; doi:10.1038/s41591-019-0505-4)
Supplement: Supplementary file 1 — Supplementsary Method Tables 1–3, Supplementary Figs. 1 and 2; Details of strain genotyping [file 41591_2019_505_MOESM1_ESM.pdf]

nature medicine

SUPPLEMENTARY INFORMATION

https://doi.org/10.1038/s41591-019-0505-4

In the format provided by the authors and unedited.

Investigation of the role of typhoid toxin in acute typhoid fever in a human challenge model

Malick M. Gibani[1,2*], Elizabeth Jones[1], Amber Barton[1], Celina Jin[1], Juliette Meek[1], Susana Camara[1], Ushma Galal[3], Eva Heinz[4,5], Yael Rosenberg-Hasson[6], Gerlinde Obermoser[6], Claire Jones[1], Danielle Campbell[1], Charlotte Black[1], Helena Thomaides-Brears[1], Christopher Darlow[1], Christina Dold[1], Laura Silva-Reyes[1], Luke Blackwell[1], Maria Lara-Tejero[7], Xuyao Jiao[7], Gabrielle Stack[7], Christoph J. Blohmke[1], Jennifer Hill[1], Brian Angus[8], Gordon Dougan[4,9], Jorge Galán[7] and Andrew J. Pollard[1]

[1]Oxford Vaccine Group, Department of Paediatrics, University of Oxford and the NIHR Oxford Biomedical Research Centre, Oxford, UK. [2]Department of Medicine, Imperial College London, London, UK. [3]Nuffield Department of Primary Care Health Sciences, Clinical Trials Unit, University of Oxford, Oxford, UK. [4]Wellcome Sanger Institute, Wellcome Genome Campus, Hinxton, UK. [5]Department of Vector Biology, Liverpool School of Tropical Medicine, Liverpool, UK. [6]Human Immune Monitoring Center, Institute for Immunity, Transplantation and Infection, Stanford University, Stanford, CA, USA. [7]Department of Microbial Pathogenesis, Yale University School of Medicine, New Haven, CT, USA. [8]Nuffield Department of Medicine, University of Oxford, Oxford, UK. [9]Department of Medicine, University of Cambridge, Hinxton, UK. *e-mail: malick.gibani@paediatrics.ox.ac.uk**Open Access** This article is licensed under a Creative Commons Attribution 4.0 International License, which permits use, sharing, adaptation, distribution and reproduction in any medium or format, as long as you give appropriate credit to the original author(s) and the source, provide a link to the Creative Commons license, and indicate if changes were made. The images or other third party material in this article are included in the article's Creative Commons license, unless indicated otherwise in a credit line to the material. If material is not included in the article's Creative Commons license and your intended use is not permitted by statutory regulation or exceeds the permitted use, you will need to obtain permission directly from the copyright holder. To view a copy of this license, visit http://creativecommons.org/licenses/by/4.0/.

SUPPLEMENTARY INFORMATION

Solicited Symptoms & Expected Adverse Events of Enteric Fever	Grade 0	Grade 1	Grade 2	Grade 3	Grade 4
Headache	Not present	Present but no interference with activity	Some interference with activity	Significant; any use of codeine phosphate or prevents daily activity	Hospital visit required
Malaise	Not present	Present but no interference with activity	Some interference with activity	Significant; any use of codeine phosphate or prevents daily activity	Hospital visit required
Anorexia	Not present	Eat less than normal for 1-2 meals	Miss 1-2 meals completely	Miss all meals completely	Hospital visit required
Rash	Not present	Present but no interference with activity	Some interference with activity	Significant or prevents daily activity	Hospital visit required
Constipation	Not present	Present but no interference with activity	Some interference with activity	Significant or prevents daily activity	Hospital visit required
Diarrhoea	Not present	3 – 4 loose stools in 24 hours (≥200mls volume)	5 – 6 loose stools in 24 hours (≥200mls volume)	>6 loose stools in 24 hours (≥200mls volume)	Hospital visit required
Abdominal pain/distension	Not present	Present but no interference with activity	Some interference with activity	Significant or prevents daily activity	Hospital visit required
Myalgia	Not present	Present but no interference with activity	Some interference with activity	Significant or prevents daily activity	Hospital visit required
Arthralgia	Not present	Present but no interference with activity	Some interference with activity	Significant or prevents daily activity	Hospital visit required
Cough	Not present	Present but no interference with activity	Some interference with activity	Significant or prevents daily activity	Hospital visit required
Nausea and/or vomiting	Not present	Present but no interference with activity or 1 – 2 episodes in 24 hours	Some interference with activity or more than 2 episodes in 24 hours	Significant or prevents daily activity	Hospital visit required

Supplementary Methods Table 1 - Grading of solicited symptoms.

Parameter				
	Grade 1	Grade 2	Grade 3	Grade 4
Haemoglobin: decrease from baseline value (gm/dl)	< 1.5	1.5-2.0	2.1-5.0	>5
White cell count: elevated (cell/mm3)	10,800–15,000	15,001–20,000	20,001–25,000	>25,000
White cell count: depressed (cells/mm3)	2500-3500	1500-2499	1000-1499	<1000
Neutrophil count (cells/mm3)	1500-2000	1000-1499	500-999	<500
Lymphocyte count (cells/mm3)	750-1000	500-749	250-499	<249
Platelets (cells/mm3)	125,000-140,000	100,000-124,000	25,000-99,000	<25,000
Sodium: hyponatraemia (mmol/L)	132–134	130–131	125–129	<125
Sodium: hypernatraemia (mmol/L)	144–145	146–147	148–150	>150
Potassium: hyperkalaemia (mmol/L)	5.1–5.2	5.3–5.4	5.5–5.6	>5.6
Potassium: hypokalaemia (mmol/L)	3.5–3.6	3.3–3.4	3.1–3.2	<3.1
Urea (mmol/L)	8.2–8.9	9.0–11	>11	RRT
Creatinine (µmol/L)	132-150	151-176	177-221	>221 or RRT
ALT and/or AST (IU/L)	1.1–2.5 x ULN	>2.6–5.0 x ULN	5.1-10 x ULN	>10 x ULN
Bilirubin, with increase in LFTs (umol/L)	1.1–1.25 x ULN	1.26–1.5 x ULN	1.51–1.75 x ULN	>1.75 x ULN
Bilirubin, with normal LFTs (umol/L)	1.1–1.5 x ULN	1.6–2.0 x ULN	2.0–3.0 x ULN	>3.0 x ULN
Alkaline phosphatase (U/L)	1.1–2.0 x ULN	2.1–3.0 x ULN	3.1–10 x ULN	>10 x ULN
Albumin: hypoalbuminaemia (g/L)	28–31	25–27	<25	Not applicable
C-reactive protein	>10-30	31-100	100-200	>200

Supplementary Methods Table 2 – Grading of laboratory parameters

Observation	Grade 0	Grade 1	Grade 2	Grade 3
Oral temperature (°C)	35.5 – 37.5	37.6 – 38.0	38.1 – 39.0	>39
Tachycardia (beats/min)	55-100	101-115	116-130	>130
Bradycardia (beats/min)	55-100	50-54	45-49	<45
Systolic hypertension (mmHg)	90-140	141-150	151-155	>155
Diastolic hypertension (mmHg)	90-140	91-95	96-100	>100
Systolic hypotension (mmHg)	90-140	85-89	80-84	<80

Supplementary Methods Table 3 – Grading of clinical observations

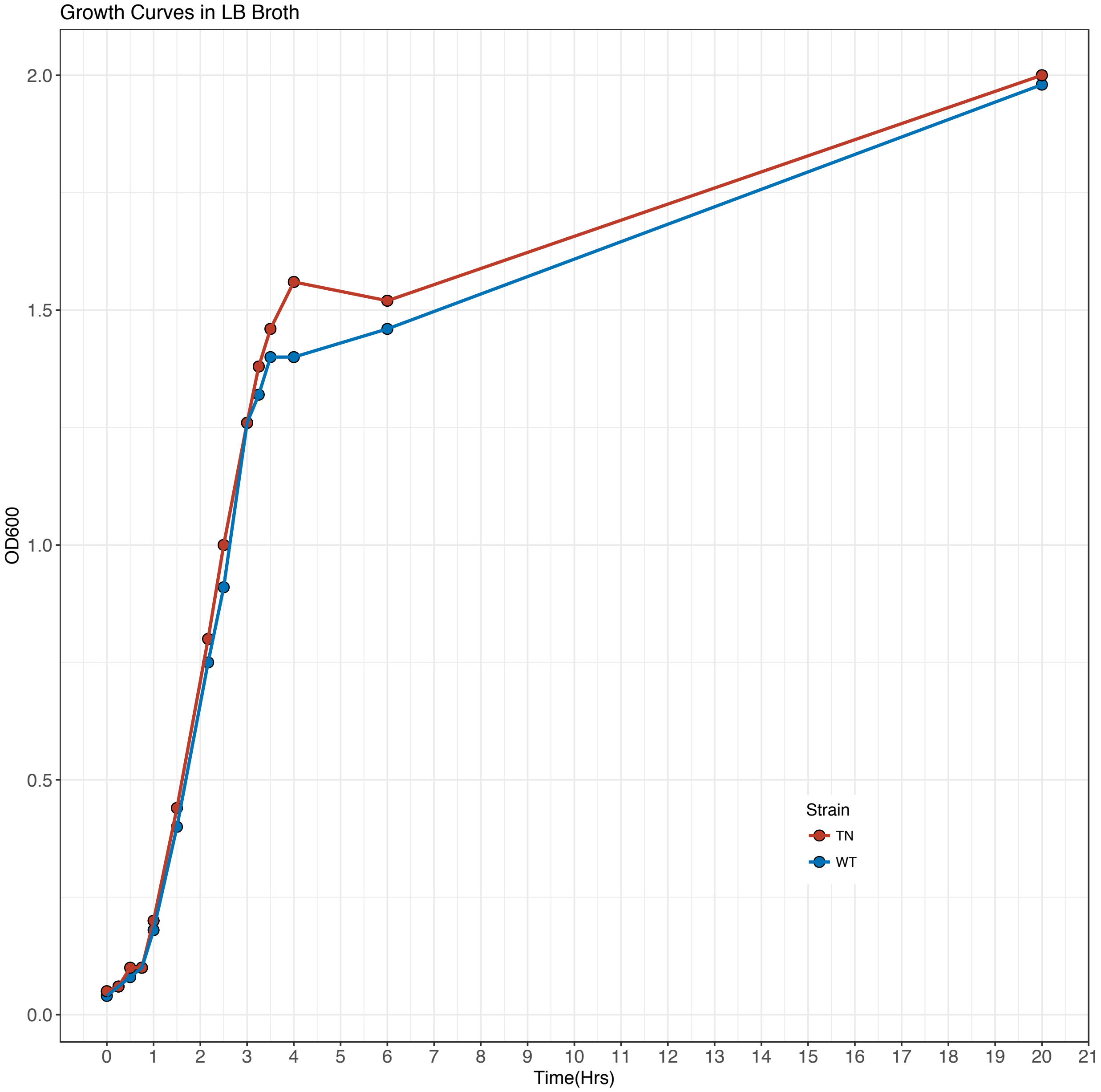

Supplementary Figure 1 : Growth curves of wild-type (WT) and typhoid-toxin deficient (TN) S. Typhi*. Data points show mean OD_{600} of duplicate samples. Isolates were inoculated into 10ml of LB and grown overnight in a shaking incubator at 200-220rpm and 37℃. Data points represent mean values from two independent experiments conducted in duplicate..*

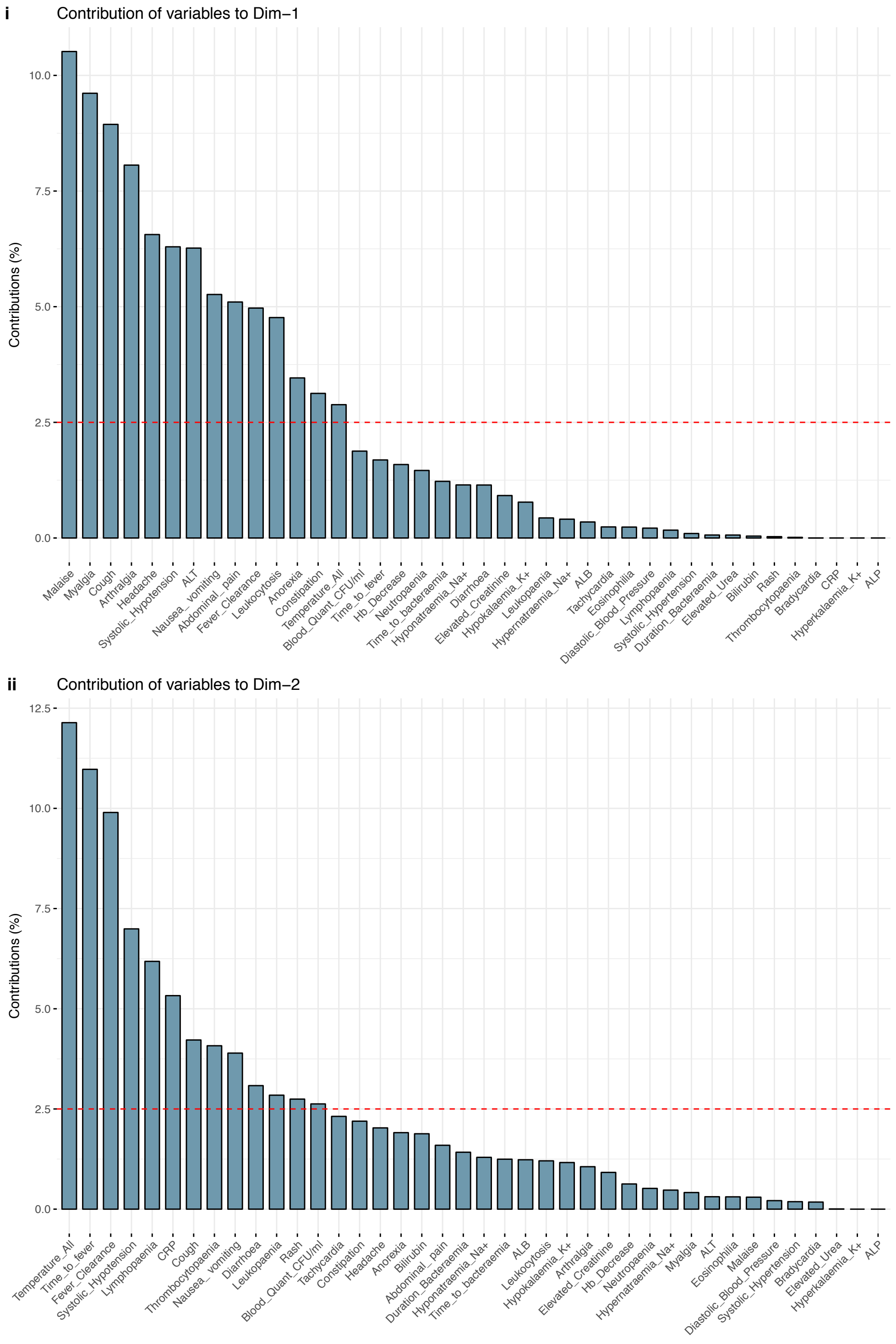

Supplementary Figure 2 - Contributions of individual variables to PCA of disease severity (i)Principal component 1 and (ii) Principal component 2 in participants diagnosed with typhoid fever after challenge, expressed as percentage contribution. *Variables listed on the X-axis represent variables included in the original principal component analysis. Red dashed line indicates the expected average contribution if the contribution were uniform, corresponding to 1/n(variables). Variables over the cut-off are considered important contributors to that principal component* [1.]

Strain genotyping

Five samples were submitted to sequencing:

1) Oxford w/t *Salmonella* Typhi Quailes strain (previously used for challenge studies)
2) Pre-GMP w/t *Salmonella* Typhi Quailes
3) Post-GMP w/t *Salmonella* Typhi Quailes
4) Pre-GMP *Salmonella* Typhi typhoid-toxin k/o
5) Post-GMP *Salmonella* Typhi typhoid-toxin k/o

Sequencing and analysis were performed at the Wellcome Trust Sanger Institute (Hinxton, UK). DNA for MiSeq sequencing was extracted using the Wizard® Genomic DNA purification kit according to manufacturer's instructions[2]. DNA for PacBio sequencing was extracted using a protocol supplied by the Wellcome Trust Sanger institute. The samples were sequenced using MiSeq and on the PacBio RSII using P6/C4 sequencing chemistry, the library was made using the SMRTbell Template Prep Kit 1.0.

Computational analyses

The automated pacbio assembly pipeline resulted in one large contig per sample, as well as 1-3 additional small fragments, and the one fragment was not always circularised. The additional sample fragments (additional contigs) were investigated for their potential as plasmids; all fragments were concluded to be fragments of the genome and encoded mostly bacteriophage proteins. To assess the coverage of these additional fragments, the reads were mapped against the original assembly; using both the corrected pacbio as well as the miseq reads. This revealed a low or in some areas equal coverage of the additional fragments as to be expected from fragments of the genome. This furthermore highlighted a large collapsed repeat region (large peak area with 2x coverage compared to the remaining sequence, indicating that this should be present 2x in the genome). This large collapsed repeat was also confirmed when using pilon [3] a tool to correct small errors resulting from long reads (pacbio) with short (e.g. Miseq) reads; which does not correct large repeats, but reports these. After several unsuccessful attempts to solve this repeat using different automated assembly strategies (canu[4]; hypridspades[5]); this repeat region was resolved manually using the assembly program gap5[6]. The quality of the improved assembly was controlled again by mapping the reads against this manually polished assembly and creating a final gap5 database, which showed an even coverage across the genome. This was repeated for strains 1-3 and 5; strain 4 included contigs broken at variable regions which renders it challenging to close, and the automated assembly containing 4 contigs was therefore used for this strain for further analysis. Throughout the analysis, two highly variable regions encoding phage tail proteins were noticed.

With the exception of three regions discussed below, no differences between the wild type strains and the knockout strains could be observed using tools to detect single-nucleotide polymorphisms (SNPs) or small insertions/deletions (INDELs), as well as assessing large-scale genome rearrangements, and comparing the assemblies. Variations in the form of ambiguous base calls detected with the SNP calling tools could be detected in an equal amount across all samples, an accumulation was observed in the phage region discussed below.

The other three variations included two phage regions, as well as one stretch of 8-basepair repeats in an intergenic region. This however showed variation across all samples, with 18 copies in strains 3, 4 and 5 and 17 copies in strains 1 and 2. The repetitive nature and ambiguity in the raw reads, as well as the variation in the wild type samples, indicates that this is not a consequence of the knockout, but rather natural variation or technical issues which are known to occur with repeat regions.

The two phage regions showed variation in all samples in four phage tail proteins, including in the raw sequencing data. Focusing on the manual assemblies between strain 3 and 5 (both post-GMT) where all variation is summed in one consensus sequence, the rearrangement results in the change of sequence of one protein, which gets split into two open reading frames in sample 5 but is only one in sample 3; and two proteins get inverted. All these proteins are annotated as phage tail proteins, searches against the NCBI result in hits in Salmonella bacteriophage tail proteins. The assemblies also differed within the wild type samples (1/2/3) in this region, and are the second reason for additional contigs formed by the automatic assembly programs. The second phage region resulted in similar changes, again concerning phage tail proteins, where two or three are inverted. In this region, samples 2 and 5 show identical sequences, as do samples 1 and 3, indicating that this is normal variation in this strain.

Methods: Large-scale rearrangements were investigated using the genome comparison programs ACT [7] and Mauve[8]. To look for single nucleotide polymorphisms (SNPs)/small insertions or deletions (INDELs), an in-house SNP-calling script was used. Uncertain bases were also assessed visually; the pacbio reads were also mapped using bwa mem[9] and mapping across the whole assembly was assessed visually. MiSeq assemblies were compared to the manually cleaned wild type assembly using abacas[10], and the contigs from the Miseq data which did not match the wild type assembly were assessed manually. This again only included variable or repetitive regions of the genome (phage, 16S/23S regions), and no indication for additional sequence could be observed. Comparison of the assemblies was also performed using parsnp and garlic from the harvest suite[11] as well as blast.

Detailed methods:

Genome assembly

The automated pacbio pipeline assemblies were performed as follows: Sequence reads were assembled using HGAP v3[12] of the SMRT analysis software v2.3.0[13]. The fold coverage to target when picking the minimum fragment length for assembly was set to 30 and the approximate genome size was set to 3Mbp. The assembly was circularized using Circlator v1.1.3[14] and the pre-assembled reads (also known as corrected reads). Finally, the circularized assembly was polished using the PacBio RS_Resequencing protocol and Quiver v1 of the SMRT analysis software v2.3.0[13]. Automated annotation, as well as annotation steps on manual assemblies, was performed using PROKKA v1.11[15] and a genus specific databases from RefSeq[16].

SNP/Indel calling

The in-house script uses SMALT[17] v0.7.4 to map reads against a selected reference including randomly mapping the repeats and using the GATK indel alignment option. Variation detection was performed using samtools mpileup v0.1.19[18] and bcftools v0.1.19 to produce a BCF file of all sites and all variant sites. The SNPs were called against the wild type assembly, which resulted in no SNPs except in the toxin knockout region from the two knockout strains. The results were assessed visually as well, by opening the reference in Artemis, as well as opening the bam file containing the mapped reads of the respective strain and the full bcf file, turning the 'variant sites' option in Artemis on to see all sites, irrespective of variation or not, and ambiguous bases were assessed. These indicated potential sequence variation in the phage region as discussed.

Building a gap5 database

For manual completion of the assemblies a gap5 database was built[6]. For this, a bam file based on the assembly was generated (https://github.com/sanger-pathogens/Fastaq/blob/master/pyfastaq/runners/to_tiling_bam.py). This bam file was merged with the other bam files using 'samtools merge', and an index based on the merged bam file was generated using the gap5 database builder tg_index command, and the database started using the gap5

command, and the consensus assembly exported after manual improvement in gap5. Mapping of the respective reads back to control the consensus assemblies was performed with bwa mem [7], adding the "-x pacbio" flag for mapping pacbio reads.

The raw sequence reads are available under accessions ERS3381923 (sample 1 Oxford w/t), ERS3381924 (sample 2 w/t Pre-GMT), ERS3381925 (sample 3 w/t Post-GMT), ERS3381926 (sample 4 k/o Pre-GMT), and ERS3381927 (sample 5 k/o Post-GMT). Manually refined hybrid assemblies as described above are given for the wild type strain (sample 2 w/t Pre-GMT) under accession GCA_901457615 and for the knock-out strain (sample 5 k/o Post-GMT) under accession GCA_901457625.

Reference:

1. PCA - Principal Component Analysis Essentials - Articles - STHDA. Available at: http://www.sthda.com/english/articles/31-principal-component-methods-in-r-practical-guide/112-pca-principal-component-analysis-essentials/. (Accessed: 19th June 2018)
2. Promega. Isolation of Genomic DNA from Whole Blood Wizard ® Genomic DNA Purification Kit Lysis Protein DNA Sample Solution Precipitation Rehydration Size Cell Nuclei. Available at: www.promega.com. (Accessed: 21st June 2018)
3. Walker, B. J. *et al.* Pilon: An Integrated Tool for Comprehensive Microbial Variant Detection and Genome Assembly Improvement. *PLoS One* **9**, e112963 (2014).
4. Koren, S. *et al.* Canu: scalable and accurate long-read assembly via adaptive *k* -mer weighting and repeat separation. *Genome Res.* **27**, 722–736 (2017).
5. Antipov, D., Korobeynikov, A., McLean, J. S. & Pevzner, P. A. hybridSPAdes: an algorithm for hybrid assembly of short and long reads. *Bioinformatics* **32**, 1009–1015 (2016).
6. Bonfield, J. K. & Whitwham, A. Gap5—editing the billion fragment sequence assembly. *Bioinformatics* **26**, 1699–1703 (2010).
7. Carver, T. J. *et al.* ACT: the Artemis comparison tool. *Bioinformatics* **21**, 3422–3423 (2005).
8. Darling, A. C. E., Mau, B., Blattner, F. R. & Perna, N. T. Mauve: Multiple Alignment of Conserved Genomic Sequence With Rearrangements. *Genome Res.* **14**, 1394–1403 (2004).
9. Li, H. & Durbin, R. Fast and accurate short read alignment with Burrows-Wheeler transform. *Bioinformatics* **25**, 1754–1760 (2009).
10. Assefa, S., Keane, T. M., Otto, T. D., Newbold, C. & Berriman, M. ABACAS: algorithm-based automatic contiguation of assembled sequences. *Bioinformatics* **25**, 1968–1969 (2009).
11. Treangen, T. J., Ondov, B. D., Koren, S. & Phillippy, A. M. The Harvest suite for rapid core-genome alignment and visualization of thousands of intraspecific microbial genomes. *Genome Biol.* **15**, 524 (2014).
12. Chin, C.-S. *et al.* Nonhybrid, finished microbial genome assemblies from long-read SMRT sequencing data. *Nat. Methods* **10**, 563–569 (2013).
13. PacBio. SMRT-Analysis by PacificBiosciences. Available at: http://pacificbiosciences.github.io/SMRT-Analysis/. (Accessed: 19th May 2019)
14. Hunt, M. *et al.* Circlator: automated circularization of genome assemblies using long sequencing reads. *Genome Biol.* **16**, 294 (2015).
15. Seemann, T. Prokka: rapid prokaryotic genome annotation. *Bioinformatics* **30**, 2068–2069 (2014).
16. Pruitt, K. D., Tatusova, T., Brown, G. R. & Maglott, D. R. NCBI Reference Sequences (RefSeq): current status, new features and genome annotation policy. *Nucleic Acids Res.* **40**, D130-5 (2012).

17. WTSI. SMALT | Wellcome Sanger Institute. Available at: https://www.sanger.ac.uk/science/tools/smalt-0. (Accessed: 19th May 2019)
18. Li, H. *et al.* The Sequence Alignment/Map format and SAMtools. *Bioinformatics* **25**, 2078–9 (2009).
