# Supplementary material for: Investigation of the role of typhoid toxin in acute typhoid fever in a human challenge model
Source: Nat Med. 2019 Jul 3;25(7):1082–8. doi: 10.1038/s41591-019-0505-4 (PMC6892374; doi:10.1038/s41591-019-0505-4)
Supplement: Supplementary file 2 — Reporting summary [file 41591_2019_505_MOESM2_ESM.pdf]

nature research

Corresponding author(s): Malick M Gibani

Last updated by author(s): 5/20/2019

Reporting Summary

Nature Research wishes to improve the reproducibility of the work that we publish. This form provides structure for consistency and transparency in reporting. For further information on Nature Research policies, see Authors & Referees and the Editorial Policy Checklist.

Statistics

For all statistical analyses, confirm that the following items are present in the figure legend, table legend, main text, or Methods section.

n/a	Confirmed	
☐	☒	The exact sample size (*n*) for each experimental group/condition, given as a discrete number and unit of measurement
☐	☒	A statement on whether measurements were taken from distinct samples or whether the same sample was measured repeatedly
☐	☒	The statistical test(s) used AND whether they are one- or two-sided<br>*Only common tests should be described solely by name; describe more complex techniques in the Methods section.*
☐	☒	A description of all covariates tested
☐	☒	A description of any assumptions or corrections, such as tests of normality and adjustment for multiple comparisons
☐	☒	A full description of the statistical parameters including central tendency (e.g. means) or other basic estimates (e.g. regression coefficient) AND variation (e.g. standard deviation) or associated estimates of uncertainty (e.g. confidence intervals)
☐	☒	For null hypothesis testing, the test statistic (e.g. *F*, *t*, *r*) with confidence intervals, effect sizes, degrees of freedom and *P* value noted<br>*Give P values as exact values whenever suitable.*
☒	☐	For Bayesian analysis, information on the choice of priors and Markov chain Monte Carlo settings
☒	☐	For hierarchical and complex designs, identification of the appropriate level for tests and full reporting of outcomes
☒	☐	Estimates of effect sizes (e.g. Cohen's *d*, Pearson's *r*), indicating how they were calculated

Our web collection on statistics for biologists contains articles on many of the points above.

Software and code

Policy information about availability of computer code

Data collection	Clinical data were recorded on a web-based database (OpenClinica Enterprise). Symptom and ELISPOT/Flurospot were extracted using Microsoft Excel. ELISPOT plates were read using an automated ELISPOT reader (AID ELR03/ELR030408215) and AID ELISPOT software V5.0.

Data analysis

Data analysis was performed using R version 3.4.4. Plots were generated using the ggplot2 package. . The principal component analysis was performed using the FactoMineR package 37 with R version 3.4.4.
The randomisation list was generated in STATA version 14.2.
Randomisation was implemented using Sortition® (Oxford University Innovation Ltd & Nuffield Department of Primary Care, Clinical Trials Unit, University of Oxford).

Sequencing:
Sequence reads were assembled using HGAP v3 (PMID: 23644548) of the SMRT analysis software v2.3.0 (Available from: https://github.com/PacificBiosciences/SMRT-Analysis).
The assembly was circularized using Circlator v1.1.3 (PMID: 26714481).
The circularized assembly was polished using the PacBio RS_Resequencing protocol and Quiver v1 of the SMRT analysis software v2.3.0 (Available from: https://github.com/PacificBiosciences/SMRT-Analysis).
Automated annotation, as well as annotation steps on manual assemblies, was performed using PROKKA v1.11 [(PubMed PMID: 24642063) and a genus specific databases from RefSeq (PubMed PMID: 22121212).

SNP/Indel calling
The in-house script uses SMALT v0.7.4 (Available from: https://sourceforge.net/projects/smalt/) to map reads against a selected reference including randomly mapping the repeats and using the GATK indel alignment option. Variation detection was performed using samtools mpileup v0.1.19 and bcftools v0.1.19 to produce a BCF file of all sites and all variant sites.

For manuscripts utilizing custom algorithms or software that are central to the research but not yet described in published literature, software must be made available to editors/reviewers. We strongly encourage code deposition in a community repository (e.g. GitHub). See the Nature Research guidelines for submitting code & software for further information.

Data

Policy information about availability of data

All manuscripts must include a data availability statement. This statement should provide the following information, where applicable:

- Accession codes, unique identifiers, or web links for publicly available datasets
- A list of figures that have associated raw data
- A description of any restrictions on data availability

The datasets generated during and/or analysed during the current study are available from the corresponding author. No participant identifiable information will be disclosed.

The raw sequence reads are available under accessions ERS3381923 (sample 1 Oxford w/t), ERS3381924 (sample 2 w/t Pre-GMT), ERS3381925 (sample 3 w/t Post-GMT), ERS3381926 (sample 4 k/o Pre-GMT), and ERS3381927 (sample 5 k/o Post-GMT). Manually refined hybrid assemblies as described above are given for the wild type strain (sample 2 w/t Pre-GMT) under accession GCA_901457615 and for the knock-out strain (sample 5 k/o Post-GMT) under accession GCA_901457625.

Field-specific reporting

Please select the one below that is the best fit for your research. If you are not sure, read the appropriate sections before making your selection.

☒ Life sciences ☐ Behavioural & social sciences ☐ Ecological, evolutionary & environmental sciences

For a reference copy of the document with all sections, see nature.com/documents/nr-reporting-summary-flat.pdf

Life sciences study design

All studies must disclose on these points even when the disclosure is negative.

Sample size

The number of participants enrolled represents a convenience sample, reflecting the number of participants that could be feasibly enrolled within the time-frame and budget of the study. Assuming that the typhoid toxin is central to the clinical presentation of acute typhoid fever, it was anticipated that the attack rate following challenge with the TN strain would be reduced compared with the WT strain, although the effect size was unknown.

Assuming an attack rate of 65% following WT challenge and 50% attack rate following TN strain, and accounting for 10% drop out, 20 participants in each group gave 95% confidence intervals for attack rate of 41% to 85% in the WT group and 27% to 73% in TN group. Twenty participants per arm provided 95% power to detect a relative risk reduction in attack rate of 85% (65% with WT strain versus 10% with TN strains) and 80% power to detect a relative reduction in attack rate of 72% (65% with S. Typhi wild type strain versus 18% with S. Typhi toxin-negative) based on Fisher's Exact test with 5% alpha

Data exclusions

One participant randomized to wild-type S. Typhi declined to participate after enrolment, but prior to challenge, and was excluded from all further analyses.

Replication

Participants acted as biological replicates within study groups.
Permission for deliberate release of the genetically modified toxin-negative strain of S. Typhi was given for a limited number of participants (n=20) and precluded replication in an independent cohort.

Randomization

Participants were randomized 1:1 to challenge with either wild-type strain Salmonella Typhi (WT) or toxin-negative strain Salmonella Typhi (TN) in varying block sizes.

Anti-Vi IgG was measured at screening using a commercial ELISA kit (VaccZyme, The Binding Site Ltd, Birmingham, UK) according to the manufacturer's instructions. Randomization was stratified by anti-Vi IgG (low <7.4EU/ML or high =>7.4EU/ml. The exception was a sentinel group of two participants who were randomized 1:1 to receive the WT strain or TN knock-out strain, using a block size of two.

Randomization was performed at the pre-challenge visit (Day -7), one week prior to challenge. We generated a randomisation list in STATA version 14.2, which was implemented in the computerised randomisation software Sortition (Nuffield Department of Primary Care, Clinical Trials Unit, University of Oxford), which matched a masked allocation group to each participant.

Blinding

The study was conducted in a double-blind from the time of randomisation until participant unblinding, such that the participants, and clinical or laboratory staff undertaking follow-up procedures, were unaware of challenge agent allocation. Both WT and TN strains were prepared suspended in sodium bicarbonate and had an indistinguishable appearance (transparent, colourless liquid).

Reporting for specific materials, systems and methods

We require information from authors about some types of materials, experimental systems and methods used in many studies. Here, indicate whether each material, system or method listed is relevant to your study. If you are not sure if a list item applies to your research, read the appropriate section before selecting a response.

Materials & experimental systems

n/a	Involved in the study
☐	☒ Antibodies
☐	☒ Eukaryotic cell lines
☒	☐ Palaeontology
☐	☒ Animals and other organisms
☐	☒ Human research participants
☐	☒ Clinical data

Methods

n/a	Involved in the study
☒	☐ ChIP-seq
☒	☐ Flow cytometry
☒	☐ MRI-based neuroimaging

Antibodies

Antibodies used

Antibodies:
Manufacturer: Merck Millipore
Catalog/lot number: Goat anti-human IgG γ-Chain specific conjugated to alkaline phosphatase 401442-1ml/2689825;
Goat anti-human IgA alpha-Chain specific conjugated to alkaline phosphatase 401132/D00165538.
Used at a 1:5000 dilution.

Flurospot detection antibodies, using Mabtech kit FSP-010308-10 (batch 3).
Manufacturer: Mabtech
Anti IFNg monoclonal antibody, clone 7-B6-1-BAM, batch 1, 1/200 dilution
Anti IL17A monoclonal antibody biotinylated, clone MT504, batch 7, 1/250 dilution
Fluorophore conjugates:
anti BAM-490, batch 3, 1/200 dilution; SA-550, batch 7, 1/200 dilution; anti-WASP-641, batch 7, 1/200 dilution

Validation

Immunoaffinity purified goat polyclonal anti-human IgA and anti-human IgG antibody conjugated to alkaline phosphatase, validated by manufacturer Merck Millipore.
(Manufacturers information available at http://www.merckmillipore.com/GB/en/product/Goat-Anti-Human-IgG-Chain-Specific-Alkaline-Phosphatase-Conjugate,EMD_BIO-401442#anchor_PDS)

Antibodies used in flurospot assay validated by manufacturer Mabtech.
(Manufacturers information available at https://www.mabtech.com/products/anti-human-ifn-gamma-antibody-7-b6-1-biotinylated-3420-6)

Antibodies used in ex-vivo ELISPOT assays as outlined in https://www.ncbi.nlm.nih.gov/pubmed/18032593 and https://www.ncbi.nlm.nih.gov/pubmed/27533046

Eukaryotic cell lines

Policy information about cell lines

Cell line source(s)

Henle-407 intestinal epithelial cells from the Roy Curtiss laboratory collection.

Authentication

The cells were frequently checked for their morphological features, growth speed and functionalities, but were not

Authentication	authenticated by short tandem repeat (STR) profiling.
Mycoplasma contamination	All cell lines tested negative for mycoplasma contamination.
Commonly misidentified lines (See ICLAC register)	No commonly misidentified cell lines were used.

Animals and other organisms

Policy information about studies involving animals; ARRIVE guidelines recommended for reporting animal research

Laboratory animals	CmaH -/- bloc3 -/- mice (Figure 1).<br>Age: 7 -10 week old<br>Sex: male and females, randomly assigned to the different groups
Wild animals	The study did not involve wild animals.
Field-collected samples	The study did not involve field collected specimens.
Ethics oversight	All animal experiments were conducted according to protocols approved by Yale University's Institutional Animal Care and Use Committee.

Note that full information on the approval of the study protocol must also be provided in the manuscript.

Human research participants

Policy information about studies involving human research participants

Population characteristics

Healthy adults aged 18-60 years

Key exclusion criteria included significant medical, surgical or psychiatric history, gallbladder disease and high-risk occupations as defined by Public Health England guidelines. A full description of inclusion and exclusion criteria is provided in the attached study protocol and below.

Study eligibility
Male or female participants aged 18-60 years inclusive who were in good health (as determined by a study doctor, medical investigation and review of medical history provided by their General Practitioner) and who were able to provide written informed consent were eligible for inclusion in this study.

Inclusion Criteria
Participants must satisfy all of the following criteria to be considered eligible for the study:
• Agree to give informed consent for participation in the study.
• Aged between 18 and 60 years inclusive at time of challenge.
• In good health as determined by medical history, physical examination and clinical judgment of the study team.
• Agree (in the study team's opinion) to comply with all study requirements, including capacity to adhere to good personal hygiene and infection control precautions.
• Agree to allow his or her General Practitioner (and/or Consultant if appropriate), to be notified of participation in the study.
• Agree to allow study staff to contact his or her GP to access the participant's vaccination records and summary of medical history.
• Agree to allow Public Health England to be informed of their participation in the study.
• Agree to give his or her close contacts written information informing them of the participant's involvement in the study and offer them voluntary screening for S. Typhi carriage.
• Agree to have 24-hour contact with study staff during the four weeks post challenge and to be able to ensure that they are contactable by mobile phone for the duration of the challenge period until antibiotic completion.
• Agree to allow the study team to hold the name and 24-hour contact number of a close friend, relative or housemate who will be kept informed of the study participant's whereabouts for the duration of the challenge period (from the time of challenge until completion of antibiotic course). This person will be contacted if study staff are unable to contact the participant.
• Have internet access to allow completion of the e-diary and real-time safety monitoring.
• Agree to avoid antipyretic/anti-inflammatory treatment from the time of challenge (Day 0) until advised by a study doctor or until 14 days after challenge.
• Agree to refrain from donating blood for the duration of the study.
• Agree to provide their National Insurance/Passport number for the purposes of TOPS registration and bank account details for payment of reimbursement expenses.

Exclusion Criteria
Participants were not enrolled if any of the following applied:
• History of significant organ/system disease that could interfere with trial conduct or completion. Including, for example, but not restricted to:
o Cardiovascular disease
o Respiratory disease
o Haematological disease
o Endocrine disorders
o Renal or bladder disease, including history of renal calculi

o Biliary tract disease, including biliary colic, asymptomatic gallstones or previous cholecystectomy
o Gastro-intestinal disease including requirement for antacids, H2-receptor antagonists, proton pump inhibitors or laxatives
o Neurological disease
o Metabolic disease
o Autoimmune disease
o Psychiatric illness requiring hospitalisation or known or suspected drug and/or alcohol misuse (alcohol misuse defined as an intake exceeding 42 units per week)
o Infectious disease
• Have any known or suspected impairment of immune function, alteration of immune function, or prior immune exposure that may alter immune function to typhoid resulting from, for example:
o Congenital or acquired immunodeficiency, including IgA deficiency
o Human Immunodeficiency Virus infection or symptoms/signs suggestive of an HIV-associated condition
o Receipt of immunosuppressive therapy such as anti-cancer chemotherapy or radiation therapy within the preceding 12 months or long-term systemic corticosteroid therapy.
o Receipt of immunoglobulin or any blood product transfusion within 3 months of study start.
o History of cancer (except squamous cell or basal cell carcinoma of the skin and cervical carcinoma in situ).
• Moderate or severe depression or anxiety as classified by the Hospital Anxiety and Depression Score at screening or challenge that is deemed clinically significant by the study doctors .
• Weight less than 50kg .
• Presence of implants or prosthesis.
• Anyone taking long-term medication (e.g. analgesia, anti-inflammatories or antibiotics) that may affect symptom reporting or interpretation of the study results.
• Contraindication to fluoroquinolone or macrolide antibiotics (e.g. ciprofloxacin or azithromycin).
• Female participants who are pregnant, lactating or who are unwilling to ensure that they or their partner use effective contraception 30 days prior to challenge and until three negative stool samples have been obtained after completion of antibiotic treatment.
• Full-time, part-time or voluntary occupations involving:
o Clinical or social work with direct contact with young children (defined as those attending pre-school groups or nursery or aged under 2 years), or
o Clinical or social work with direct contact with highly susceptible patients or persons in whom typhoid infection would have particularly serious consequences (unless willing to avoid work until demonstrated not to be infected with S. Typhi in accordance with guidance from Public Health England and willing to allow study staff to inform their employer).
• Full time, part time or voluntary occupations involving:
o Commercial food handling (involving preparing or serving unwrapped foods not subjected to further heating)
• Close household contact with:
o Young children (defined as those attending pre-school groups, nursery or those aged less than 2 years)
o Individuals who are immunocompromised.
• Scheduled elective surgery or other procedures requiring general anaesthesia during the study period.
• Participants who have participated in another research study involving an investigational product that might affect risk of typhoid infection or compromise the integrity of the study within the 30 days prior to enrolment (e.g. significant volumes of blood already taken in previous study) .
• Detection of any abnormal results from screening investigations (at the clinical discretion of the study team).
• Inability to comply with any of the study requirements (at the discretion of the study staff and the participant's General Practitioner).
• Any other social, psychological or health issues which, in the opinion of the study staff, may
o put the participant or their contacts at risk because of participation in the study,
o adversely affect the interpretation of the primary endpoint data,
o impair the participant's ability to participate in the study.
• Prior vaccination with an oral typhoid vaccines (e.g. Ty21a) or other investigational typhoid vaccine (e.g. Vi-conjugate vaccine, MO1ZH09)
• Prior vaccination with a Vi-polysaccharide typhoid vaccine administered within 5 years from the time of screening.
• Prior vaccination with a Vi-polysaccharide typhoid vaccine administered more than 5 years from the time of screening AND detectable Vi-antibody titre at screening (defined as IgG ≥7.4U/ml measured using the VaccZyme Salmonella Typhi Vi IgG kit, Binding Site ® UK).
• Having been resident in an enteric fever endemic country for 6 months or more.
• Have previously been diagnosed with laboratory-confirmed typhoid or paratyphoid infection or been given a diagnosis compatible with enteric fever.
• Have participated in previous typhoid or paratyphoid challenge studies (with ingestion of challenge agent).
• Have a prolonged corrected QT interval (>450 milliseconds) on ECG screening.

Recruitment

Identification of study participants
Several strategies were employed to recruit participants, including:

-NHS database: Potential study participants will be identified via databases such the National Health Applications and Infrastructure Services (NHAIS) who hold the central NHS patient database (Open Exeter) or their equivalent.

-Poster advertising: Display of posters advertising the study throughout local hospitals and doctor's surgeries, tertiary education institutions and other public places with the permission of the owner/ proprietor.

-Direct mail-out

-E-mail communication: We will contact representatives of local tertiary education establishments and local employers and ask them to circulate posters and information, and to circulate a link to study information on the OVG website by email.

	-Oxford Vaccine Centre (OVC) database for healthy volunteers<br><br>-Media advertising: Local media, newspaper, radio, website and social media advertisement placed in locations relevant for the target age group with brief details of the study and contact details for further information<br><br>-Website advertising: Description of the study and copy of information booklet on the Oxford Vaccine Group website.<br><br>Exhibitions: Advertising material and/or persons providing information relating to the study will exhibit using stalls or stands at exhibitions and/or fairs, such as University Fairs<br><br>Royal Mail Leaflet: Royal Mail door-to-door service with delivery of invitation letters in OVG envelopes to every household within certain postcode areas.<br><br>Potential participants who were interested in study participation contacted the study site by telephone, email, by out website online registration with self-screening questions or paper reply slip for further information. Once an expression of interest was received, an information booklet was be sent via mail or email to the potential participants to read at their leisure. Participants were also be directed to the Oxford Vaccine Group website, where the information booklet was available. If participants were willing to proceed they were invited for a screening and consent visit, where a member of the clinical research team at the Oxford Vaccine Group assessed their eligibility. We also took consent for clinical staff to access electronic patient records (EPR) to assess eligibility.<br>We acknowledge the potential self-selection bias in such human-challenge studies. We contend that t any potential biases are mitigated by standardized procedures and consistent inclusion/exclusion criteria. These limitations are discussed within the manuscript.
Ethics oversight	The OVG2016/03 study was sponsored by the University of Oxford (Clinical Trials & Research Governance). Ethical approvals for the primary protocol, and any study amendments, were obtained from the South-Central Oxford A research ethics committee (16/SC/0358). In the UK, legislation governing the deliberate release of genetically modified organisms is currently provided by the Environmental Protection Act 1990 section 111 and 11242, and the Genetically Modified Organisms (Deliberate Release) Regulations 200243. Approvals for deliberate release of the genetically modified strain of S. Typhi were obtained from the United Kingdom Department for Environment, Food & Rural Affairs (16/R48/01)44

Note that full information on the approval of the study protocol must also be provided in the manuscript.

Clinical data

Policy information about clinical studies

All manuscripts should comply with the ICMJE guidelines for publication of clinical research and a completed CONSORT checklist must be included with all submissions.

Clinical trial registration	Clinicaltrials.gov NCT03067961
Study protocol	Submitted alongside manuscript
Data collection	Centre for Clinical Vaccinology & Tropical Medicine, The Churchill Hospital, Oxford, United Kingdom<br>OX3 7LE<br>10th April 2017 and 1st August 2017
Outcomes	Primary Outcome Measure<br><br>The primary outcome was the attack rate post challenge, defined as the proportion of participants meeting the composite diagnostic endpoint for typhoid fever during the challenge period.<br>The composite diagnostic endpoint for typhoid fever was defined as a temperature of 38C persisting for >12hrs and/or S. Typhi bacteraemia collected >72hours after oral challenge<br><br>Secondary clinical and microbiological outcome variables are listed by below<br><br>• Time to diagnosis - Time from date/time of challenge to date/time of first temperature >38C that subsequently lasted for >12hours OR the date/time of first positive blood culture collection (whichever occurs earliest).<br>• Time to first blood culture positive for S. Typhi- Time from date/time of challenge to date/time of blood culture collection.<br>• Time to clinical diagnosis (fever >38C lasting >12 hours) - Time from date/time of challenge to date/time of first recorded temperature >38C which subsequently lasted 12 hours.<br>• Mode of diagnosis - The proportion of participants diagnosed with typhoid/paratyphoid fever based upon either clinical criteria (persistent fever >38C for >12 hours) OR microbiological criteria (blood culture positive for S. Typhi collected >72 hours from diagnosis)<br>• Detailed mode of diagnosis - Proportion of participants diagnosed with typhoid/paratyphoid fever based upon either of the following specific diagnostic criteria:<br>Temperature >38C preceding positive blood culture;<br>o Temperature >38C without positive blood culture;<br>o S. Typhi bacteraemia preceding temperature >38C;<br>o S. Typhi bacteraemia without temperature >38C.<br>• Time to first fever - Time from date/time of challenge to date/time of first recorded temperature >38C.<br>• Fever clearance time - Time from initiation of antibiotics or start of fever (whichever was later) to first recorded temperature <38oC persisting for at least 48hours. Only diagnosed participants with fever were included in the analysis.

• Symptom severity - Severity of symptoms in each challenge group were assessed by:
o The proportion of participants with maximum symptom severity score graded as mild, moderate or severe following challenge.
o The proportion of participants meeting the criteria for severe enteric fever.
o Individual enteric fever severity scores calculated by summing numerical values assigned to the severity of individual solicited symptoms, clinical observations (heart rate, systolic blood pressure, diastolic blood pressure and temperature) and laboratory measurements between Day 0 to Day 21 (0=not present; 1=mild; 2=moderate; 3=severe; 4 = Hospitalisation.
o Duration of bacteraemia - Time (Hours/Days) from collection of first positive blood culture until date/time of the last positive blood culture.
o Bacteraemia clearance time - Time (Hours/Days) from collection of first positive blood culture until date/time of the first negative blood culture remaining persistently negative. Participants with missing data (e.g. no negative blood cultures after commencing antibiotics) were censored in the analysis at the time point of the last culture taken.
o Stool shedding - Daily stool culture(s) positive for S. Typhi for 14 days post-challenge.
• Quantitative blood culture - Concentration of bacteria in 10ml blood taken at the time of diagnosis using the Wampole™ Isostat® Isolator system (Colony forming units/ml). For values below the lower limit of detection (0.1 CFU/ml), a value of 0.05 CFU/ml was assigned.
• Haematological and biochemical end-points - The following haematological parameters were measured from time of challenge to Day 28 and/or Day 90.
o Total Haemoglobin (g/L)
o Haemoglobin change from baseline (Hb g/l D0 – Hb g/l D14)
o Total White Cell Count (x109/l)
o Platelet counts (x109/l)
o Neutrophil count (x109/l)
o Lymphocyte count (x109/l)
o Monocyte count (x109/l)
o Eosinophil count (x109/l)
o Monocyte/Lymphocyte ratio
o Urea & Electrolytes (Na, K+, Urea, Creatinine –mmol/l)
o C-reactive protein (mg/l)
o Liver function tests (Bilirubin [umol/l], aspartate transaminase (AST IU/l), alkaline phosphatase (ALP IU/l), alanine transaminase (ALT IU/l), Albumin (g/L)
• Safety outcome measures - Adverse events, adverse events of special interest, SAE's and SUSARs according to each study group.
