# Supplementary material for: Investigation of the role of typhoid toxin in acute typhoid fever in a human challenge model
Source: Nat Med. 2019 Jul 3;25(7):1082–8. doi: 10.1038/s41591-019-0505-4 (PMC6892374; doi:10.1038/s41591-019-0505-4)
Supplement: Supplementary file 4 — Study protocol [file 41591_2019_505_MOESM4_ESM.pdf]

Investigating the role of typhoid toxin in the pathogenesis of enteric fever: A double-blinded, randomised, outpatient human challenge study.

Internal Reference Number / Short title: 2016/03: Investigating Typhoid Fever Pathogenesis (TYGER)

Ethics Ref: 16/SC/0358

Date and Version No: 16-Feb-2018, Version 4.0

**Chief Investigator**	Professor Andrew J Pollard
**Co-Investigators**	Professor Brian Angus
**Study Site**	Centre for Clinical Vaccinology & Tropical Medicine (CCVTM), Churchill Hospital, Oxford OX3 7LE, United Kingdom
**Study Code**	2016/03
**Sponsor**	University of Oxford, University Offices, Wellington Square, Oxford, OX1 2JD
**Clinicaltrials.gov number**	NCT03067961
**Funding Support**	Bill and Melinda Gates Foundation OPP1126235

Investigator Agreement

"I have read this protocol and agree to abide by all provisions set forth therein. I agree to comply with the International Conference on Harmonisation Tripartite Guideline on Good Clinical Practice."

Signatures:

Date: 16-Feb-2018

Prof Andrew Pollard
Chief Investigator, Oxford Vaccine Group, Department of Paediatrics, University of Oxford

Date: 16-Feb-2018

Prof Brian Angus
Principal Investigator, Nuffield Department of Medicine, University of Oxford

Confidentiality Statement

This document contains confidential information that must not be disclosed to anyone other than the Sponsor, the Investigator Team, host organisation, regulation authorities, and members of the Research Ethics Committee, unless authorised to do so.

CONTENTS

CONTENTS 3
1. CONTACTS 7
2. SYNOPSIS 8
3. ABBREVIATIONS 11
4. BACKGROUND 15
4.1. Pathogenesis of *Salmonella* Infection 16
4.2. Typhoid Toxin 17
4.3. Typhoid and Paratyphoid challenge studies 19
4.4. The Quailes strain 20
4.5. Aim of the project 21
5. OBJECTIVES AND ENDPOINTS 23
6. STUDY DESIGN 25
6.1. Summary of the study design 25
6.2. Visit structure 25
7. STUDY PARTICIPANTS 32
7.1. Study eligibility 32
7.2. Inclusion Criteria 32
7.3. Exclusion Criteria 33
7.4. Pregnancy and contraception 36
7.5. Trial Over-volunteering Prevention System 36
7.6. Potential benefits to participants 37
7.7. Potential risks to participants 37
7.8. Potential risks to close contacts of participants 39
8. TRIAL PROCEDURES 41
8.1. Recruitment 41
8.2. Informed Consent 42
8.3. Screening and Eligibility Assessment 43
8.4. Definition of enrolment 45
8.5. Randomisation, blinding and code-breaking 46
8.6. *S.* Typhi Challenge Procedure 47
8.7. Post-challenge visits; non-typhoid diagnosis visits (D0+12 to D365) 49
8.8. Outside of scheduled visits and unscheduled visits 49
8.9. Typhoid diagnosis and typhoid diagnosis visits (TD to TD+96 hours) 50

8.10. Reporting to the Health Protection Unit ... 52
8.11. Concomitant medication for symptoms of typhoid infection ... 53
8.12. Antibiotic treatment ... 54
8.13. Stool culture for carriage of *S.* Typhi ... 55
8.14. Screening of close contacts for carriage of *S.* Typhi ... 55
8.15. Transport of samples ... 56
8.16. Discontinuation/ Withdrawal of trial participants ... 56
8.17. Participant questionnaire ... 56
8.18. Definition of End of Trial ... 57
9. INTERVENTIONS ... 58
9.1. *S.* Typhi challenge strain ... 58
9.2. Compliance with Trial Treatment ... 59
9.3. Concomitant Medication ... 59
9.4. Post-trial Treatment ... 59
10. LABORATORY ... 60
10.1. Blinding of laboratory samples ... 60
10.2. Bacteriology ... 60
10.3. Molecular diagnostics ... 62
10.4. Inflammatory responses ... 62
10.5. Antibody responses ... 62
10.6. Cellular immune responses ... 63
10.7. Factors affecting susceptibility and response to infection ... 63
10.8. Other laboratory investigations ... 64
11. SAFETY REPORTING ... 65
11.1. Definitions (AE) ... 65
11.2. Definition of an Expected Adverse Event ... 66
11.3. Causality ... 67
11.4. Adverse Events of Special Interest (AESI) ... 68
11.5. Expected Serious Adverse Events and Serious Adverse Reactions ... 69
11.6. Procedures for recording Adverse Events ... 69
11.7. Procedures for reporting Serious Adverse Events ... 70
11.8. SUSAR Reporting ... 71
11.9. Annual Progress report (APR) ... 71
11.10. Safety Profile Review ... 71

11.11. Trial Management Committee 71
11.12. Data Safety Monitoring Committee 71
11.13. Procedure to be followed in the event of an abnormal finding 72
11.14. Staff and Investigator safety 72
12. STATISTICS 73
12.1. Description of Statistical Methods 73
12.2. The Number of Participants 73
12.3. Analysis of demographic and baseline characteristics 74
12.4. Analysis of the primary endpoint 74
12.5. The Level of Statistical Significance 75
12.6. Criteria for the Termination of the Trial 76
12.7. Procedure for Accounting for Missing, Unused, and Spurious Data. 76
12.8. Inclusion in Analysis 76
12.9. Procedures for Reporting any Deviation(s) from the Original Statistical Plan 77
13. DATA MANAGEMENT 78
13.1. Access to Data 78
13.2. Source Data 78
13.3. Data Recording and Record Keeping 78
13.4. Data integrity 79
13.5. Data archiving and storage 79
14. QUALITY ASSURANCE PROCEDURES 80
14.1. Investigator procedures 80
14.2. Monitoring 80
14.3. Direct access to source data/documents 80
14.4. Modification to protocol 80
14.5. Protocol deviation 81
14.6. Audit and Inspection 81
14.7. Trial Progress 81
15. ETHICAL AND REGULATORY CONSIDERATIONS 82
15.1. Declaration of Helsinki 82
15.2. Guidelines for Good Clinical Practice 82
15.3. Approvals 82
15.4. Reporting 82
15.5. Participant confidentiality 82

15.6. Participant reimbursement 82
15.7. Other Ethical Considerations 83
16. FINANCE AND INSURANCE 84
16.1. Funding 84
16.2. Insurance 84
17. PUBLICATION POLICY 85
18. REFERENCES 86
19. APPENDIX A: Grading the severity of solicited and unsolicited systemic Adverse Events 89
20. APPENDIX B: Grading the severity of visit observed Adverse Events 90
21. APPENDIX C: Grading the severity of laboratory Adverse Events 91
22. APPENDIX D: AMENDMENT HISTORY 92

1. CONTACTS

**Chief Investigator**	Professor Andrew J Pollard<br>Professor of Paediatrics and Immunity<br>Oxford Vaccine Group, University of Oxford,<br>Centre for Clinical Vaccinology and Tropical Medicine (CCVTM),<br>Churchill Hospital, Oxford, OX3 7LE, United Kingdom<br>andrew.pollard@paediatrics.ox.ac.uk
**Co-Investigator**	Professor Brian Angus<br>Professor of Infectious Diseases / Consultant Physician /<br>Director Oxford Centre for Clinical Tropical Medicine,<br>University of Oxford,<br>Nuffield Department of Medicine Research Building (NDRMB),<br>Old Road Campus, Headington, Oxford, OX3 7FZ, United Kingdom.<br>brian.angus@ndm.ox.ac.uk
**Sponsor**	The University of Oxford
**Monitor**	Clinical Trials and Research Governance<br>Joint Research Office, Block 60,<br>Churchill Hospital<br>Old Road, Headington<br>Oxford OX3 7LE<br>United Kingdom
**Statistician**	Ly-Mee Yu<br>Trial Statistician<br>Primary Care Clinical Trials Unit,<br>Nuffield Department of Primary Care Health Sciences<br>University of Oxford<br>Gibson Building, Radcliffe Observatory Quarter, Woodstock Road<br>Oxford, OX2 6GG<br>Tel: 01865 289288<br>Email:ly-mee.yu@phc.ox.ac.uk
**DSMC Chair**	Dr. David Lalloo

2. SYNOPSIS

**Study Title**	Investigating the role of typhoid toxin in the pathogenesis of enteric fever: A double-blinded, randomised, outpatient human challenge study.	
**Short title (internal ref.)**	OVG 2016/03: Investigating Typhoid Fever Pathogenesis (TYGER)	
**Study Design**	Double-blinded, randomised, outpatient, ambulatory, human infection study	
**Study Participants**	Healthy adults aged 18 to 60 years inclusive	
**Planned Sample Size**	A total of 40 participants will be randomised 1:1 to receive challenge with either wild type *Salmonella* Typhi Quailes strain (WT) or a typhoid toxin-deficient isogenic mutant of *S.* Typhi Quailes strain SB6000 (TN). Assuming an attack rate of 65% following WT strain and 50% attack rate following TN strain, and accounting for 10% drop out, 20 participants in each group will lead to 95% confidence intervals for attack rate of 40% to 84% in the group randomised to WT strain and 27% to 72% in the group randomised to TN strain.	
**Follow up duration**	12 months	
**Planned Trial Period**	Clinical phase: 1st September 2016 – 31st August 2017	
**Objectives and Endpoints**		
	**Objective(s)**	**Endpoint(s)**
**Primary**	To investigate the contribution of typhoid toxin to *S.* Typhi infection and typhoid fever using a human challenge model of infection.	The proportion of participants developing clinical or microbiologically proven typhoid infection following oral challenge with 1-5x10^4 CFU wild type *S.* Typhi Quailes strain (WT), in comparison with 1-5x10^4 CFU of a typhoid toxin-deficient isogenic mutant of *S.* Typhi Quailes strain SB6000 (TN).
**Secondary**	To compare clinical and laboratory features following challenge with either wild-type *S.* Typhi Quailes strain (WT) or typhoid toxin-deficient isogenic mutant *S.* Typhi Quailes strain SB6000 (TN).	Comparison of the clinical course of typhoid infection after challenge, between wild-type (WT) and typhoid toxin-deficient mutant (TN) *S.* Typhi Quailes strain SB6000, with particular reference to :<br>• time to onset of symptoms<br>• duration of illness including fever clearance time<br>• symptom severity<br>• time to diagnosis<br>• time to onset of bacteraemia<br>• duration of bacteraemia<br>• quantification of bacteraemia at time of diagnosis

		• time to onset and pattern of stool shedding<br>• inflammatory response<br>• haematological and biochemical parameters<br><br>using clinical reporting, physical examination findings, microbiological to detect *S.* Typhi in blood and stool, and laboratory assays to monitor inflammatory responses.
	To compare the host immune response following challenge with either wild-type *S.* Typhi Quailes strain (WT) or typhoid toxin-deficient isogenic mutant *S.* Typhi Quailes strain SB6000 (TN).	Comparison of the host innate, humoral and cell-mediated responses to challenge with WT and TN strains at baseline (Day 0) and post-challenge time points, with particular reference to, but not limited to,:<br>• *S.* Typhi specific antibody concentrations;<br>• Frequency and magnitude of *S.* Typhi specific antibody-secreting cells as measured by ex-vivo and memory B-cell ELISPOT;<br>• Description of lymphocyte populations at baseline and following challenge as measured by flow cytometry and/or CyTOF;<br>• Frequency and magnitude of *S.* Typhi specific cell-mediated immune responses as measured by ELISPOT and flow cytometry and/or CyTOF;<br>• Plasma Cytokine concentration
	To describe the variation in gene expression in whole blood following challenge with either wild-type *S.* Typhi Quailes strain (WT) or typhoid toxin-deficient isogenic mutant *S.* Typhi Quailes strain SB6000 (TN).	Comparison of differentially expressed genes using RNASeq on whole blood and/or PBMC samples at baseline (Day 0) and post-challenge time points following challenge with WT and TN strains.

	To investigate indirect effects of typhoid-toxin *in vivo.*	Analysis comparing blood and/or urine samples from participants challenged with WT and TN strains, using proteomic and metabolomics based techniques at baseline (Day 0) and post-challenge time points.
	To investigate methods for direct detection of typhoid toxin *in vivo* following challenge with wild-type *S.* Typhi Quailes strain (WT).	Exploratory analysis of blood and/or urine and/or faecal samples including use of assays including (but not limited to) mass-spectrometry.
Exploratory Objectives	To investigate how the human microbiota, including nasal carriage of *Staphylococcus aureus*, influences and interacts with a challenge of *S*. Typhi.	Samples of nasal swabs, saliva and stool to measure the constituent microbiological flora by culture and metagenomic studies.
	To investigate new molecular techniques for detection of *S*. Typhi in clinical samples.	Use of novel methodologies to prepare bacterial DNA/RNA and development of sensitive quantitative and qualitative PCR assays for blood, urine and stool specimens.
	To investigate recruitment methods and reasons for volunteer exclusion from typhoid challenge models.	Analysis of recruitment numbers, including:<br>• Number of positive and negative responses to different recruitment techniques;<br>• Number of volunteers excluded prior to attending screening visits and reasons for exclusion;<br>• Number of volunteers attending for screening visits and reasons for exclusion.

**Challenge Agents**		
1)	**Dose/formulation:**<br>Wild-type *Salmonella enterica* serovar Typhi Quailes strain (WT)	$1\text{-}5\times10^4$ CFU suspended in sodium bicarbonate prior to oral ingestion

2)	Typhoid toxin-deficient mutant *Salmonella enterica* serovar Typhi Quailes strain SB6000 (TN)	1-5x10^4 CFU suspended in sodium bicarbonate prior to oral ingestion

3. ABBREVIATIONS

AE	Adverse event
AESI	Adverse event of special interest
ALP	Alkaline phosphatase
ALT	Alanine transaminase
AR	Adverse reaction or Attack Rate
ASC	Antibody secreting cell
AST	Aspartate transaminase
BD	bis in die (Latin: twice a day; prescription medicines)
BMGF	Bill and Melinda Gates Foundation
BMI	Body Mass Index
CI	Chief investigator or confidence interval
CCVTM	Clinical Centre for Vaccinology and Tropical Medicine
cdtB/CdtB	Cytolethal Distending Toxin sub-unit B
CFU	Colony forming unit
CMI	Cell-mediated immunity
CRF	Case Report Form
CRO	Contract Research Organisation
CRP	C-reactive protein
CT	Clinical Trials
CTA	Clinical Trials Authorisation
CTL	Cytotoxic T-lymphocyte
CTRG	Clinical Trials & Research Governance, University of Oxford
DSMC	Data Safety and Monitoring Committee
DSUR	Development Safety Update Reports
EDTA	Ethylenediamine tetraacetic acid
ELISA	Enzyme linked immunosorbent assay
ELISPOT	Enzyme linked immunosorbent spot assay
EPI	Expanded Program on Immunisation

ESR	Erythrocyte sedimentation rate
GCP	Good Clinical Practice
GMT	Geometric Mean Titre
GP	General Practitioner
HADS	Hospital Anxiety and Depression Scale
HIV	Human Immunodeficiency Virus
(TV) HPU	(Thames Valley) Health Protection Unit
IB	Investigators Brochure
ICF	Informed Consent Form
ICH	International Conference of Harmonisation
IFN	Interferon
IgA	Immunoglobulin A
IgM	Immunoglobulin M
IgG	Immunoglobulin G
IL	Interleukin
IMP	Investigational medicinal product
IRB	Independent Review Board
ISF	Investigator site file
LFT	Liver function tests
LLN	Lower limit of normal
LPS	Lipopolysaccharide
MAX	Maximum (prescription medication)
MHRA	Medicines and Healthcare products Regulatory Agency
NIH	National Institute of Health
NRES	National Research Ethics Service
OUH	Oxford University Hospital NHS Trust
OVC	Oxford Vaccine Centre
OVG	Oxford Vaccine Group
OVGL	Oxford Vaccine Group Laboratory
PBMC	Peripheral blood mononuclear cell
PCR	Polymerase chain reaction
PE	Protective Effect
PHE	Public Health England
PIL	Participant/ Patient information leaflet

pltA/PltA	Pertussis like toxin A
pltB/PltB	Pertussis like toxin B
PO	Per oral (by mouth)
PR	Per rectum (by rectum)
PRN	pro re nata (latin: as required; prescription medicines)
QDS	quater die sumendus (Latin: four times a day; prescription medicines)
R&D	NHS Trust Research & Development Department
REC	Research Ethics Committee
REC ref.	Research Ethics Committee reference
RPM	Revolutions per minute
RRT	Renal replacement therapy
SAE	Serious adverse event
SAR	Serious adverse reaction
SBA	Serum bactericidal assay
SCV	*Salmonella* Containing Vacuole
SMP(C)	Summary of Medicinal Product (Characteristics)
SOP	Standard Operating Procedure
SPI	Salmonella pathogenicity island
SUSAR	Suspected unexpected serious adverse reactions
T3SS	Type 3 Secretion System
TCV	Tetanus (toxoid) Conjugate Vaccine
TD	Typhoid Diagnosis
TDS	ter die sumendum (Latin: three times a day; prescription medicines)
TMF	Trial Master File
TN	Typhoid Toxin-deficient isogenic mutant of *Salmonella enterica* subsp. *enterica* serovar. Typhi Quailes strain (SB6000)
TOPS	The Over volunteering Prevention System (see: http://www.tops.org.uk)
TSB	Tryptone soya broth
TT	Typhoid Toxin
TSC	Trial Steering Committee
TSG	Oxford University Hospitals Trust / University of Oxford Trials Safety Group
ULN	Upper limit of normal
V[n]	Visit [number]
Vac	Vacutainer

Vi	Virulence antigen
WBC	White blood cell/count
WT	Wild-Type *Salmonella enterica* subsp. *enterica* serovar. Typhi Quailes strain
XLD	Xylose lysine deoxycholate
95%CI	95% Confidence Interval

4. BACKGROUND

Enteric fever is a systemic illness caused by infection with human-restricted pathogens *Salmonella enterica* subspecies *enterica* serovar Typhi (*S.* Typhi) and *Salmonella enterica* subspecies *enterica* serovar Paratyphi (*S.* Paratyphi). It continues to pose a significant burden of disease with an estimated 25 million cases and 200,000 fatalities occurring globally in the year 2000. The highest burden of disease is found in low-income countries, where poor sanitation results in the contamination of water and food products and the spread of disease [1]. Most parts of South Asia, South-East Asia, Central Asia, Africa, and South America are considered endemic, with an annual incidence of >100 per 100,000 population [2]. Although conventionally thought to be a disease of school-aged children and young adults, there is increasing evidence for high rates of infection in children fewer than 5 years of age in endemic areas [3 4]. Enteric fever also remains an important health consideration in developed countries for travellers visiting endemic regions and laboratory workers [5–7].

Humans are the only reservoir of typhoid infection, with the gallbladder thought to be the main site for long-term carriage[8,9]. Spread between individuals occurs through faecal-oral transmission, mainly via contaminated food or water. In areas with adequate sanitation (i.e. flushing latrines), person-to-person transmission is rare [10]. A retrospective case note review studied 645 contacts of 329 cases of enteric fever in London [11]. All had travelled to endemic regions, and only 10 additional cases were identified, all of whom had travelled abroad with their contact and were presumably infected by the same source rather than from person-to-person contact.

The treatment of typhoid fever has been complicated by the appearance and global dissemination of S. Typhi strains with resistance to all first-line antimicrobials [12] . Use of ineffective antibiotics has been associated with adverse clinical outcomes in patients with typhoid fever [13] and antibiotic treatment in endemic countries is often empiric and provided by untrained practitioners. The lack of sensitive and specific diagnostic tests further hampers clinical decision-making. Antibiotic susceptibility testing requires culture of the causative organism and adequate microbiology laboratory services which may not be available in many resource-limited settings. Even in advanced healthcare settings, the interpretation of antimicrobial susceptibility profiles is complex and frequently changing [14]. Although the optimal treatment regimen for enteric fever is still under investigation [15], fluoroquinolones (for example, ciprofloxacin) and azithromycin are considered the suitable oral treatment options for susceptible strains and for clearance of long-term carriage [16 17].

Efforts to eradicate enteric fever will rely on improvements in sanitation and infrastructure to prevent on-going transmission of infection. However, given the difficulties and costs involved in implementing such changes in resource-limited countries, vaccination of populations at the highest risk of developing enteric fever is likely to have the greatest impact on disease prevalence. The development of new vaccines and therapeutics for enteric fever can be aided by understanding the pathogenic mechanisms underlying the disease and by

studying the human immune response during infection. However, the pathogenesis of *S.* Typhi and *S.* Paratyphi is only partially understood and there remains no readily reproducible immunological correlate of protection. These limitations stem in part from the lack of a relevant small-animal model in which the disease can be studied, as both *S.* Typhi and *S.* Paratyphi are human-restricted pathogens.

In order to address these problems, the Oxford Vaccine Group (OVG) has established and successfully implemented an outpatient, ambulant, *S.* Typhi human challenge model in healthy adult volunteers [18]. The design of these studies was based in part upon a series of typhoid controlled human infection studies that were undertaken at the University Of Maryland School Of Medicine between 1952 and 1974. To date, this model has provided a unique opportunity to investigate a human-restricted pathogen in a biologically-relevant model and within the context of a strictly monitored experimental setting. Additionally, this challenge model has been used to study the efficacy of novel typhoid vaccines and to aid the development of new typhoid diagnostics.

In this protocol, the human challenge model will be used to further our understanding of the pathogenesis of typhoid fever by exploring the contribution of a recently identified virulence factor, termed the typhoid-toxin, to the clinical syndrome of enteric fever. It is hoped that the insights obtained from this study will form the basis for future studies exploring the use of typhoid-toxin as a novel vaccine candidate as well as providing insights into disease pathogenesis that could facilitate the development of novel diagnostic strategies and therapeutics.

4.1. Classification and Pathogenesis of *Salmonella enterica*

Salmonellae are facultative anaerobic gram-negative organism that can cause a diverse range of infections. There are over 2000 *Salmonella* serovars, classified according to their major surface antigens: O (Lipopolysaccharide); H (flagellum) and Vi-polysaccharide. Although there are several different types of *Salmonella enterica,* the species can be broadly divided into two main groups, commonly referred to as typhoidal or non-typhoidal *Salmonella* (NTS). This broad distinction is of clinical and epidemiological significance, as typhoidal and non-typhoidal *Salmonella* cause distinct clinical syndromes and also differ in terms of their host range.

The typhoidal *Salmonella* serovars comprise *S.* Typhi and *S.* Paratyphi. Both *S.* Typhi and *S.* Paratyphi are human-restricted pathogens that characteristically cause an invasive disease with bacteraemia and prominent systemic symptoms, such as fever, headache and malaise[19]. S. Typhi and *S.* Paratyphi cause an indistinguishable clinical syndrome from one another[20]. In contrast, non-typhoidal *Salmonella* comprise several hundred different serovars that typically possess a broad host range. Unlike the typhoidal *Salmonella*,

NTS serovars classically cause a localised gastrointestinal infection, characterised by diarrhoea and abdominal pain, although invasive disease can occur.

The molecular basis for the host specificity and distinct clinical phenotype of the typhoidal *Salmonella* serovars is likely to be multifactorial [21]. Whole genome sequencing of *S.* Typhi and *S.* Paratyphi reveals the presence of multiple deletions, frame shift mutations and stop codon insertions, leading to the presence of >200 'pseudogenes'. Several of these pseudogenes correspond to genes known to contribute to virulence and in non-typhoidal *Salmonella* and may, in part, account for the restricted host range and unique phenotype of *S.* Typhi and *S.* Paratyphi [22–24].

Following ingestion of bacteria in contaminated food or water, the bacteria must first pass through the gastric acid environment of the stomach. The bacteria subsequently invade intestinal mucosa via attachment to the M (microfold) cells of Peyer's patches. It is thought that professional phagocytic cells ingest the bacteria within the lamina propria of the intestine. Subsequent dissemination of bacteria contained within macrophages is thought to cause a subclinical primary bacteraemia. This results in dissemination of *S.* Typhi to the liver, spleen, lymph nodes and bone marrow. After a latent incubation period of approximately one week, patients develop symptomatic secondary bacteraemia. Delayed or inadequate treatment can result in the development of complications including gastrointestinal perforation or haemorrhage due to hyperplasia, ulceration and necrosis of Peyer's patches [25].

Much of what is known about the pathogenesis of typhoidal *Salmonella* has been inferred from studies using non-typhoidal *Salmonella enterica* serovars that can infect small animals, such as *Salmonella* Typhimurium. Administration of the *Salmonella* Typhimurium to mice recapitulates several of the key features of enteric fever and whilst acting a valuable animal model of infection, a number of key differences between *Salmonella* Typhimurium and the typhoidal serovars Typhi and Paratyphi limit the generalizability of these findings to the human model. The human challenge model has the potential overcome some of these limitations.

4.2. Typhoid Toxin

It is hypothesised that differences between typhoidal and non-typhoidal *Salmonella* may in part be explained by the acquisition of new virulence factors. Comparative genomic studies have identified approximately 601 genes unique to *S*. Typhi compared with *S*. Typhimurium, and 479 genes that are unique to *S*. Typhimurium compared to *S*. Typhi [22], indicating that there are a relatively small number of genes that are unique to typhoidal *Salmonella* serovars. One virulence factor that is limited to *S.* Typhi and *S*. Paratyphi is a recently described exotoxin termed the **typhoid toxin**. There is a growing body of evidence, which indicates that the typhoid toxin may play a key role in the pathogenesis of enteric fever.

The components of the typhoid toxin are encoded on a pathogenicity island comprising five genes– *cdtB*, *pltA*, *pltB*, *ttsA* and sty1887[22]. The *cdtB* gene encodes a homologue of the *Campylobacter* toxin subunit CdtB, whilst *pltA* and *pltB* encode homologues of the *Bordetella Pertussis* toxin sub-units. This pathogenicity island is found in a limited number of non-Typhoidal *Salmonella* serovars, but not in *Salmonella* Enteritidis or *Salmonella* Typhimurium, which are responsible for largest burden of invasive NTS disease in Africa [26 27].

The typhoid toxin comprises enzymatic (A) and binding (B) components and is composed of three polypeptide subunits (CdtB, PltA and PltB). The enzymatic component of the toxin is Cytolethal Distending Toxin B (CdtB), a homologue of active A subunit of Cytolethal distending toxin. This toxin is present in several bacterial pathogens and possesses DNAse activity capable of inducing cell-cycle arrest and cellular distension *in vitro* [28]. CdtB is covalently linked to Pertussis-like toxin A (PltA), a homologue of the enzymatic A subunit of pertussis toxin, which has ADP-ribosylase activity. PltA in turn is linked to Pertussis-like toxin B, a homologue of one of the binding (B) sub-units of pertussis toxin [29]. The crystal structure of the typhoid toxin has been resolved and shows the sub-units to be arranged into a relatively unique A_2B_5 structure, comprising two enzymatic A components (CdtB, PltA) and a homopentameric binding B portion (PltB) [29].

CdtB is thought to account for the majority of the toxin's enzymatic activity, but all three components are required for toxin delivery and toxicity[30]. The typhoid toxin is expressed by the bacterium from the intracellular compartment of macrophages within the Salmonella Containing Vacuole (SCV), from where is trafficked to the extracellular space to target cells in an autocrine and paracrine fashion [30].

Administration of purified typhoid toxin in mice recapitulates several of the key features of enteric fever, including lethargy, weight loss, leukopaenia, neutropaenia and death[29,28]. Interestingly, typhoid toxin administration does not induce fever in the mouse model, however, it is unknown if typhoid toxin produces fever in humans, as its role in the pathogenesis of disease in humans has not previously been studied [29].

Recent evidence suggests that the unique binding properties of typhoid toxin may, in part, account for the host restriction properties of typhoidal *Salmonella*. The typhoid toxin binds to a broad range of human cells via the PltB sub-unit. Binding is mediated by an interaction with a range of cell-surface glycoproteins, which terminate in specific sialoglycans - N-Acetylneuraminic Acid (Neu5AC). In contrast to humans, sialoglycans on the surface of cells from other old-world primates and most mammals possess a different configuration, terminating in N-Glycolylneuraminic Acid (Neu5GC). This is because humans lack a specific enzyme (CMP-N-acetylneuraminic acid hydroxylase (CMAH)) required to convert Neu5AC to Neu5GC. Typhoid toxin preferentially binds, and is toxic to, cells expressing the human signature Neu5AC, as in comparison to cells expressing Neu5GC, as most other mammals [31].

Importantly, typhoid toxin is highly immunogenic in humans. IgG antibodies against CdtB have been identified in the serum of typhoid fever patients from Vietnam, the presence of which was used to discriminate healthy controls and from patients infected with Non-Typhoidal Salmonella[32]. Similar findings have been found using Antibody-in-Lymphocyte supernatant (ALS) samples from typhoid fever patients in Bangladesh[33].

Converging lines of evidence suggest that typhoid toxin plays a central role in the pathogenesis of typhoid fever, which may account for the human-restriction properties of this pathogen as well as being a strongly immunogenic antigen following natural infection. These data raise the intriguing possibility that typhoid toxin could be a promising vaccine candidate for typhoidal *Salmonella.* The lack of a meaningful animal model has, however, hampered the ability to study the potential importance of typhoid toxin in the pathogenesis of typhoid fever.

4.3. Typhoid and Paratyphoid challenge studies

A human challenge model of typhoid infection was first developed at the University of Maryland in the 1950s [34,35]. Studies performed involved almost 2,000 participants and described the development of humoral immunity after inoculation with *S*. Typhi in vaccinated and unvaccinated volunteers [36]. Studies in Maryland were terminated due to concern regarding the use of prisoners and other vulnerable groups in clinical research; the prison environment was considered overly coercive, despite the ethically progressive nature in which the Maryland studies were performed.

A human typhoid challenge model was developed at the University of Oxford by the Oxford Vaccine Group between 2009 and 2012 (OVG 2009/10 Oxford A REC ref. 10/H0604/53, OVG 2011/02 Oxford A REC ref. 11/SC/0302). This new model used sodium bicarbonate as a gastric acid neutralising buffer, allowing a lower challenge dose to be used and resulting in a more consistent pattern of clinical infection. The model was also set up to be performed using outpatient community volunteers, and was demonstrated to be safe and acceptable to participants [37].

In Oxford, 41 healthy volunteers were challenged with escalating dose levels of *S.* Typhi delivered in a sodium bicarbonate buffer solution from 2009 to 2010. Participants were then followed daily until diagnosed with typhoid infection or day 14 at which point they were treated with eradicative antibiotics. Participants developing infection tolerated the clinical course well, and all participant symptoms responded to antibiotic treatment. Four participants met predetermined criteria for severe infection (2 recorded temperatures exceeding 40°C and had >grade 3 laboratory abnormalities), however, no participants required hospital admission, intravenous antibiotics or fluids. Participants were promptly treated on confirmation of typhoid diagnosis and no instances of stool shedding after treatment or transmission to secondary contacts were detected [37].

This model was then used in a subsequent clinical trial (OVG 2011/02 Oxford A REC ref. 11/SC/0302) to assess the protective efficacy of a novel, single dose oral typhoid vaccine (M01ZH09). The typhoid model was validated by demonstration of a consistent attack rate of 66% in the placebo vaccinated group (an attack rate of 65% had been demonstrated in the initial dose-finding study), and by demonstration of a protective effect by 3 doses of the licensed Ty21a vaccine (PE=35%). The study was performed safely, and challenge was well tolerated. The most significant study related SAE was related to use of ciprofloxacin antibiotics, rather than vaccination or illness related to challenge; the participant developed symptoms of acute depression on a background of low mood, which resolved over time without pharmacological treatment. One participant was withdrawn from the study following challenge, as they vomited (deliberately) within several hours of ingesting the challenge agent.

Building upon the experiences from these studies, the Oxford Vaccine Group has undertaken a number of additional challenge studies, including the first *Salmonella* Paratyphi A human challenge model (OVG 2013/07 Oxford A REC ref. 14/SC/0004), studies of the mechanisms and determinants of systemic and mucosal immunity to *S.* Typhi and Paratyphi A infection (OVG 2014/01 Oxford A REC ref 14/SC/1204) as well as studies testing a Vi-conjugate vaccine (OVG 2014/08 Oxford A REC ref. 14/SC/1427). In total, over 200 participants have been successfully challenged to date.

Significant advances have been made in performing these human typhoid challenge studies, including first confirmation of primary bacteraemia, identification of novel clinical and laboratory biomarkers and progression of our understanding of the host response to infection and microbiological dynamics. In this study we propose to utilise the human challenge model to further study host-pathogen interactions by investigating the role of a specific virulence factor, the typhoid toxin, in the pathogenesis of typhoid fever. This information will be used to inform vaccine design and development potentially influencing public health intervention strategies.

4.4. The Quailes strain

S. Typhi (Quailes strain) was used extensively for human challenge studies in the 1960s/70s [34,35] and has been provided by the University of Maryland to establish a master cell bank in Oxford. Prior to the development of the challenge model, study investigators met with the UK regulator (MHRA) and established the regulatory framework for challenge studies and received advice that the Quailes strain is not an Investigational Medicinal Product. Use of the Quailes strain therefore, does not require assessment or approval by the MHRA. The Quailes strain has been used in challenge studies in over 200 healthy volunteers in Oxford to date.

In order to explore the role of typhoid-toxin in the pathogenesis of enteric fever, participants will be challenged with either a wild-type Quailes strain or a toxin-deficient isogenic mutant derived from a parent Quailes strain. Two vials of GMP-grade *S.* Typhi Quailes strain were supplied by the Oxford Vaccine Group to the Galan laboratory (Department of Microbial Pathogenesis, Yale School of Medicine, New Haven, CT, USA), who undertook a targeted knock-out of the typhoid-toxin pathogenicity island, generating a variant of the parent strain that is identical save for the absence of the genes encoding the typhoid toxin. Deletion of the toxin-encoding genes was confirmed through phenotypic assays and nucleotide sequencing of the toxin encoding region. Both the wild-type and toxin-negative strains were subsequently supplied to the Walter Reed Army Institute of Research, MD, USA, who undertook manufacture to GMP standard of both strains. Full antibiotic sensitivity and microbial purity of both strains in the master cell banks was demonstrated and further characterisation work including genome sequencing has been completed at the Department of Microbial Pathogenesis, Yale School of Medicine.

4.5. Aim of the project

This research project proposes to investigate the impact of typhoid toxin in the virulence of wild type *S.* Typhi by comparison with that of a toxin-defective isogenic mutant in a human volunteer model of *S.* Typhi infection.

The primary objective of the study is to compare the proportion of participants developing clinical or microbiologically proven typhoid infection following oral challenge with wild type *S.* Typhi Quailes strain (WT), with participants challenged with a typhoid toxin-deficient isogenic mutant of *S.* Typhi Quailes strain SB6000 (TN). Extrapolating from animal and *in vitro* data, we hypothesize that the attack rate in healthy participants challenged with TN strain will be reduced compared to challenge with the WT strain. We also anticipate a milder symptom profile and less-pronounced laboratory abnormalities after challenge with the SB6000 TN strain.

The diagnostic criteria for typhoid fever are comparable with earlier challenge studies and have been designed to minimise risk to study participants by commencing treatment at the earliest possible time point [37]. As such, it is conceivable that large differences in attack rate between participants challenged with WT or TN strains may not be observed, as the illness could be halted prior to expression of the typhoid toxin or application of its biological activity *in vivo*. Both strains also possess multiple additional factors required for invasion, immune evasion and the induction of a febrile response, including expression of the Vi-capsule [25]. *In vitro* data demonstrate that typhoid-toxin expression occurs within 3-5 hours of intracellular infection and that extracellular trafficking occurs within 24 hours [30], making it highly likely that the toxin will be expressed during the course of the challenge period. The secondary objectives of this study aim to pinpoint the clinical and

immunobiological role of typhoid toxin in the pathogenesis of enteric fever, by conducting assays that can potentially detect typhoid toxin activity early during infection in addition to detailed immunological, transcriptional, metabolomic and proteomic profiles at several time points after challenge.

Controlled human infection studies such as this involve recruitment and deliberate infection of healthy adult volunteers. Strict inclusion and exclusion criteria are applied in order to protect the safety of potential participants. Consequently, the rate of volunteer exclusion prior to screening and the rate of exclusion post-screening is typically higher than that typically observed for other healthy volunteer studies conducted by our group. In order to better understand reasons for volunteer exclusion from the typhoid and paratyphoid controlled human infection studies, we aim to review and analyse data collected from screening visits. These data will allow us to adapt and review inclusion and exclusion criteria for future controlled human infection studies.

5. OBJECTIVES AND ENDPOINTS

	Objective(s)	Endpoint(s)
**Primary**	To investigate the contribution of typhoid toxin to *S.* Typhi infection and typhoid fever using a human challenge model of infection.	The proportion of participants developing clinical or microbiologically proven typhoid infection following oral challenge with 1-5x10^4 CFU wild type *S.* Typhi Quailes strain (WT) delivered in a sodium bicarbonate solution, in comparison to challenge with 1-5x10^4 CFU of a typhoid toxin-deficient isogenic mutant of *S.* Typhi Quailes strain SB6000 (TN).
**Secondary**	To compare clinical and laboratory features following challenge with either wild-type *S.* Typhi Quailes strain (WT) or typhoid toxin-deficient isogenic mutant *S.* Typhi Quailes strain SB6000 (TN).	Comparison of the clinical course of typhoid infection after challenge, between wild-type (WT) and typhoid toxin-deficient mutant (TN) *S.* Typhi Quailes strain SB6000, with particular reference to :<br>• time to onset of symptoms<br>• duration of illness including fever clearance time<br>• symptom severity<br>• time to diagnosis<br>• time to onset of bacteraemia<br>• duration of bacteraemia<br>• quantification of bacteraemia at time of diagnosis<br>• time to onset and pattern of stool shedding<br>• inflammatory response<br>• haematological and biochemical parameters<br><br>using clinical reporting, physical examination findings, microbiological assays to detect *S.* Typhi in blood and stool, and laboratory assays to monitor inflammatory responses.
	To compare the host immune response following challenge with either wild-type *S.* Typhi Quailes strain (WT) or typhoid toxin-deficient isogenic mutant *S.* Typhi Quailes strain SB6000 (TN).	Comparison of the host innate, humoral and cell-mediated responses to challenge with WT and TN strains at baseline (Day 0) and post-challenge time points, with particular reference to, but not limited to:<br>• *S.* Typhi specific antibody concentrations;<br>• Frequency and magnitude of *S.* Typhi specific antibody-secreting cells as measured by

		ex-vivo and memory B-cell ELISPOT;<br>• Description of lymphocyte populations at baseline and following challenge as measured by flow cytometry and/or CyTOF;<br>• Frequency and magnitude of *S.* Typhi specific cell-mediated immune responses as measured by ELISPOT and flow cytometry and/or CyTOF;<br>• Plasma Cytokine concentration
	To describe the variation in gene expression in whole blood following challenge with either wild-type *S.* Typhi Quailes strain (WT) or typhoid toxin-deficient isogenic mutant *S.* Typhi Quailes strain SB6000 (TN).	Comparison of differentially expressed genes using RNASeq on whole blood and/or PBMC samples at baseline (Day 0) and post-challenge time points following challenge with WT and TN strains.
	To investigate indirect effects of typhoid-toxin *in vivo.*	Analysis comparing blood and/or urine samples from participants challenged with WT and TN strains, using proteomic and metabolomics based techniques at baseline (Day 0) and post-challenge time points.
	To investigate methods for direct detection of typhoid toxin *in vivo* following challenge with wild-type *S.* Typhi Quailes strain (WT).	Exploratory analysis of blood and/or urine and/or faecal samples including use of assays including (but not limited to) mass-spectrometry.
Exploratory Objectives	To investigate how the human microbiota, including nasal carriage of *Staphylococcus aureus*, influences and interacts with a challenge of *S*. Typhi.	Samples of stool, saliva and nasal swabs to measure the constituent microbiological flora by culture and metagenomic studies.
	To investigate new molecular techniques for detection of *S*. Typhi in clinical samples.	Use of novel methodologies to prepare bacterial DNA/RNA and development of sensitive quantitative and qualitative PCR assays for blood, urine urine stool specimens.
	To investigate recruitment methods and reasons for volunteer exclusion from typhoid challenge models.	Analysis of recruitment numbers, including:

		• Number of positive and negative responses to different recruitment techniques;<br>• Number of volunteers excluded prior to attending screening visits and reasons for exclusion;<br>• Number of volunteers attending for screening visits and reasons for exclusion.

6. STUDY DESIGN

6.1. Summary of the study design

This is a double-blinded, randomised, human infection study using wild type *S.* Typhi Quailes strain (WT) or a typhoid toxin-deficient isogenic mutant of *S.* Typhi Quailes strain SB6000 (TN) in healthy adult volunteers.

Forty participants will be randomised in a 1:1 ratio to challenge with either WT or TN strains, delivered in a sodium bicarbonate solution. The primary objective of the study is to compare the proportion of participants developing clinical or microbiologically proven typhoid infection following oral challenge with WT strain, with participants challenged with the TN strain. The challenge dose of both strains will be 1-5x10^4 CFU. This dose has previously been demonstrated to give a clinical/laboratory attack rate of between 60-75% using the wild-type Quailes strain (as per the previous dose finding OVG Study 2009/10 REC ref. 10/H0604/53).

A summary of the overall trial can be reviewed in **Table 1**. The day of challenge is referred to as the zero time point (Day 0). Continuous participant safety monitoring will occur throughout the challenge period through daily clinical review and monitoring of e-diary cards.

6.2. Visit structure

After screening and on fulfilment of the study eligibility criteria, each participant will be required to attend the study site on approximately 28 further occasions over the entire study period. The timing of visits and procedures carried out on each visit are described in **Table 1** and **Table 2**. A flow chart describing the participant's journey is outlined in **Figure 1**. The protocol for visits will depend on whether the participant develops infection (i.e. meets criteria for typhoid diagnosis, 'TD') or not. These diagnostic criteria are defined

in **Table 3**. This means that an individual participant may have more or less visits in total, depending on the day of typhoid diagnosis. In addition, further reviews may be required depending on the participant's condition, clinical indication or at the discretion of the study staff. Every effort will be made to perform the visits on time as indicated in the study protocol. Based on previous experience with challenge studies and similar trial protocols, we are aware that in some instances this may be difficult. This is expected to be particularly difficult for visits TD+12 hours, TD+24 hours, TD+48 hours, TD+72 hours, TD+96 hours, as the time of diagnosis will not always be convenient for the participant to attend the study site. A window of time to complete the visits will be as follows:

- Day -7 +/- 3days
- TD+12h; TD+24h; TD+48h; TD+72h; TD+96h +/- 12 hours
- Day 28 +/- 5 days
- Day 90 +/- 14 days
- Day 180 +/- 28 days
- Day 365 +/- 56 days

Table 1 Summary of study procedures

	Screening (within 120 days)		Intensive challenge period (first 14 days)								If you develop typhoid fever		3 – 12 month follow-up	
		D-7	D0	D0+ 12hrs	D1 to D6	D7	D8 to D13	D14	D28	TD	TD+ 12hrs	TD +24, +48, +72, +96hrs	D90 and D180	D365
Informed consent	x													
Consent quiz	x													
Revalidation of consent	`	x												
Obtain 24 hr contact details		x												
Medical history	x													
Interim medical history		x	x	x	x	x	x	x	x	x	x	x	x	x
Physical examination[1]	x									x				
Vital signs	x	x	x	x	x	x	x	x	x	x	x	x	x	x
Urine pregnancy test	x	x	x					x		x				
Urine sample	x		x		x			x	x	x				
Stool sample[2]			x		x	x	x	x	x	x		x	x	x
Blood sample	x	x	x	x	x	x	x	x	x	x	x	x	x	x
Saliva sample	x	x	x					x		x				
Nasal Swab	x	x	x					x		x				
12 lead ECG	x													
Ultrasound scan	x													
Mood assessment	x	x				x		x		x				
Challenge with *S.* Typhi (WT or TN Quailes strain)			x											
Issue study pack		x												
e-Diary entries		x	x	x	x	x	x	x	x	x	x	x		
Randomisation		x												

[1] Physical examination may be performed at other times in the study if clinically indicated and at the discretion of the study team.

[2] Stool samples will be collected at least one week after completion of a 14 day course of antibiotics, until 3 successive stool samples are negative for *S*. Typhi. If persistent stool shedding occurs after completion of antibiotics, participants will be referred to the Infectious Diseases Consultant at the Oxford University Hospitals NHS Trust.

	Screening (within 120 days)		Intensive challenge period (first 14 days)								If you develop typhoid fever		3 – 12 month follow-up	
		D-7	D0	D0+ 12hrs	D1 to D6	D7	D8 to D13	D14	D28	TD	TD+ 12hrs	TD +24, +48, +72, +96hrs	D90 and D180	D365
Commence antibiotics									x		x			
Participant questionnaire										x				
Notification of Public Health England and GP[3]			x						x		x			
Letter informing close contacts			x											
Letter to close contacts offering screening									x		x			

[3] Public Health England and participant GPs will also be notified at the time of stool shedding clearance

Table 2 Sample collection plan

Sampling time points, volumes and investigations may vary. Exploratory samples not marked * may be collected in a subset of study participants only.

Investigation	Stool sample	Saliva sample*	Nasal Swab*	Urine sample*	Blood culture	Molecular Diagnostics*	Bacterial quantification	PBMC*	Serum	Plasma	Functional genomics	DNA samples (including epigenetics)	FBC	CRP, U+Es, LFTs	Screening Serology(1)	Glucose	
Sample tube	Stool Pot	Saliva pot	Nasal swab	Universal container	Aerobic BACTEC bottle	Heparin Vacutainer or Streck tube	Wampole™ Isostat® Isolator		Serum Vacutainer	Plasma vacutainer	Tempus™ Blood RNA	Taken from clot of serum tube	EDTA Vac	Serum Vacutainer		Glucometer	
Volume blood (mL)					10	10	10	25-60	5-15	3-6	3	x	1-3	2	9	1 drop	TOTAL
Day																	
Screening		x	x										3	2	9	x	14
D-7		x	x			10											10
D0	x	x	x	x				60	15	6	3	x	1	2			87
D0 +12hrs (2)					10			10	10	3	3						36
D1	x			x	10			25	10	3	3		1	2			54
D2	x			x	10				10	3	3						26
D3	x				10												10
D4	x				10			25	10	3	3		1	2			54
D5	x				10												10
D6	x				10				10	3	3		1	2			29
D7	x			x	10			60									70
D8	x				10				10	3	3		1	2			29
D9	x				10												10
D10	x				10			25	10	3	3		1	2			54
D11	x				10												10
D12	x				10				10	3	3		1	2			29
D13	x				10												10
D14	x			x	10			60	10	3	3		1	2			89

Investigation	Stool sample	Saliva sample	Nasal Swab	Urine sample	Blood culture	Molecular diagnostics	Bacterial quantification	PBMC	Serum	Plasma	Functional genomics	DNA samples (including epigenetics)	Full blood count	CRP, U+Es, LFTs	Screening Serology	Glucose	
Day																	TOTAL
TD	x			x	10	10	10		10	3	3		1	2			49
TD +12hrs	x				10												10
TD +24hrs (3)	x				10			60	5	3	3		1	2			84
TD +48hrs	x				10								1	2			13
TD +72hrs	x				10								1	2			13
TD +96hrs	x				10								1	2			13
TD Day 14	x				10			25	5	3	3		1	2			49
D28 +/- 5 days	x			x				50	15	3	3	x	1	2			74
Total for first 2 months (excluding screening)							If NO Typhoid Diagnosis										705
							If Typhoid Diagnosed										936
D90 +/- 14 days	X*							40	5		3		1				49
D180 +/- 28 days	X*							25	5								30
D365 +/- 56 days	X*							40	5			x					45
Total in 12 months							If NO Typhoid Diagnosis										829
Total in 12 months							If Typhoid Diagnosed										1060

(1) Includes Vi-antibody, Hepatitis B/ Hepatitis C/HIV serology & coeliac serology;

(2) PBMC samples at DO+12 samples to be collected in a subset of study participants

(3) PBMC samples may be collected at either the TD12, TD24 or TD48 visit to facilitate sample processing

Figure 1. Participant journeys for the duration of the study depending on typhoid diagnosis or no typhoid diagnosis.

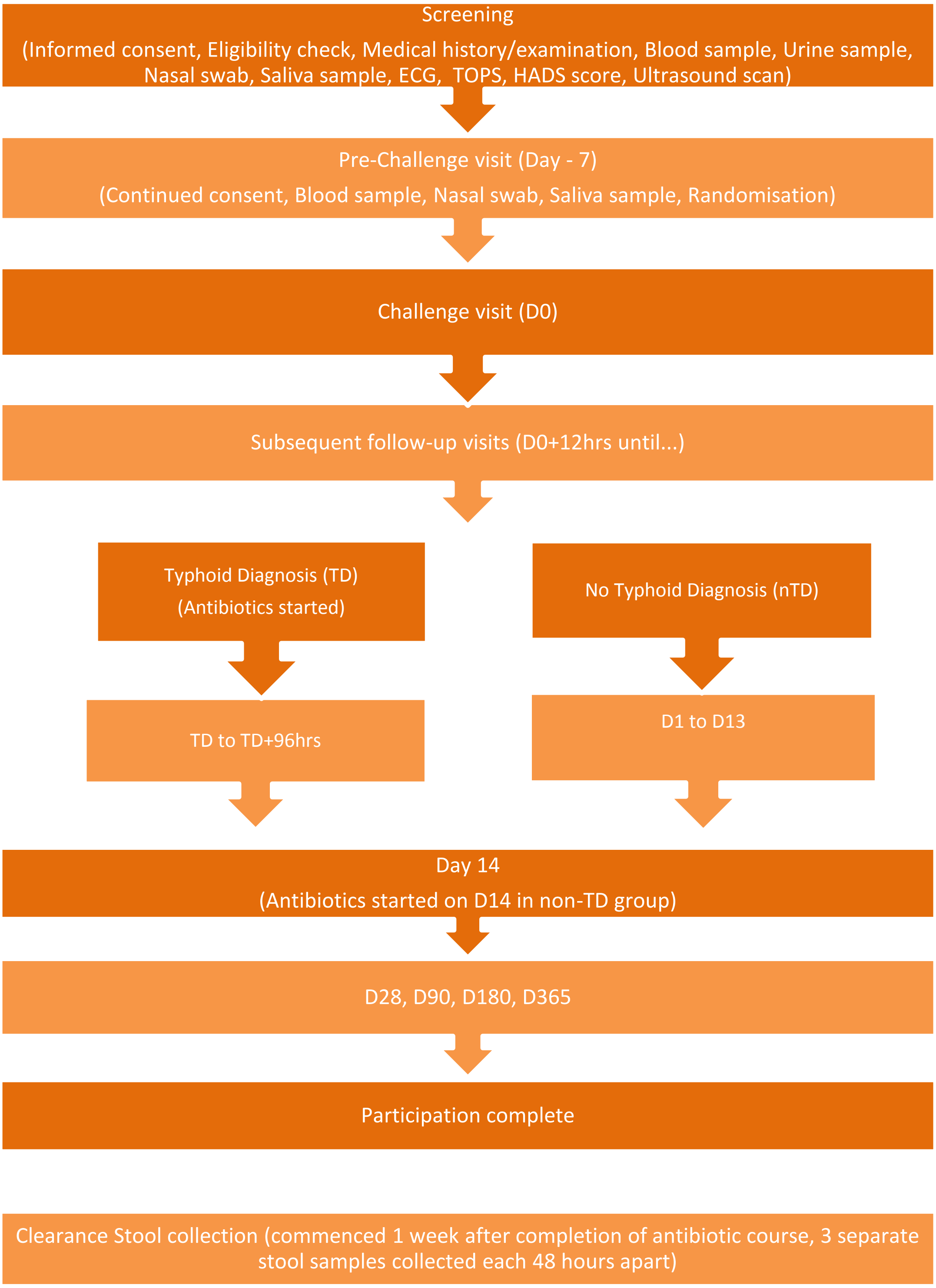

7. STUDY PARTICIPANTS

7.1. Study eligibility

Male or female participants aged 18-60 years inclusive who are in good health (as determined by a study doctor, medical investigation and review of medical history provided by their General Practitioner) and able to provide written informed consent will be eligible for inclusion in this study.

7.2. Inclusion Criteria

Participants must satisfy all of the following criteria to be considered eligible for the study:

- Agree to give informed consent for participation in the study.
- Aged between 18 and 60 years inclusive at time of challenge.
- In good health as determined by medical history, physical examination and clinical judgment of the study team.
- Agree (in the study team's opinion) to comply with all study requirements, including capacity to adhere to good personal hygiene and infection control precautions.
- Agree to allow his or her General Practitioner (and/or Consultant if appropriate), to be notified of participation in the study.
- Agree to allow study staff to contact his or her GP to access the participant's vaccination records and summary of medical history.
- Agree to allow Public Health England to be informed of their participation in the study.
- Agree to give his or her close contacts written information informing them of the participant's involvement in the study and offer them voluntary screening for *S.* Typhi carriage.
- Agree to have 24-hour contact with study staff during the four weeks post challenge and to be able to ensure that they are contactable by mobile phone for the duration of the challenge period until antibiotic completion.
- Agree to allow the study team to hold the name and 24-hour contact number of a close friend, relative or housemate who will be kept informed of the study participant's whereabouts for the duration of the challenge period (from the time of challenge until completion of antibiotic course). This person will be contacted if study staff are unable to contact the participant.
- Have internet access to allow completion of the e-diary and real-time safety monitoring.
- Agree to avoid antipyretic/anti-inflammatory treatment from the time of challenge (Day 0) until advised by a study doctor or until 14 days after challenge.

- Agree to refrain from donating blood for the duration of the study.
- Agree to provide their National Insurance/Passport number for the purposes of TOPS registration and bank account details for payment of reimbursement expenses.

7.3. Exclusion Criteria

The participant will not be enrolled if any of the following apply:

- History of significant organ/system disease that could interfere with trial conduct or completion. Including, for example, but not restricted to:
    - Cardiovascular disease
    - Respiratory disease
    - Haematological disease
    - Endocrine disorders
    - Renal or bladder disease, including history of renal calculi
    - Biliary tract disease, including biliary colic, asymptomatic gallstones or previous cholecystectomy
    - Gastro-intestinal disease including requirement for antacids, H_2-receptor antagonists, proton pump inhibitors or laxatives
    - Neurological disease
    - Metabolic disease
    - Autoimmune disease
    - Psychiatric illness requiring hospitalisation or known or suspected drug and/or alcohol misuse (alcohol misuse defined as an intake exceeding 42 units per week)
    - Infectious disease
- Have any known or suspected impairment of immune function, alteration of immune function, or prior immune exposure that may alter immune function to typhoid resulting from, for example:
    - Congenital or acquired immunodeficiency, including IgA deficiency
    - Human Immunodeficiency Virus infection or symptoms/signs suggestive of an HIV-associated condition

  - Receipt of immunosuppressive therapy such as anti-cancer chemotherapy or radiation therapy within the preceding 12 months or long-term systemic corticosteroid therapy.
  - Receipt of immunoglobulin or any blood product transfusion within 3 months of study start.
  - History of cancer (except squamous cell or basal cell carcinoma of the skin and cervical carcinoma in situ).
- Moderate or severe depression or anxiety as classified by the Hospital Anxiety and Depression Score at screening or challenge that is deemed clinically significant by the study doctors[4].
- Weight less than 50kg[5].
- Presence of implants or prosthesis.
- Anyone taking long-term medication (e.g. analgesia, anti-inflammatories or antibiotics) that may affect symptom reporting or interpretation of the study results.
- Contraindication to fluoroquinolone or macrolide antibiotics (e.g. ciprofloxacin or azithromycin).
- Female participants who are pregnant*,* lactating or who are unwilling to ensure that they or their partner use effective contraception 30 days prior to challenge and until three negative stool samples have been obtained after completion of antibiotic treatment.
- Full-time, part-time or voluntary occupations involving:
  - Clinical or social work with direct contact with young children (defined as those attending pre-school groups or nursery or aged under 2 years), or
  - Clinical or social work with direct contact with highly susceptible patients or persons in whom typhoid infection would have particularly serious consequences (unless willing to avoid work until demonstrated not to be infected with *S*. Typhi in accordance with guidance from Public Health England and willing to allow study staff to inform their employer).
- Full time, part time or voluntary occupations involving:

[4] If elevated scores are due to temporary significant life events, the questionnaire may be repeated after resolution of the event with a view to inclusion if normal.

[5] Or a Body Mass Index (BMI) that, in the opinion of the study doctors, may adversely impair the interpretation of the study results or affect the safe performance of any study procedures.

    - Commercial food handling (involving preparing or serving unwrapped foods not subjected to further heating)
- Close household contact with:
    - Young children (defined as those attending pre-school groups, nursery or those aged less than 2 years)
    - Individuals who are immunocompromised.
- Scheduled elective surgery or other procedures requiring general anaesthesia during the study period.
- Participants who have participated in another research study involving an investigational product that might affect risk of typhoid infection or compromise the integrity of the study within the 30 days prior to enrolment (e.g. significant volumes of blood already taken in previous study)[6].
- Detection of any abnormal results from screening investigations (at the clinical discretion of the study team).
- Inability to comply with any of the study requirements (at the discretion of the study staff and the participant's General Practitioner).
- Any other social, psychological or health issues which, in the opinion of the study staff, may
    - put the participant or their contacts at risk because of participation in the study,
    - adversely affect the interpretation of the primary endpoint data,
    - Impair the participant's ability to participate in the study.
- Prior vaccination with an oral typhoid vaccines (e.g. Ty21a) or other investigational typhoid vaccine (e.g. Vi-conjugate vaccine, MO1ZH09)
- Prior vaccination with a Vi-polysaccharide typhoid vaccine administered within 5 years from the time of screening.
- Prior vaccination with a Vi-polysaccharide typhoid vaccine administered more than 5 years from the time of screening AND detectable Vi-antibody titre at screening (defined as IgG ≥7.4U/ml measured using the VaccZyme *Salmonella* Typhi Vi IgG kit, Binding Site ® UK).

[6] As assessed by both participant questioning and registration with The Over Volunteering Prevention System (TOPS) database.

- Having been resident in an enteric fever endemic country for 6 months or more.
- Have previously been diagnosed with laboratory-confirmed typhoid or paratyphoid infection or been given a diagnosis compatible with enteric fever.
- Have participated in previous typhoid or paratyphoid challenge studies (with ingestion of challenge agent).
- Have a prolonged corrected QT interval (>450 milliseconds) on ECG screening.

7.4. Pregnancy and contraception

The possible adverse effects of *S.* Typhi infection or the effect of some antibiotics on the outcome of pregnancy are unknown, therefore, pregnant women will be excluded from the study. If relevant, non-pregnant female participants will be required to use an effective form of contraception from 30 days prior to challenge until deemed to be clear of infection with three negative clearance stool samples following completion of the antibiotic course.

Typhoid infection, with or without diarrhoea or vomiting, could reduce the efficacy of an oral hormonal contraceptive (‘the pill’) by altering absorption. For this reason, female participants who are taking oral contraception will be advised to use additional barrier contraception during the two week challenge period and whilst taking antibiotics.

In the event of a female participant becoming pregnant during the study, this outcome will be recorded and the Sponsor and the DSMC will be notified if appropriate. No further non-essential trial procedures will be performed (i.e. appropriate antibiotic treatment, screening for stool carriage and/or referral may be required but not further blood sampling, etc.). This is further addressed in Section 11.7 and the procedure as outlined in relevant OVG SOPs will be followed.

7.5. Trial Over-volunteering Prevention System

The Over-volunteering Prevention System (TOPS) is a database to guard against the potential for harm that can result from excessive volunteering in clinical trials involving IMPs or blood donations. Participants will be registered with this system at screening using their national insurance number or passport number if they do not have a national insurance number. The system will be updated in the event of the participant not entering the trial, being withdrawn or excluded. Alternatively, TOPS will be updated on the participant’s last visit.

7.6. Potential benefits to participants

Participants will not directly benefit from participation in this study. However, it is hoped that the information gained from this study will contribute to the development of an understanding of enteric fever. The only benefits for participants will be information about their general health status.

7.7. Potential risks to participants

The general risks to participants in this study are associated with study-fatigue, phlebotomy, symptomatic infection and the small risk of subsequent complications.

Study-fatigue

This may occur due to the intense nature of the study procedures, especially during the challenge period. Participants are reimbursed for their time and every effort will be made to make study investigations as swift and uncomplicated as possible. Interventions and visits will be limited in number and will be arranged to fit with individual schedules and other obligations, as far as is practical.

Phlebotomy

The volume of blood drawn over the study period should not compromise healthy adult participants. No more than 1135ml of blood will be taken over the course of the study. As a comparison, women are able to donate a maximum of 1410ml of blood per year, and men 1880ml, to the National Blood Service. Mild tenderness, bruising, light-headedness or, rarely, syncope may result from venepuncture.

Symptomatic infection

Some study participants will develop symptomatic typhoid infection following challenge, as demonstrated in the initial dose-finding study. During the challenge phase (before treatment with antibiotics) participants will be reviewed at least daily by a clinical study team member and will, in addition, be contacted (by text message) every evening to ensure participant safety, and to reiterate that participants are to contact the study team if they have any concerns. Participants will be made aware of the potential symptoms of typhoid infection and will be monitored closely throughout the challenge for the development of these symptoms. Symptoms of fever, headache, malaise, anorexia, abdominal pain, nausea/vomiting, myalgia, arthralgia, cough, rash, diarrhoea and constipation will be actively enquired about at each visit after challenge. Participants will be provided with a thermometer and will be instructed to record their oral temperature a minimum of twice a day and to contact study staff immediately should they record an elevated temperature ($\geq 38^{o}C$) or if they have any concerns.

The further management of typhoid fever is outlined in section 8.12. If any participant is unexpectedly unwell, the clinical study team will arrange an additional review. If it is deemed necessary for safety

reasons, a study nurse or doctor will be permitted to review the participant in their home. This will be emphasised at screening and throughout the study.

Complications of typhoid fever, such as perforation or haemorrhage, occur almost exclusively in patients who do not receive appropriate antibiotic treatment for an extended period of time. Participants in this study will be treated 12 hours after developing fever or if *S.* Typhi is recovered from a blood culture drawn 72 hours or more after challenge (see section 8.13). They will be closely monitored during the initial study phase until a 14 day course of antibiotics is completed to minimise the risk of complications. Participants who are not diagnosed with typhoid fever within 14 days post-challenge will also be treated with a 14 day course of antibiotics.

The risks associated with typhoid challenge will be greatly minimised by complying with study visits and maintaining close contact with the study team. Previous challenge studies using the same parent strain of *S.* Typhi (Quailes strain) undertaken in the 1960/70s at the University of Maryland and at OVG have demonstrated a good safety profile. This will be the first study using an attenuated typhoid-toxin negative variant of the *S.* Typhi Quailes strain SB6000 (TN), which has been manufactured to GMP standard for the purposes of this study. It anticipated that the TN strain will have an equivalent safety profile to the WT parent strain.

Relapse of typhoid fever

Relapse rarely occurs after treatment with an effective antibiotic for 14 days; shorter durations of therapy have been associated with infrequent relapses [19,38]. For this reason, a two-week antibiotic course of oral ciprofloxacin will be used. Participants will be screened for shedding of *S*. Typhi in the stool commencing one week after completion of antibiotic therapy and reviewed if symptoms or laboratory results suggest evidence of relapse.

Chronic carrier state

A chronic carrier state, in which *S.* Typhi is excreted in the stools for many years without symptoms, can develop after *S.* Typhi ingestion [19,39]. The chronic carrier state is usually seen in older women with pre-existing gallbladder disease (primarily gallstones). Only participants with a normal ultrasound examination of the gallbladder will be included (see section 7.3) in this study. The likelihood of developing chronic carriage is extremely low, particularly with newer antibiotics such as fluoroquinolones and azithromycin being available. A previous study demonstrated that, of more than 200 patients treated for typhoid fever with ciprofloxacin, none became carriers [38].

To ensure clearance of infection and to exclude carriage, stool samples for culture will be obtained upon completion of the initial antibiotic course. Should convalescent carriage occur (defined as stool cultures

being positive for *S.* Typhi in at least 1/3 stool samples collected at least one week after completion of antibiotics) then participants will be referred to an Infectious Diseases Consultant at the Oxford University Hospitals NHS Trust for further management.

Antibiotics

Potential participants with known antibiotic hypersensitivity or allergy to either of the first-line antibiotics (ciprofloxacin, azithromycin or other macrolide antibiotics) will be excluded. The antibiotics to be used in this study are generally well tolerated and are only occasionally associated with side effects. Should an antibiotic cause allergy or intolerance this will be managed by a study doctor and a different antibiotic will be used for subsequent management.

7.8. Potential risks to close contacts of participants

In view of the low infectivity of *S.* Typhi without bicarbonate buffer and the high standard of hygiene and sanitation in the UK, secondary transmission of the challenge strain to household or other close contacts after discharge is highly unlikely. It is thought that *S.* Typhi, unlike *Shigella sp.*, enterohaemorrhagic *Escherichia coli* or hepatitis A virus, is virtually never transmitted by direct faecal-oral contact. This is in part due to the higher oral inoculum of *S.* Typhi bacteria required to cause clinical disease.

The only (rarely) reported exception is direct transmission by ano-lingual sexual contact [7]. Although the risk of typhoid transmission to sexual contacts is very uncommon, participants will be informed of which bodily fluids are potentially infective and will be advised to use barrier methods to minimise the risk of transmission.

It is acknowledged, however, that transmission within households can occur if the individual excreting *S.* Typhi fails to practice effective hand washing after defecation and is subsequently involved in uncooked food preparation. If food is kept at ambient temperatures, bacterial proliferation occurs such that an infective dose level is reached, and the food then may act as a vehicle for typhoid transmission.

Throughout the period of possible excretion of the challenge strain, participants must practice stringent hand washing techniques, particularly after defecation. Participants will be given soap and paper towels for use at home and detailed advice on how to prevent transmission of *S.* Typhi. Participants will be taught and observed practising good hygiene technique at their initial challenge visit. The importance of adhering to sanitation advice will be emphasised to participants at each visit. It is important to note that participants in this trial will be fully informed about the risks of transmission and how to prevent this prior to challenge. As such, participants will be in the position to implement this from the point of infection, which will reduce the chance of secondary transmission. This is very different from the situation with travellers returning

from abroad where typhoid diagnosis is usually delayed by several weeks allowing a prolonged period of exposure to contacts before precautions are put in place. Since most individuals living in developed countries practice good personal hygiene and food hygiene, secondary transmission of *S*. Typhi within households by returning travellers with typhoid fever is rare. Furthermore, the delay in diagnosis that occurs in travellers with typhoid fever leads to a prolonged period of time in which *S.* Typhi has been excreted. We will treat all participants in this study very early in the course of disease, leading to rapid clearance of bacteria and a very limited period of excretion, reducing potential exposure to contacts.

When occasional transmission of typhoid infection occurs, it is usually related to unknowingly infected food handlers [39,40]. For this reason, food handlers will be excluded from this study. Potential participants employed in clinical or social work with direct contact with young children (those attending pre-school groups, nursery, or aged less than 2 years) or highly susceptible patients or persons in whom typhoid infection would have particularly serious consequences (such as the elderly) also represent an increased risk and will be excluded unless willing to not work until it has been demonstrated that they are not infected with *S.* Typhi in accordance with PHE guidance [10].

Even in the absence of precautions to prevent secondary transmission (as is seen in returning travellers), the rate of transmission is exceptionally low within the UK. In a large recent study of 251 contacts of patients with typhoid fever in London, only one patient was identified as a suspected case of secondary transmission [41]. Similarly, a study in Scotland showed a maximum secondary transmission rate in the absence of precautions to be 6 out of 267 contacts [42].

8. TRIAL PROCEDURES

Existing OVG and OVC Standard Operating Procedures (SOPs) govern the safe and accurate conduct of all study procedures in addition to a study-specific Clinical Study Plan.

8.1. Recruitment

Identification of study participants

Several strategies may be employed in order to recruit the required cohort of participants, including:

- NHS database: Potential study participants will be identified via databases such the National Health Applications and Infrastructure Services (NHAIS) who hold the central NHS patient database (Open Exeter) or their equivalent. Use of the database will be approved by the Caldecott Guardian for that specific area. These databases will identify all persons within the local area who are in the appropriate age range. First contact to potential participants will not be made by the researchers at the Oxford Vaccine Group. The initial information about the study will be sent out from this agency to preserve the confidentiality of potential participants. Potentially eligible participants will be sent an invitation letter, which describes the study, with a reply slip. Anyone who is interested in taking part will be able to contact the study team by telephone, email, registering on the OVG website or by returning the attached reply slips. Potential participants will be directed to the full information booklet on the OVG website, or will be sent the information via post or email. The study team will then contact them via telephone and/or email to provide further details about the study and arrange a screening visit. Those people who have indicated they do not wish to receive postal mail−shots will have their names removed prior to sending the invitations.
- Poster advertising: Display of posters advertising the study throughout local hospitals and doctor’s surgeries, tertiary education institutions and other public places with the permission of the owner/ proprietor.
- Direct mail-out: This will involve direct mailing of the study information sheet to adults whose names and addresses have been obtained via the Electoral register. Those people who have indicated they do not wish to receive postal mail−shots will have their names removed prior to the study team being given the names and addresses. The company providing this service is registered under the Data Protection Act 1998.
- E-mail communication: We will contact representatives of local tertiary education establishments and local employers and ask them to circulate posters and information, and to circulate a link to study information on the OVG website by email.

- Oxford Vaccine Centre (OVC) database for healthy volunteers: Direct email and link to members of the public who have registered their interest in potentially volunteering for clinical trials conducted by OVC. This secure database is maintained by OVC and members of the public registered here have given consent to have their details recorded and be contacted expressly for this purpose of being notified when a trial opens for recruitment. They understand this is not a commitment to volunteering for any trial they are contacted about.
- Media advertising: Local media, newspaper, radio, website and social media advertisement placed in locations relevant for the target age group with brief details of the study and contact details for further information.
- Website advertising: Description of the study and copy of information booklet on the OVG website.
- Exhibitions: Advertising material and/or persons providing information relating to the study will exhibit using stalls or stands at exhibitions and/or fairs, such as University Fresher's Fairs.
- Royal Mail Leaflet: Royal Mail door-to-door service with delivery of invitation letters in OVG envelopes to every household within certain postcode areas.

Recruitment, approach and initial eligibility assessment of potential study participants

Potential participants who are interested in study participation will be able to contact OVG by telephone, email, by the OVG website online registration with self-screening questions or paper reply slip for further information. Once an expression of interest has been received by OVG, an information booklet will be sent via mail or email to the potential participants to read at their leisure. Participants can also be directed to the OVG website, where the information booklet will be available. If participants are willing to proceed they are invited for a screening and consent visit, where a member of the clinical research team at the Oxford Vaccine Group will assess their eligibility. We will also take consent for clinical staff to access electronic patient records (EPR) to assess eligibility.

8.2. Informed Consent

The participant will personally sign and date the latest approved version of the informed consent form before any study specific procedures are performed. Consent will be sought as described in relevant OVG and OVC SOPs. Written and verbal versions of the participant information booklet and informed consent form will be presented to the participant, detailing no less than:

- the exact nature of and the rationale for performing the study

- implications and constraints of the protocol
- the risks and benefits involved in taking part

Additional consent will be sought from each participant:

- For a study staff member to hold the name and 24-hour contact number of a close friend, relative or housemate who lives nearby and will be kept informed of the study participant's whereabouts for the duration of the study. This person is to be contacted if study staff are unable to contact the participant. The 24-hour contact will receive written information, and complete and sign a reply slip that the participant will give the study doctor/nurse before challenge.
- To inform close contacts of their involvement in the study they will be given letters provided by OVG. The letter details the study, and offers screening for typhoid carriage. The low risk of spread will be emphasised to contacts to avoid undue anxiety.
- For Public Health England to be informed of study participation, challenge outcome, commencement and completion of antibiotic course, and clearance results.
- To contact their GP to confirm their medical and immunisation history and participation in the study.
- If the participant is involved in the provision of health or social care to vulnerable groups then consent will be taken to inform his/her employer of their participation in the study.

It will be clearly stated that the participant is free to withdraw from the study at any time, for any reason and that they are under no obligation to give the reason for withdrawal. The participant will be allowed adequate time to consider the information, and the opportunity to question the researcher, their GP or other independent parties to decide whether they will participate in the study. Written informed consent will be obtained by means of a dated signature of the participant and a signature of the study staff member who presented informed consent. A copy of the signed informed consent will be given to the participant and the original signed form will be retained at the study site. A doctor or nurse at OVG, who has been trained in the consent process, will conduct the informed consent discussion. Participants will be asked to complete a consent quiz after completing the informed consent form to ensure they have properly understood the study and re-education will be performed if necessary.

8.3. Screening and Eligibility Assessment

Once informed written consent is obtained, the following baseline assessments and information will be collected as part of the assessment of inclusion/exclusion criteria:

- Participant demographics; age, sex and ethnicity.
- Medical history, including:
    - Details of any significant medical or surgical history based on participant recall. If medical clarification is required, medical notes may be obtained and/or discussion with other medical practitioners will be undertaken.
    - Immunisation history (particularly receipt of previous typhoid vaccines).
    - Specific details relating to risk factors for *S. aureus* carriage (e.g. smoking history, previous antibiotic usage, previous skin infections/abscesses).
    - Confirmation from the participant's GP that they know of, or do not know of, any medical condition that may affect their suitability as a participant.
    - Blood donation history and planning.
- Females participants only:
    - Contraception use.
    - Urinary pregnancy test.
- Responses regarding any personal or domestic reason that may lead to concern regarding a participant's ability to maintain good personal hygiene.
- Use of concomitant medication (including over the counter medications, vitamins, illicit drug use and herbal supplements).
- History of living in or visiting enteric fever endemic areas (age, place and duration).
- Physical examination; cardiovascular, respiratory, abdominal and gross neurological examination, calculation of Body Mass Index, blood pressure, heart rate and temperature.
- Urine dipstick (and laboratory analysis if appropriate).
- 12-lead ECG.
- Blood samples for: haemoglobin count, white cell indices, platelet count, serum sodium, serum potassium, serum urea, serum creatinine, liver function tests, C-reactive protein, serum amylase, tissue transglutaminase antibody and IgA levels, HIV, Hepatitis B and C serology, and Vi (typhoid) serology.
- Random capillary or venous blood glucose.
- Nasal swab and saliva sample to assess microbiota, including *S. aureus* carriage.

- Abdominal ultrasound (to screen for gallbladder disease).
- Mood assessment by the Hospital Anxiety and Depression Score.
- Provision of the following documents;
    - 24-hour contact letter (to be returned completed and signed),
    - Letter to participant's close contacts.

The medical history, vaccination history and prescribed medication lists are based primarily on participant recall. With prior participant approval, the GP will be contacted to confirm the history as given and stating whether they know of any medical reason why the participant should not be included; this reply is required prior to study enrolment.

Consent will be taken to register the participant onto TOPS (see section 7.7).

Participants will be informed that they would also be eligible for BioBank ('Oxford Vaccine Centre Biobank' Southampton & South West Hampshire LREC (B) 10/H0504/25). BioBank is a separate study and optional to all participants of studies conducted by OVC. Separate consent is sought for this.

All laboratory results will be reviewed and collated by the study team who will record these in the electronic source database. Specific guidance is provided in existing OVG SOPs. If a test result is deemed clinically significant, it may be repeated, to ensure it is not a single occurrence. If a test remains clinically significant, the participant will be informed and appropriate medical care arranged with the permission of the participant in liaison with their GP. Decisions to exclude potential participants from enrolling in the trial or to withdraw a participant from the trial will be at the discretion of the Chief and Co-Investigators.

8.4. Definition of enrolment

A participant is considered enrolled at the time of the Day -7 visit. A maximum period of 120 days between initial screening and enrolment into the study will be accepted. If the period between screening and enrolment exceeds 120 days, rescreening will consist of repeat informed consent, interim medical history, clinical examination, urinalysis and blood tests as a minimum. Additional screening investigations, including repeat ultrasound gallbladder scan, may be performed at the discretion of the study doctor following consultation with the senior study investigator.

8.5. Pre-Challenge visit

Participants will be given access to an e -Diary from seven days prior to challenge. During this time we will record any unsolicited symptoms and medications. From the day of challenge through to day 21 after

challenge (28 days in total), participants will record daily solicited symptoms and temperature. The e-Diary will remain accessible till day 28 post-challenge for unsolicited symptoms. Completion of the e-Diary prior to challenge will comprise of recording unsolicited symptoms only, with the aim of aiding interpretation of the post-challenge data.

Participants will be provided with study centre contact details (including 24-hour telephone contact details for the on-call study doctor). They will also be issued with a Medic Alert-type card containing information including the antibiotic sensitivity of the *S.* Typhi strain, study doctor contact details and instruction for the research team to be contacted immediately in the event of illness/accident.

A blood sample will be collected on this day and we will perform a urine pregnancy test on female participants.

We will also undertake a baseline pre-challenge mood assessment using Hospital Anxiety and Depression Score (HADS).

8.6. Randomisation, blinding and code-breaking

Randomisation of challenge agent will be carried out at the OVG on Day -7. Randomisation will use varying block sizes (stratified by Vi antibody titre at baseline) with an allocation ratio of 1:1 to receive the wild type or toxin-negative knock-out strain using the on-line computer randomisation system 'Sortition'. The exception will be the sentinel group of two participants who will be randomised 1:1 to receive wild type or toxin-negative strains using a block size of two.

Challenge agent blinding

The study will be conducted double-blind from the time of randomisation until participant unblinding, which will occur once the last participant has completed their Day 28 post-challenge visit. The challenge agents will be administered in a double-blinded fashion, such that the participant and clinical staff undertaking follow-up visits will not be aware of which challenge agent they have received (wild-type or toxin-negative knock-out strain). Trained unblinded laboratory staff will prepare the WT and TN challenge agents on Day 0, in accordance with local SOPs. Both WT and TN strains will be prepared suspended in sodium bicarbonate and will have an indistinguishable appearance (transparent, colourless liquid). Challenge strains will be delivered to the clinic rooms labelled with participant identification number.

Clinical staff administering the challenge agents will be aware of challenge allocation. Aside from designated unblended laboratory staff preparing the challenge agents, other laboratory staff members will remain unaware of challenge agent assignment. Participants will be informed by the study team of the

challenge agent they have received at the time of unblinding. A letter will also be sent to the participant's GP notifying them of unblinding and challenge agent assignment.

Unblinding may occur at an earlier time point in the event of participant withdrawal, or the occurrence of SAEs, SARs or SUSARs. This will be conducted under the guidance of the Data Safety and Monitoring Committee.

8.7. *S.* Typhi Challenge Procedure

Assessment and challenge with either wild type *S.* Typhi Quailes strain (WT) or typhoid toxin knock-out *S.* Typhi Quailes strain SB6000 (TN) will take place on Day 0. These procedures are described below, and in further detail in the relevant OVG SOPs, Clinical and Laboratory Study Plans.

Assessment

- Obtain interim medical history; check observations and perform physical examination if clinically indicated; complete continued consent form; check eligibility criteria (specifically for temporary exclusion criteria to challenge) and check for occurrence of any new adverse or medically significant events since previous visit.
- Check details of the 24-hour contact (who will be kept informed by the participant of their whereabouts for the subsequent 28 days) (see section 8.2).
- Record oral temperature, pulse and blood pressure.
- Perform urinary pregnancy test for all females.
- If the participant is in good health and is still suitable for inclusion in the study, perform blood draw as per **Table 2** (section 6.2), relevant OVG SOPs and Clinical Study Plan.
- Collect clinical specimens as per **Table 2.**
- Confirm the participant has fasted for a minimum of 90 minutes prior to ingestion of the challenge agent.

Preparation of challenge agent

The solution for ingestion containing either wild type *S.* Typhi Quailes strain and typhoid toxin knock-out *S.* Typhi Quailes strain will be prepared in a Class II biological safety cabinet within containment level 3 laboratory that is solely used for the purpose of preparing the challenge solution. Challenge solution preparation is conducted by laboratory staff and checked by two laboratory staff members. Two clinical study team members check the challenge solution immediately prior to ingestion by the participant. The

water and bicarbonate used for the preparation will be commercially available food products. The strain will be prepared, checked and given to participants as outlined in relevant OVG SOPs and Laboratory and Clinical Study Plans. Following solution ingestion, the single-use containers will be returned to the laboratory for inspection, autoclaving and disposal.

Administration of *S.* Typhi (Quailes strain)

Both *S.* Typhi challenge agents used in this study will be administered by the oral route with sodium bicarbonate at a dose of 1-5x10^4 CFU. Participants will fast for 90 minutes before and after challenge. The procedure for administration is:

- Remove the *S.* Typhi suspension from the BIOJAR.
- Check label to ensure contents of solution match challenge agent allocation.
- Ask the participants to drink the 120 ml of bicarbonate solution.
- Ask the participants to ingest the 30ml *S.* Typhi/bicarbonate solution.
- Return containers to the containment level 3 laboratory for disposal in accordance with local OVG SOPs.

Assessment after challenge

- Fast participant for a further 90 minutes.
- Participants who vomit for any reason within 90 minutes of the challenge will be withdrawn from the trial and treated with antibiotics as described in section 8.12.
- Instruct participant to notify study centre of any serious adverse events/reactions that occur prior to next review.
- Instruct participant not to use antipyretics.
- Instruct participant to notify study team if they require the use of any medications.
- Instruct participant to notify study centre if they have a temperature ≥38.0°C.
- Provide participant with access to an eDiary and paper back up diary for recording systemic effects and oral temperatures.
- Check details of a mobile telephone number that the participant will be carrying with them for the 14 days post-challenge. Counsel the participant on the importance of keeping the mobile switched on and with them at all times. Explain to the participant that in the event that they are

uncontactable, and their 24 hour contact is unsure of their whereabouts, the study team will make every effort to locate them. This may include notifying the police.

- Issue participant with information on enteric precautions.
- Educate participant on correct hand washing technique, including demonstration and observation.
- Advise participant to inform the study team if any breaches of enteric precautions occur such that another individual comes into contact with excreta from the participant.
- Issue participants with liquid hand soap and paper towels to aid with adherence to enteric precautions.
- Instruct participant on obtaining stool specimens (as outlined in the relevant OVG SOPs) and provide the participant with sampling equipment.

Notification of challenge will be provided to Thames Valley Health Protection Unit (Public Health England) and to the participant's GP.

Participants do not have to remain on site between assessments but a rest area will be provided which participants may use if they wish.

8.8. Post-challenge visits; non-typhoid diagnosis visits (D0+12h to D365)

These visits will require the following procedures:

- Obtain and document interim medical history since screening and check continued consent,
- Review e-diary entries and laboratory blood results (if available),
- Record oral temperature, pulse and blood pressure,
- Perform mood assessment using HADS form (only on Day 7, and the day of antibiotic commencement either for typhoid diagnosis or on Day 14 after challenge if no typhoid diagnosis is made),
- Perform sample collection as per **Table 2**,
- Prescribe and issue concomitant medication for symptom control if required (see **Table 6**),
- Schedule next visit and reiterate participant requirements such as completion of the eDiary, refraining from use of antipyretics, notification of any medication administration, and notification of any fever ≥38.0°C (where appropriate).

8.9. Outside of scheduled visits and unscheduled visits

Unscheduled visits will be arranged, if required, to ensure participant safety as further history, examination and investigation may be needed. These visits will be at the discretion of the clinical study team. If participants are unwell and unable to attend CCVTM for a visit, they may be visited at home by one of the study team members.

8.10. Typhoid diagnosis and typhoid diagnosis visits (TD to TD+96 hours)

These visits will require the following procedures:

- Obtain and document typhoid diagnosis including physical examination findings (at TD and at TD+12 to TD+96 if applicable),
- Assessment by a study doctor at the time of TD to assess severity and potential need for in-patient admission **(**Table **5)**, further visits will be conducted by a clinical study team member,
- Review e-diary entries and laboratory results (if available),
- Record oral temperature, pulse and blood pressure,
- Perform sample collection as per **Table 2**,
- Prescribe and issue concomitant medication for symptom control (if required) and antibiotic therapy (with confirmatory negative urine pregnancy test result at TD) (see Table 6),
- Schedule next visit and re-iterate participant requirements such as continuing e-diary entries, adherence to antibiotic therapy and maintaining contact with the study team.

Diagnosis of typhoid infection

For the purposes of data analysis and reporting of typhoid cases to Public Health England, typhoid infection will be defined as specified in **Table 3**.

Typhoid fever is diagnosed if ANY of the following apply
**A positive blood culture for *S.* Typhi from 72 hours post-challenge**
**A positive blood culture for *S.* Typhi within 72 hours post-challenge, with one or more signs/symptoms of typhoid infection (such as recorded temperature ≥38.0°C)**
**Persistent positive blood cultures (two or more blood cultures taken at least 4 hours apart) for *S.* Typhi within 72 hours post-challenge**
**Oral temperature ≥38.0°C persisting for 12 hours**

Table 3. Criteria for the diagnosis of typhoid infection

S. Typhi bacteraemia occurring before 72 hours may reflect a transient primary bacteraemia and not typhoid fever; however, participants who are bacteraemic before 72 hours AND have one or more symptoms/signs consistent with typhoid infection (such as a temperature ≥38.0°C) will also be deemed to have reached the definition for typhoid fever.

Microbiologically, the earliest indication of *S.* Typhi bacteraemia will be the identification of Gram-negative bacilli by Gram staining of aerobic blood/broth culture specimens. Formal identification of the organism as *S.* Typhi, will take a minimum of a further 24-hours. Participants in whom a Gram-negative bacillus is identified in the aerobic blood culture bottle will, therefore, be defined as having typhoid fever for the purposes of clinical management (including antibiotic treatment) and collection/handling of blood, urine, and stool samples.

Severe typhoid fever

Severe typhoid fever will be defined as illness that includes any of the criteria listed in **Table 4**.

Severe typhoid fever is diagnosed if ANY of the following apply
**Oral temperature ≥ 40.0°C**
**Systolic blood pressure ≤ 85 mmHg**
**Significant lethargy or confusion**
**Gastrointestinal bleeding**
**Gastrointestinal perforation**
**Any grade 4 or above laboratory abnormality, as defined in Appendix C: Grading the severity of laboratory Adverse Events**

Table 4. Criteria for severe typhoid diagnosis

Admission to in-patient facility

Admission to the John Warin Ward (or Infectious Diseases Unit, Oxford University Hospitals NHS Trust) will be considered by a study doctor under the following circumstances:

Admission to in-patient facility to be considered if ANY of the following apply
**Severe typhoid fever as defined in Table 4**
**Failure of symptoms to improve within 72 hours after starting antibiotic therapy**
**Inability to tolerate oral antibiotics**
**Dehydration requiring intravenous fluid therapy**
**Unanticipated concerns about the participant's home conditions**

Table 5. Criteria for considering admission to inpatient facility

In addition, any participant that deviates from the protocol, including taking antipyretics prior to a diagnosis of typhoid infection is made, will be considered for hospital admission at the discretion of the clinical study team.

Ultimately, all decisions regarding admission will be assessed by the clinical study team in conjunction with the Infectious Diseases Consultant on-call. The consultant will be made aware of the study protocol and the suggested treatments outlined below, but in-patient management is at the discretion of the supervising consultant.

Blood sampling for participants with typhoid fever

For participants who develop typhoid fever, blood tests will be performed as per Table 2 rows labelled Typhoid Diagnosis (TD). This schedule replaces other scheduled bloods during these days. After completing the visit TD+96 hours, the next blood sample will occur on the Day 14 visit unless further blood samples are required for participant safety purposes (e.g. positive blood culture at TD+96 visit). If the TD+96 hours visit falls on or after Day 14 post challenge, then the next blood sample will be collected at the Day 28 visit.

8.11. Reporting to the Health Protection Unit

The Thames Valley Health Protection Unit (Public Health England) will be informed of the name, address and date of birth of all participants who;

- have been challenged with *S.* Typhi
- satisfy the definition of typhoid infection
- have commenced and completed antibiotics, and
- have completed clearance stool sampling (with additional information and continued contact if persistence stool shedding occurs).

In addition any breaches in enteric precautions that result in another individual coming into contact with the excreta of a participant will be reported to Public Health England.

8.12. Concomitant medication for symptoms of typhoid infection

Concomitant medication can be provided for symptomatic control of typhoid infection, before and after diagnosis. Concomitant medication after typhoid diagnosis includes antibiotics and antipyretics if required.

Drug	Indication	Dose	Route	Frequency
Paracetamol	Fever and discomfort (**after antibiotic therapy started**)	500mg - 1 g	Oral	PRN, max QDS
Codeine	Pain including headache	30-60mg	Oral	PRN (max. 240mg/24 hours)
Senna	Constipation	2-4 tablets	Oral	PRN
Domperidone	Nausea and/or vomiting	10-20mg	Oral	PRN (max. 80mg/24 hours)
Chlorpheniramine	Allergy	4mg	Oral	PRN TDS-QDS (max. 24mg/24 hours)

Table 6. Concomitant medication for symptom control

8.13. Antibiotic treatment

Antibiotic therapy is commenced when one or more of the criteria in **Table 7** are satisfied.

Antibiotics are commenced if ANY of the following apply
Any participant meeting the definition of typhoid infection
Any participant with 3 or more of the following symptoms ***severe enough to interfere with all normal activity;***<br>• **Headache**<br>• **Malaise**<br>• **Anorexia**<br>• **Abdominal pain**<br>• **Nausea/vomiting**<br>• **Myalgia**<br>• **Arthralgia**<br>• **Cough**<br>• **Rash**<br>• **Diarrhoea**<br>• **Constipation**
Any participant who has not received antibiotics by day 14 post-challenge
Any participant in whom antibiotic use is felt to be clinically necessary (as decided by a medically qualified study doctor)

Table 7. Criteria for commencing antibiotic treatment.

The first line antibiotic is oral Ciprofloxacin 500mg twice daily for 14 days. Side-effects of ciprofloxacin are detailed in the British National Formulary. The most common side-effects include gastro-intestinal upset, rash, dizziness or itch. Participants will be informed of possible side-effects of antibiotic treatment before enrolment into the study. Cautions and contra-indications for the use of ciprofloxacin include:

- Pregnancy – a negative pregnancy test is required of all female participants of childbearing potential prior to treatment,
- Quinolone antibiotics can cause QT interval prolongation, increasing the risk of ventricular arrhythmias in people predisposed to QT prolongation. For this reason, participants with

evidence of QT interval prolongation on screening ECG or a history of cardiac arrhythmia will be excluded from the study.

Any participant in whom a contra-indication to ciprofloxacin becomes apparent (as per The British National Formulary) or if response to treatment is slower than expected, the following regimens of licensed antibiotics will be used:

- 2nd line: Oral azithromycin 500mg once daily for 14 days
- 3rd line: Oral trimethoprim/sulfamethoxazole 160/800 mg once daily for 14 days
- 4th line: Oral amoxicillin 500mg TDS for 14 days

Participants will be asked to take one antibiotic dose per day in the presence of a study member, or, if the participant is not due to visit the CCVTM when an antibiotic dose is due, they will be telephoned or contacted by text message to remind them to take the antibiotic dose. The participant's GP will be notified in writing of the antibiotics received.

8.14. Stool culture for carriage of *S.* Typhi

To detect chronic carriage of *S*. Typhi, stool samples for culture will be obtained one week after completion of the antibiotic course until three samples (each taken at least 48 hours apart) are negative. Once these criteria are satisfied, the participant will be considered to be fully treated for *S.* Typhi infection and no longer an infection risk. If samples remain positive for *S.* Typhi four weeks after completion of antibiotics then the participant will be referred to a Consultant in Infectious Diseases (Oxford University Hospitals NHS Trust) for further management.

Public Health England will be informed of all participants in whom clearance has been demonstrated and of any participant who fails to demonstrate clearance after the initial 14 day course of antibiotics or after any other antibiotic treatment. The employer of any participant involved in the provision of health or social care to vulnerable groups will be notified in writing once three successive stool samples are negative.

8.15. Screening of close contacts for carriage of *S.* Typhi

The participant will provide letters from the study team to close contacts including household contacts. Contacts will be offered the opportunity to be screened for *S*. Typhi infection, which will involve obtaining two stool samples 48-hours apart. If either stool culture of a household contact is positive, he/she will be

referred to a Consultant in Infectious Diseases for appropriate antibiotic management and Public Health England will be informed.

8.16. Transport of samples

All samples from participants must be a labelled with a 'Danger of Infection' sticker if transported outside of the CCVTM. If a specimen sample bag is to be used, this should also be labelled 'Danger of Infection'. Samples should be transported in accordance with local OVG SOPs.

8.17. Discontinuation/ Withdrawal of trial participants

Each participant can exercise his or her right to withdraw from the study at any time. If, however, the participant decides to withdraw after they are challenged they will be, for public health reasons, required to complete a course of antibiotics and may be required to attend additional hospital/non-study visits to ensure compliance. In addition, the investigator may terminate a participant's involvement in the study at any time if the investigator considers it necessary for any reason including, though not exclusive to, the following:

- Ineligibility (either arising during the study or in the form of new information not declared or detected at screening),
- Significant protocol deviation,
- Significant non-compliance with study requirements or risk to public health,
- Any adverse event which requires discontinuation of the study procedures or results in an inability to continue to comply with study procedures,
- Consent withdrawn,
- Lost to follow-up.

Withdrawal from the study will not result in exclusion of the data generated by that participant from analysis. The reason for withdrawal, if given, will be recorded in the CRF.

8.18. Participant questionnaire

Following completion of their last e-diary entry, participants will be directed to an optional questionnaire regarding their experience of the study with the results anonymised. The questionnaire will be closely based on one used in the previous typhoid study OVG 2011/02 (approved by the South Central, Oxford Research Ethics Committee A, REC ref. 11/SC/0302), which aimed to examine participant motivations, attitudes and factors influencing participation in human challenge research.

8.19. Definition of End of Trial

The clinical phase of the study has ended when the last participant completes their last visit. The definition of the end of the study is when laboratory analysis has been completed.

9. INTERVENTIONS

9.1. *S.* Typhi challenge strain

GMP manufacture

The *S.* Typhi (Quailes strain) was originally supplied by the University of Maryland and then transferred to the Health Protection Agency (Porton Down, Salisbury, UK) via the University of Oxford, and manufactured to GMP standards. It has been used as a challenge agent for healthy volunteers since 2009 in Oxford. Using the results of the previously performed dose-finding study (OVG 2009/10 REC ref. 10/H0604/53), the required dose will be administered to provide an inoculum of 1-5x10^4 CFU.

A master cell bank (OX-SQ-MCB1) was generated in 2009 at the Oxford site which was then formally transferred to a GMP site. GMP batches were then transported and stored in Oxford under controlled conditions at -80°C. GMP batches have been subjected to quality control and characterisation including Gram stain, antibiotic sensitivity (fully sensitive), purity and content testing.

Two vials of GMP-grade *S.* Typhi Quailes strain were supplied by the Oxford Vaccine Group to the Galan laboratory (Department of Microbial Pathogenesis, Yale School of Medicine, New Haven, CT, USA), who undertook a targeted knock-out of the typhoid-toxin pathogenicity island, generating a variant of the parent strain that is identical save for the absence of the genes encoding the typhoid toxin. Deletion of the toxin-encoding genes was confirmed through phenotypic assays and nucleotide sequencing of the toxin encoding region. Both the wild-type and toxin-negative strains were subsequently supplied to the Walter Reed Army Institute of Research, MD, USA (WRAIR), who undertook manufacture to GMP standard of both strains. Full antibiotic sensitivity and microbial purity of both strains in the master cell banks was demonstrated and further characterisation work including genome sequencing has been completed at the Sanger Institute, UK

Samples will be taken at defined time points to evaluate the stability of the frozen GMP manufactured product. Stability testing will be performed by WRAIR, in accordance to established SOPs. In addition, parallel stability testing will be performed locally at OVG.

Storage

The *S.* Typhi (Quailes strain WT and TN) for inoculation of participants are stored as a frozen suspension in soya tryptone medium containing 10% sucrose. Suspensions have been labelled with the contents (*S*. Typhi Quailes strain), date of manufacture, CFU information, storage conditions, batch and vial number.

Vials of the required concentration of *S.* Typhi (Quailes stain) will be thawed and diluted immediately prior to use.

Accountability for the challenge strain

The investigator will be responsible for adequate and accurate accounting of *S.* Typhi vials prepared for administration to participants. The investigator or designee will administer the study *S.* Typhi vials only to individuals included in this study following the procedures set out in this study protocol and the associated OVG SOPs and Study Plans. The date, dosage and time of administration will be recorded.

The study team will track all vials of *S.* Typhi that have been used, administered to participants and wasted within an accountability log.

9.2. Compliance with Trial Treatment

The challenge agents will be administered by trained doctors and nurses. Administration will be documented according to GCP guidelines and relevant OVG SOPs. Issues related to compliance are therefore the responsibility of the study staff that have received appropriate training.

9.3. Concomitant Medication

Any medication taken by the participant at the time of enrolment into the study or during the first 90 days of the study period will be recorded on the eCRF. Medications as outlined in Sections 8.12 and 8.13 will be provided by study staff according to clinical indication.

9.4. Post-trial Treatment

Study medication will not be continued beyond the study period. Unused medications prescribed during the study will be returned to the study team for disposal 28 days after typhoid challenge.

10. LABORATORY

10.1. Blinding of laboratory samples

Details regarding the process of blinding laboratory samples and the laboratory methods to be used are given in relevant OVG SOPs and Study Plans. Samples processed by departments at the Oxford University Hospitals will be processed using the participant study number and participant initials.

10.2. Bacteriology

Blood culture

After inoculation of aerobic broth with 10 mL of the participant's blood (*BACTEC* PLUS Aerobic/F culture vial; BD, Oxford, UK), culture will be performed using the BACTEC 9240 continuous monitoring system in the microbiology laboratory of the Oxford University Hospitals NHS Trust, according to current SOPs. Identification of organisms cultured will be by biochemical (API, Analytical Profile Index; bioMérieux, Basingstoke, UK) and serological methods, latterly by agglutination with *S.* Typhi anti-sera. Confirmed isolates will be tested for antibiotic susceptibility using standard methods.

Quantitative culture of whole blood will be performed to determine the number of organisms in the blood, using the Wampole™ Isostat® Isolator system (Oxoid Ltd, Basingstoke). Enumeration of *S.* Typhi organisms in the blood will be performed by lysis centrifugation followed by direct plating onto non-selective media.

Stool culture

Stool samples supplied by participants should be delivered to CCVTM within 24 hours of being taken. If possible, the samples should be kept cool until delivered to the CCVTM and then stored at 2°C to 8°C. The time of sampling will be noted on the sample form.

Routine stool cultures and screening for enteric pathogens will be performed by the microbiology laboratory, OUH, according to relevant SOPs. Stool will be inoculated directly onto XLD agar for semi-quantitative culture and into Selenite F enrichment broth for qualitative culture. After overnight incubation (at 37.0°C), each sample will be sub-cultured onto Salmonella-selective chromogenic agar (SALM agar, E&O Laboratories Ltd, Bonnybridge, Scotland). Suspicious colonies will be identified as per the blood culture method described above.

Stool cultures will be taken at Day 0 (challenge), throughout the 14 day post-challenge period and at visits after typhoid diagnosis. Participants will be required to supply further stool samples until proven not to be shedding *S.* Typhi in three consecutive samples.

Isolates of *S*. Typhi may be retained for phage typing or further investigation by the reference laboratory if challenge strain confirmation is required by PHE.

Additionally, quantitative stool cultures or PCR may be performed at the OVGL, to assess the burden of stool shedding. Isolates from stool samples will be stored frozen for future analysis, which may include phage typing or genetic sequencing.

Analysis of the relative composition of bacterial populations within the bowel may be performed on collected stool samples. The faecal microbiome at baseline and its effects on challenge outcome, bacterial dynamics and response to antibiotic treatment may be assayed using techniques including pyrosequencing. After collection, stool will be stabilised in RNA-later and stored at -80°C, prior to further assessment.

Assessment of *Staphylococcus aureus* carriage status

There is increasing evidence that the human microbiome can influence immune responses in health and disease [43]. The human challenge model provides a means to study the interaction between the microbial flora and the human immune system in a controlled setting and to investigate how this relates to the control of infectious diseases. Earlier typhoid challenge studies have focussed on the gastrointestinal microbiome through serial collection of stool samples following challenge or vaccination. In this study we aim to further investigate how the human microbiome influences and interacts with the clinical and immunological response to *S.* Typhi challenge, with a particular focus on nasopharyngeal microbiota.

An association between *Staphylococcus aureus (S. aureus)* carriage and IL-17A responses has been demonstrated in a group of healthy adult volunteers (Y. Yamaguchi, D. Wyllie - unpublished observations). IL-17A is a pro-inflammatory cytokine thought to play a key role in mucosal immunity and protection against intracellular pathogens [44]. Previous studies have hypothesised that IL-17 producing T lymphocytes may play a role in protection against *S.* Typhi infection. For example, vaccination with live attenuated *S.* Typhi Ty21a induces IL-17A producing CD8+ T-cells [45] and IL-17 producing T-lymphocytes have been associated with protection against invasive *Salmonella* disease [46 47]. In order to further explore the association of *S. aureus* carriage with IL-17 responses we will establish the *S. aureus* carriage status in all study participants. Carriage status will be correlated with the response to *S.* Typhi challenge, including the development of typhoid fever, the development of bacteraemia, monocyte/lymphocyte ratio, lymphocyte immunophenotype and kinetics of cytokine production (see below).

We will identify persistent nasal carriers of *Staphylococcus aureus* (*S. aureus*) by collecting swabs of the anterior nares and saliva samples at three time points (screening, pre-challenge visit (D-7), baseline (DO) visits). Quantitative and qualitative culture will be performed on these samples. Analysis of the nasal, salivary and gut microbiome may also be performed using 16s rRNA sequencing. Persistent carriage is

defined as the isolation of *S. aureus* from at least two samples taken at least one week apart and is estimated that up to 30% of the healthy adult population [48]. Analysis of the microbiome may also be performed using 16s rRNA sequencing.

10.3. Molecular diagnostics

Blood PCR detection

The Oxford Vaccine Group has developed a fast and highly sensitive novel TSB-bile blood culture-PCR assay which has been used to detect low levels of *S.* Typhi in the blood of participants after challenge in previous studies (OVG 2009/10, OVG 2011/02). Whole blood (5ml) is inoculated into 20ml tryptone soya broth containing 3.0% ox bile and micrococcal nuclease. The blood/broth culture is shaken at 220rpm in a 37°C incubator for 5 hours, and then centrifuged at 5,000rpm for 20 minutes. The supernatant is then discarded and the pellet used for DNA isolation. Isolated DNA will be used as a template for organism detection by PCR amplification, utilising the *S.* Typhi fliC-d gene employing primers H-for (ACTCAGGCTTCCCGTAACGC) and Hd-rev (GGCTAGTATTGTCCTTATCGG).

We will continue developing novel PCR/RT-PCR methods to shorten the turnaround time without any compromise to specificity and sensitivity. We will use either the present TSB-bile blood culture-PCR assay or a newly developed PCR/RT-PCR method to detect low levels of *S.* Typhi in the blood of participants after challenge in the current study and attempt to analyse bacterial gene expression profiles during acute typhoid infection.

Novel molecular diagnostic techniques may also be assessed with study collaborators, including the detection of *S.* Typhi cell-free DNA (cfDNA) in urine and blood specimens. These tests may be performed in a sub-set of study participants depending on reagent availability. Briefly, blood and urine samples will be collected and/or processed in tubes containing cfDNA preservative, as outlined in the laboratory analysis plan.

10.4. Inflammatory responses

Plasma cytokines

The kinetics of the inflammatory response will be measured in stored plasma samples. A custom commercial multiplex bead-array kit is in use in the laboratory (including, but not limited to, IL-1β, IL-2, IL-6, IL-8, IL-10, IL-12, IL-17A, TNF-α and IFN-γ.). Plasma samples will be isolated from heparinised blood and stored at -80°C until assays are performed.

10.5. Antibody responses

Serum

Serum will be isolated from blood and stored at -80°C prior to assays being performed. *S*. Typhi-specific antibody responses to multiple antigens including typhoid toxin components, Vi, H and O-antigens will be tested at baseline and various time points post-challenge. Antibody responses will be assessed using a combination of commercial kits and in-house developed ELISAs. Functional antibody responses may also be determined using serum bactericidal or opsonophagocytic assays.

B-cell responses

Antibody-secreting cells (ASCs) that secrete antibodies against *S.* Typhi specific antigens including toxin components, Vi, O and H will be measured by using ELISPOT [49]. Briefly, peripheral blood mononuclear cells (PBMCs) will be separated by density gradient centrifugation and added to antigen-coated ELISPOT plates. In the ELISPOT, antigen specific antibody secreted by individual ASCs will be quantified. Memory B-cells responses will be detected after *in vitro* polyclonal stimulation.

10.6. Cellular immune responses

Cellular immune responses will be analysed using PBMCs isolated using density gradient centrifugation. Assays performed may include activation, proliferation, and cytokine and surface marker measurements using techniques such as intracellular staining and multi-chromatic flow cytometry of cells. Additional analysis may be performed using heavy metal ion tags (CyTOF) to increase the number of parameters being investigated.

10.7. Factors affecting susceptibility and response to infection

DNA analysis

Analysis of DNA sequencing or the association with single-nucleotide polymorphisms (SNPs) and epigenetics will be performed using DNA extracted from clotted blood, derived from collected serum samples. With consent, these samples will be used to identify host-susceptibility factors for susceptibility and response to challenge using a candidate gene approach. Samples will be taken at baseline and at post-challenge time points to assess for epigenetic changes in DNA transcription.

Functional genomics

In brief, blood will be collected in Tempus™ Blood RNA Tubes and stored at -80°C until processing. RNA will be extracted and used for study of gene expression profiles. Gene expression profiles will be determined using gene expression microarrays and/or mRNA-seq technologies at baseline and post-challenge time points.

10.8. Detection of typhoid toxin activity

Mass spectrometry

We will develop mass spectrometry based assays to directly detect typhoid toxin following challenge, using samples of serum, plasma and/or urine collected at baseline various time points after challenge. Urine samples will be collected and stored on ice prior to transfer to the OVG Laboratory. Samples will be centrifuged for 10 minutes at 13000g (to remove cellular debris) and subsequently stored at -20°C overnight, prior to transfer to -80°C. Following collection, samples will be stored and analysed in conjunction with study collaborators (Galán laboratory (Department of Microbial Pathogenesis, Yale School of Medicine) using techniques including Selected Reaction Monitoring mass spectrometry (SRM).

Peripheral signatures of typhoid toxin activity

PBMCs, serum, plasma and/or urine will be collected from participants at baseline and various time points after challenge for metabolomics and proteomic analysis, to compare difference in peripheral signatures following challenge with wither WT or TN strains of *S.* Typhi.

10.9. Other laboratory investigations

All other laboratory tests including FBC, WBC differential counts, C-reactive protein, urea, creatinine, electrolytes, aspartate transaminase (AST), alkaline phosphatase (ALP), alanine transaminase (ALT), bilirubin, amylase will be performed using the OUH, NHS laboratories. Blood samples will be collected in assay sample tubes and delivered to OUH clinical laboratories for analysis according to national SOPs.

Samples collected as part of this study may also be used for other exploratory studies of scientific relevance by the OVG laboratory or any of the collaborating laboratories. These samples may include stool, serum, extracted DNA and RNA, and PBMCs. Frozen samples will be stored under the ethical approval for this study until study completion. At this time, samples will be transferred to the Oxford Vaccine Centre Biobank subject to participant consent (see Section 8.3). Studies may include further investigation of the inflammatory and immunological response to vaccination and challenge.

Blood samples obtained from healthy consenting adult volunteers may be used as controls for laboratory assay development. Buffy coats will be obtained from the National Blood Transfusion Service (UK) for use of the PBMCs in the optimisation, validation and control of ELISPOTs and cell phenotyping assays.

11. SAFETY REPORTING

According to current recommendations from the Medicines and Healthcare products Regulatory Agency, challenge agents do not constitute Investigational Medicinal Products or 'treatment'.

11.1. Definitions (AE)

Adverse Event (AE)	Any untoward medical occurrence in a participant to whom a medicinal product has been administered, including occurrences which are not necessarily caused by or related to that product.<br><br>An AE can therefore be any unfavourable and unintended sign (including an abnormal laboratory finding), symptom or disease temporally associated with the ingestion of *S.* Typhi, whether or not considered related to this.
Adverse Reaction (AR)	All untoward and unintended responses to *S*. Typhi challenge or any study procedure.<br><br>The phrases "responses to *S.* Typhi" means that a causal relationship between *S.* Typhi and an AE, is at least a reasonable possibility, i.e. the relationship cannot be ruled out.<br><br>All cases judged by either the reporting medically qualified professional or the Sponsor as having a reasonable suspected causal relationship to *S.* Typhi challenge qualify as adverse reactions.<br><br>For the avoidance of doubt, if the reaction to *S*. Typhi challenge is within a defined set of criteria as listed under 'Expected Adverse Events' (see section 11.2), which may be expected in an individual developing symptomatic typhoid fever, these will be classified as outcomes and not as adverse events.<br><br>Adverse events may be identified if the participant is specifically asked to record their presence/absence/severity in the e-diary or if the participant volunteers these spontaneously. A list of expected adverse events is shown in **Table 8**.
Serious Adverse Event (SAE)	A serious adverse event is an AE that results in any of the following outcomes, whether or not considered related to the study medication or *S*. Typhi ingestion:<br><br>• Death,<br>• is life-threatening,<br>• requires inpatient hospitalisation or prolongation of existing hospitalisation,<br>• results in persistent or significant disability/incapacity,<br>• consists of a congenital anomaly or birth defect.

	Other 'important medical events' may also be considered serious if they jeopardise the participant or require an intervention to prevent one of the above consequences.<br>NOTE: The term "life-threatening" in the definition of "serious" refers to an event in which the participant was at risk of death at the time of the event; it does not refer to an event which hypothetically might have caused death if it were more severe.
Serious Adverse Reaction (SAR)	An adverse event (expected or unexpected) that is both serious and, in the opinion of the reporting investigator, believed with reasonable probability to be due to one of the study treatment, based on the information provided. (See Causality Assessment, section 11.3).
Suspected Unexpected Serious Adverse Reaction (SUSAR)	A serious adverse reaction, the nature or severity of which is not consistent with the expected outcomes following challenge with *S.* Typhi.

NB: To avoid confusion or misunderstanding of the difference between the terms "serious" and "severe", the following note of clarification is provided: "Severe" is often used to describe intensity of a specific event, which may be of relatively minor medical significance. "Seriousness" is the regulatory definition supplied above.

Any pregnancy occurring during the clinical trial and the outcome of the pregnancy should be recorded and followed up for congenital abnormality or birth defect, at which point it would fall within the definition of "serious".

11.2. Definition of an Expected Adverse Event

Any expected adverse event related to symptomatic typhoid infection (as specified in **Table 8**) will be deemed an 'Expected Adverse Event' and will be documented in the eCRF, however, not in the 'Adverse Event' section of the eCRF. These symptoms will be considered 'Expected Adverse Events' if they occur between Day 3 and Day 14 post-challenge (inclusive) or between Day 3 and TD +96 hours. Expected events can be recorded as adverse events, at the discretion of study staff, if they are believed to be unusual (for example, in severity, frequency, duration or character).

Expected Events – Typhoid Infection
**Headache**
**Fever >37.5°C but <40.0°C (fever ≥40.0°C will be considered an AE)**
**Generally unwell**
**Loss of appetite**
**Rash**
**Constipation**
**Diarrhoea**
**Abdominal pain**
**Myalgia or arthralgia**
**Cough**
**Nausea or vomiting**

Table 8. Events which will be attributed to development of symptomatic enteric fever

Additionally, symptoms occurring outside of the specified time frame will also be considered adverse events.

11.3. Causality

Causality assessment will follow the guidelines from existing OVC SOPs, with the exception of causality attribution and classification that will be based on the following criteria.

No relationship

- No temporal relationship to vaccine administration or *S*. Typhi ingestion, *and*
- alternative aetiology (clinical, environmental or other intervention), *and*

- does not follow pattern of recognised response to typhoid infection.

Possible

- Reasonable temporal relationship to *S*. Typhi ingestion, *or*
- event not readily explained by alternative aetiology (clinical, environmental or other interventions), *or*
- similar pattern of response to that seen to typhoid infection.

Probable

- Reasonable temporal relationship to *S*. Typhi ingestion, *and*
- event not readily produced by alternative aetiology (clinical, environment, or other interventions), *or*
- known pattern of response with typhoid infection.

Definite

- Reasonable temporal relationship to *S.* Typhi ingestion, *and*
- event not readily produced by alternative aetiology (clinical, environment, or other interventions), *and*
- known pattern of response to typhoid infection.

11.4. Adverse Events of Special Interest (AESI)

The following events will be considered AESIs and reported to the DSMC in the same manner as for reporting SAEs:

- Severe typhoid infection (as defined in section 8.10, **Table 4**).

  Complications such as perforation or haemorrhage occur almost exclusively in patients who are untreated for an extended period. Participants in this study will be treated 12 hours after developing fever or if *S*. Typhi is recovered from a blood culture drawn 72 hours post-challenge. They will be closely monitored during the initial study phase, until a 14-day course of antibiotic is completed, minimising the risk of complications.
- Failure to clinically or bacteriologically cure a participant of typhoid infection within 14 days of antibiotic therapy, progression to chronic carrier state, or relapse.
- Transmission of *S*. Typhi to a contact of a participant (See section 8.15).

- AEs requiring a physician visit or Emergency Department visit which, in the opinion of study staff, are related to the challenge with *S.* Typhi.
- Pregnancy.

  The possible adverse effects of *S.* Typhi infection or the effect of some antibiotics on the outcome of pregnancy are unknown. Therefore, pregnant women will be excluded by history and laboratory tests, and female participants will be specifically instructed to prevent conception during the challenge period of the study until completion of antibiotic therapy and clearance of typhoid infection is confirmed. Should pregnancy occur, information about outcome of the pregnancy will be sought.

11.5. Expected Serious Adverse Events and Serious Adverse Reactions

No serious adverse events or reactions are expected. This is addressed in Adverse Events of Special Interest (section 11.4).

11.6. Procedures for recording Adverse Events

In the 21 days after challenge, solicited symptoms will be recorded once daily by the participant in the e-Diary. All Aes occurring during this time, either observed by study staff or reported by the participant, irrespective of their relatedness to the study medication, will be recorded on the eCRF and graded according to criteria defined in Appendix A, B and C. In addition participants will be requested to continue to record any unsolicited symptoms until 28 days after challenge in the e-diary.

Aes will be recorded using the following guidance:

- All Aes occurring for 21 days post-challenge, either observed by study staff or reported by the participant, whether or not attributed to study medication, will be recorded in the eCRF. Events starting within these time periods but persisting beyond the above specified time points will similarly be recorded in the eCRF.
- From Day 22 to Day 90, medically significant adverse events (e.g. requiring medical review by a physician or Emergency Department visit) will be recorded in the eCRF, whether or not these are attributed to *S.* Typhi ingestion or study medication.
- Pre-existing medical conditions (present prior to enrolment into the study) are considered “concurrent medical conditions” and should not be recorded as Aes. However, if the participant experiences a worsening or complication of the condition, the worsening or complication should

be recorded as an AE. Study staff will ensure that the AE term recorded captures the change in the condition (e.g., “worsening of”).

- Each AE should be recorded to represent a single diagnosis. Accompanying signs or symptoms (including abnormal laboratory values) should not be recorded as additional Aes.
- Changes in laboratory values are only considered to be Aes if they are judged to be clinically significant, e.g., if some action or intervention is required. If abnormal laboratory values are the result of pathology for which there is an overall diagnosis, the diagnosis only should be reported as one AE.
- The following information will be recorded in the eCRF: description of the AE, the date of onset and end date (if there is no end date, it will be stated if the AE it is ongoing or resolved with sequelae), severity, assessment of relatedness to medication or challenge with *S.* Typhi (as judged by a medically qualified doctor) and the action taken. Follow-up information should be provided as necessary.

- Aes considered related to study procedures, medications or *S.* Typhi challenge, as judged by a medically qualified doctor will be followed either until resolution, or the event is considered stable.
- All Aes that result in a participant’s withdrawal from the study or that are present at the end of the 28 day challenge period, will be followed up until a satisfactory resolution occurs or until a non-study related causality is assigned. It will be left to the investigator’s clinical judgment whether or not an AE is of sufficient severity to require the participant’s removal from the study. A participant may also voluntarily withdraw from the study due to what he or she perceives as an intolerable AE. If either of these occurs, the participant must undergo an end of study assessment. With participant consent, follow-up will be arranged with their GP for appropriate care until symptoms cease or the condition becomes stable.

11.7. Procedures for reporting Serious Adverse Events

All serious adverse events (SAE) must be recorded on the Sponsor’s SAE form and reported by fax and email to the Sponsor and DSMC Chair within 24 hours of discovery or notification of the event. Additional information received for a case (follow-up or corrections to the original case) need to be detailed on a new SAE form and faxed or emailed to the Sponsor and DSMC Chair, as above. All SAEs will also be reported to the CTRG study monitor.

The chair of the DSMC will perform an independent review of SAEs and request any further information required in a manner adherent to the procedures and timelines of the DSMC Charter. Documentation of this review will be kept in the TMF.

11.8. SUSAR Reporting

All SUSARs will be reported by the Chief Investigator to the Sponsor, MHRA and to the REC and other parties as applicable. For fatal and life-threatening SUSARS, this will be done no later than 7 calendar days after the Sponsor or delegate is first aware of the reaction. Any additional relevant information will be reported within 8 calendar days of the initial report. All other SUSARs will be reported within 15 calendar days.

Treatment codes will be unblinded for specific participants.

Principal Investigators will be informed of all SUSARs for the relevant IMP for all studies with the same Sponsor, whether or not the event occurred in the current trial.

For all deaths, available autopsy reports and relevant medical reports will be made available for reporting to the relevant authorities.

11.9. Annual Progress report (APR)

An Annual Progress Report (APR) of the trial will be submitted once a year by the Chief Investigator to the Research Ethics Committee and the Sponsor. The APR will be submitted within 30 days the anniversary date of the favourable opinion of that REC.

11.10. Safety Profile Review

A sentinel group of two participants will be challenged with either one of the WT or TN strains. The sentinel group will be randomised using a block size of two. If there are no safety concerns (as assessed by a study doctor and discussed with the senior investigator and/or clinician as appropriate) by Day 14 post-challenge, the remaining participants will be challenged. Randomisation using varying block sizes will occur thereafter. The DSMC chair and/or other members of the DSMC committee will be updated at regular time points following the initial challenge safety profile will be reviewed after the first 10 participants have been challenged and upon completion of all participants reaching Day 28. Any concerns will be referred to the DSMC Chair.

11.11. Trial Management Committee

The trial investigators will form the trial management committee and will provide on-going management of the trial.

11.12. Data Safety Monitoring Committee

A Data and Safety Monitoring Committee (DSMC) will be appointed to provide real-time oversight of safety and trial conduct.

The DSMC is independent and will review unblinded safety data throughout the study according to the DSMC Charter. The DSMC will have access to data and, if required, will monitor these data and make recommendations to the study investigators on whether there are any ethical or safety reasons why the trial should not continue and will particularly review the control group attack rate to confirm the challenge model is proceeding as expected. A summary of all AESIs and SAEs to date will be provided to the DSMC on request. The DSMC will also be notified if the study team have any concerns regarding the safety of a participant or the general public (e.g. if a participant is not contactable after *S*. Typhi challenge and potentially infectious to others).

The outcome of each DSMC review will be communicated directly to the study investigators and documentation of all reviews will be kept in the TMF. The Chair of the DSMC will also be contacted for advice when the Chief Investigator feels independent advice or review is required.

11.13. Procedure to be followed in the event of an abnormal finding

Abnormal clinical findings from medical history, examination or blood tests, will be assessed as to their clinical significance using the severity grading criteria for Adverse Events tables (see Appendix A, B, C). If a test result is deemed clinically significant, it may be repeated, to ensure it is not a single occurrence. If a test remains clinically significant, the participant will be informed and appropriate medical care will be arranged with the permission of the participant. Decisions to exclude potential participants from enrolling in the trial or to withdraw a participant from the trial will be at the discretion of the study team.

11.14. Staff and Investigator safety

All staff working on the project will be required to follow strict infection control techniques as outlined in local OVG SOPs. All staff members working at the CCVTM will be informed of the commencement of the challenge study. All staff members associated with the study will be offered a typhoid vaccine through occupational health and safety.

12. STATISTICS

12.1. Description of Statistical Methods

The analyses for the primary endpoint will be descriptive in nature and will not include formal hypothesis testing.

Binary outcomes for the primary endpoint will be described using percentages and 95% binomial exact (Clopper-Pearson) confidence intervals.

Time-to-event endpoints analyses will be conducted using the Kaplan-Meier method and presented as Kaplan-Meier plots.

Immunogenicity data are expected to be skewed and so will be log transformed prior to analysis. Results will be presented as geometric means with 95% confidence intervals. Values below the limit of detection will be replaced by half the value of the lower limit.

Data on reasons for study exclusion will be descriptive in nature only and will not include formal hypothesis testing.

12.2. The Number of Participants

A total of 40 participants will be randomised 1:1 to receive challenge with either wild type *Salmonella* Typhi Quailes strain (WT) or a typhoid toxin-deficient isogenic mutant of *S.* Typhi Quailes strain SB6000 (TN). It is anticipated that the attack rate following challenge with the TN strain will be reduced compared to the WT strain. Assuming an attack rate of 65% following WT strain (as observed in previous studies) and 50% attack rate following TN strain, and accounting for 10% drop out, 20 participants in each group will lead to 95% confidence intervals for attack rate of 41% to 85% in the group randomised to WT strain and 27% to 73% in the group randomised to TN strain. Table 9 displays the confidence intervals for observed attack rates of 0 – 70% using a sample size of 20 in each group.

Confidence Level	Sample Size (N)	Proportion diagnosed (P)	Lower Limit	Upper Limit	Actual Width	Width if P = 0.5
0.95	20	0	0	0.168	0.168	0.456
0.95	20	0.3	0.119	0.543	0.424	0.456
0.95	20	0.35	0.154	0.592	0.438	0.456
0.95	20	0.4	0.191	0.639	0.448	0.456
0.95	20	0.45	0.231	0.685	0.454	0.456
0.95	20	0.5	0.272	0.728	0.456	0.456
0.95	20	0.55	0.315	0.769	0.454	0.456
0.95	20	0.6	0.361	0.809	0.448	0.456
0.95	20	0.65	0.408	0.846	0.438	0.456
0.95	20	0.7	0.457	0.881	0.424	0.456

Table 9. Confidence intervals for observed attack rates using sample size 20 in each group

12.3. Analysis of demographic and baseline characteristics

Descriptive statistics relating to participant characteristics at baseline will be calculated overall and by group. No formal statistical comparisons of baseline characteristics between randomised groups will be conducted.

12.4. Analysis of the primary endpoint

The analysis of the primary endpoint will be descriptive only. The percentage of participants in each group, who meet the criteria for diagnosis of typhoid fever, will be calculated using a 95% Clopper-Pearson Exact confidence interval. The numerator will be the number of participants who meet the criteria for diagnosis and the denominator will include all participants excluding those who withdrew or were treated prior to Day 14 without being diagnosed.

A secondary analysis of the primary endpoint will be conducted using the Kaplan-Meier method which will include all participants. Participants who withdrew or were treated prior to Day 14 and had no diagnosis of enteric fever will be censored in the analysis at the time of withdrawal or treatment. Participants who were not diagnosed with enteric fever and were treated at Day 14 will be counted as 'events' occurring at Day 14 in the analysis under the assumption that enteric fever would have occurred had continual monitoring without treatment been possible during the unobserved time period beyond Day 14.

Time-to-event analyses of individual components of the primary outcome (e.g. positive blood culture, oral temperature ≥ 38.0°C etc.) will be conducted using the Kaplan-Meier method and will include all

participants. Participants not meeting the criteria for an individual component of the primary endpoint will be censored in the analysis at the time of diagnosis or at Day 14 for those undiagnosed.

12.5. Definitions of secondary clinical and microbiological end-points

- Time to onset of symptoms – Time (Days) to first recorded solicited symptoms in the diary card OR first temperature ≥38°C, excluding the first 48 hours following challenge.
- Duration of illness – Time (Days) from first recorded individual solicited symptoms to complete resolution of individual recorded symptoms in the diary card, excluding the first 48 hours following challenge.
- Fever clearance time – Time (Hours/Days) from first dose of treatment until temperature ≤37.5°C for a 48 hour period.
- Symptom severity
    a. The proportion of participants with maximum symptom severity score graded as mild, moderate or severe following challenge.
    b. The proportion of participants meeting the criteria for severe enteric fever (**Table 4)**.
    c. The proportion of participants reporting the typhoid symptom triad (fever, headache and abdominal pain).
    d. The proportion of participants recording one or more severe solicited symptoms following challenge.
    e. Total symptom scores calculated by summing numerical values assigned to the severity of all solicited symptoms between Day 0 to Day 14 (0=not present; 1=mild; 2=moderate; 3=severe).
    f. Individual symptom severity scores calculated by summing numerical values assigned to the severity of individual solicited symptoms between Day 0 to Day 14 (0=not present; 1=mild; 2=moderate; 3=severe).
- Time to diagnosis – Time (Hours/Days) from challenge until fulfilment of diagnostic criteria (taken as date/time Gram negative rods are detected in blood culture AND/OR recorded temperature ≥38°C for 12hours)
- Time to onset of bacteraemia – Time (Hours/Days) from challenge until the date/time first positive blood culture collected.
- Duration of bacteraemia – Time (Hours/Days) from collection of first positive blood culture until date/time of the first negative blood culture and blood cultures are persistently negative.
- Quantification of bacteraemia at time of diagnosis – Concentration of bacteria (CFU/ml) in 10ml blood taken at the time of diagnosis using the Wampole™ Isostat® Isolator system (Oxoid Ltd, Basingstoke).
- Time to onset of stool shedding – Time from challenge (Hours/Days) to the first positive stool culture.
- Duration of stool shedding – Cumulative number of days where positive stool culture for *Salmonella* Typhi collected
- Pattern of stool shedding – Descriptive
- Persistent *S. aureus* carriage – The isolation of *S. aureus* from at least two samples taken at least one week apart.
- Haematological and biochemical parameters

a. Total Haemoglobin (g/L)
b. Haemoglobin change from baseline (Hb g/l D0 – Hb g/l D14)
c. Total White Cell Count ($x10^9$/l)
d. Platelet counts ($x10^9$/l)
e. Neutrophil count ($x10^9$/l)
f. Lymphocyte count ($x10^9$/l)
g. Monocyte count ($x10^9$/l)
h. Eosinophil count ($x10^9$/l)
i. Monocyte/Lymphocyte ratio
j. Urea & Electrolytes (Na, K+, Urea, Creatinine –mmol/l)
k. C-reactive protein (mg/l)
l. Liver function tests (Bilirubin [umol/l], aspartate transaminase (AST IU/l), alkaline phosphatase (ALP IU/l), alanine transaminase (ALT IU/l), Albumin (g/L)

12.6. The Level of Statistical Significance

The main study outcome is a proportion, which will be quoted with 95% confidence intervals. 95% confidence intervals will be used throughout.

12.7. Criteria for the Termination of the Trial

The Chief Investigator with the Data and Safety Monitoring Committee will have the right to terminate the study at any time on grounds of participant safety. If the study is prematurely terminated the clinical study team will promptly inform the participants and will ensure appropriate therapy and follow-up. If the study is halted, the Sponsor, Oxford University Hospitals NHS Trust, MHRA and relevant Ethics Committee will be notified within 15 days of this occurring.

12.8. Procedure for Accounting for Missing, Unused, and Spurious Data.

All available data will be used in the analyses and there will be no imputations for missing data. Participants will be analysed according to the type of challenge received for summaries by group.

Participants will be replaced if the diagnosis of typhoid has not been reached by the time of withdrawal. Similarly, additional participants will replace participants who require treatment before the formal diagnosis of typhoid or Day 14. Participants withdrawn after this time period (i.e. after being diagnosed with typhoid infection, or after 14 days post-challenge) may be replaced at the discretion of the Chief Investigator.

12.9. Inclusion in Analysis

All participants will be included in analyses if they are successfully challenged on Day 0 and have at least one post- challenge assessment. If a participant later withdraws from the study, data up until that point will be included in the analysis. Participants who are treated or withdraw before meeting the primary endpoint at day 14 and have no diagnosis of enteric fever prior to day 14 will not be included in the primary analysis of the primary endpoint.

All participants will be included in time-to-event analyses.

If a participant is inadvertently given an incorrect dose or challenge product they will be analysed according to the dose they received rather than the planned dose.

12.10. Procedures for Reporting any Deviation(s) from the Original Statistical Plan

Any additional analysis or deviations from the analysis plan will be documented and updated according to the statistical standard operating procedure.

13. DATA MANAGEMENT

13.1. Access to Data

Direct access will be granted to authorised representatives from the Sponsor and host institution and the regulatory authorities to permit trial-related monitoring, audits and inspections. The BMGF will have access to raw, anonymised data on request.

13.2. Source Data

Source documents are original documents, data, and records from which some participants' electronic data is directly populated. These include, but are not limited to, hospital records (from which medical history and previous and concurrent medication may be summarised into the electronic data capture database), clinical and office charts, laboratory and pharmacy records, diaries, microfiches, radiographs, and correspondence. In this study, electronic data entries will be considered source data when it is the site of the original recording. All documents will be stored safely under strict confidentiality and with restricted access. The participant will be referred to by the study participant number/code on study-specific documents, other than the signed consent forms and the participant contact sheet. Participant details populated from the electronic database are kept in the form of an electronic participant and screening log located on a password protected OVG network drive.

13.3. Data Recording and Record Keeping

The study team will populate the content of participants' CRFs, which will be in a paper and/or electronic format, using an OpenClinica™ database. This database is stored on a secure server within the UK and has restricted access and is password-protected with accountability records. This data includes safety data, laboratory data (both clinical and immunological) and outcome data. All information transcribed to and from the OpenClinica™ database is by encrypted (Https) transfer.

Each study participant will have a unique screening number, which will be allocated following phone screening. A participant number is allocated at the time of enrolment. After enrolment, participants will be identified by a study specific participant number and/or code. Samples sent to laboratories for processing will be identified by, participant number, randomised laboratory number and participant initials.

Anonymised CRFs and any other documents related to primary and secondary endpoints of the study will also be transmitted to the Sponsor at completion of the study.

13.4. Data integrity

Data collection and storage will be inspected throughout the study by internal (performed by the Oxford Vaccine Group) and external (by the study Sponsor) monitoring.

13.5. Data archiving and storage

Following completion of the study, all personal data will be transmitted to the Sponsor with an anonymised key and will also be kept for a period of 25 years by Ardington Archives storage (Faringdon, Oxford) according to the relevant OVG and OVC SOPs. Storage of this data will be reviewed every 5 years and files will be confidentially destroyed if storage is no longer required. Professor Andrew J. Pollard, or his successor, as Head of the Oxford Vaccine Group will have the responsibility for custody of the data.

14. QUALITY ASSURANCE PROCEDURES

14.1. Investigator procedures

The study will be conducted in accordance with the current approved protocol, GCP, relevant regulations and standard operating procedures. Approved and relevant Standard Operating Procedures (SOPs) and Laboratory and Clinical Study Plans will be used at all clinical and laboratory sites.

14.2. Monitoring

Monitoring will be performed according to Good Clinical Practice (GCP) by CTRG or parties appointed by the Sponsor. Following written SOPs, the monitors will verify that the clinical trial is conducted and data are generated, documented and reported in compliance with the protocol, GCP and the applicable regulatory requirements. The investigator sites will provide direct access to all trial related source data/documents and reports for the purpose of monitoring and auditing by the Sponsor and inspection by local and regulatory authorities.

14.3. Direct access to source data/documents

Direct access will be granted to authorised representatives from the Sponsor, host institution and the regulatory authorities to permit trial-related monitoring, audits and inspections.

14.4. Modification to protocol

No amendments to this protocol will be made without consultation with and agreement from the Sponsor. Any amendments to the study that appear necessary during the course of the study must be discussed with the Investigator and Sponsor concurrently. If agreement is reached concerning the need for an amendment, it will be produced in writing by the Chief Investigator and will be made a formal part of the protocol following ethical and regulatory approval.

An administrative change to the protocol is one that modifies administrative and logistical aspects of a protocol but does not affect the participants' safety, the objectives of the study and its progress. An administrative change does not require HRA REC approval.

The Investigator is responsible for ensuring that changes to an approved study, during the period for which NHS REC approval has already been given, are not initiated without NHS REC review and approval except to eliminate apparent immediate hazards to the participant.

14.5. Protocol deviation

Any deviations will be documented in a protocol deviation form and filed in the TMF.

14.6. Audit and Inspection

The Quality Assurance manager operates an internal audit program to ensure that the systems used to conduct clinical research are present, functional, and enable research to be conducted in accordance with study protocols and regulatory requirements. Audits include laboratory activities covering sample receipt, processing and storage and assay validation. The internal audits will supplement the external monitoring process and will review processes not covered by the external monitor.

The Sponsor may carry out audits to ensure compliance with the protocol, GCP and appropriate regulations.

14.7. Trial Progress

The progress of the study will be overseen by the Chief Investigator.

15. ETHICAL AND REGULATORY CONSIDERATIONS

15.1. Declaration of Helsinki

The Investigator will ensure that this study is conducted in accordance with the principles of the current version of the Declaration of Helsinki.

15.2. Guidelines for Good Clinical Practice

The Investigator will ensure that this study is conducted in accordance with relevant regulations and with Good Clinical Practice.

15.3. Approvals

The protocol, informed consent form, participant information sheet and any proposed advertising material will be submitted to an appropriate Research Ethics Committee (REC), and host institution(s) for written approval.

The Investigator will submit and, where necessary, obtain approval from the above parties for all substantial amendments to the original approved documents.

15.4. Reporting

The CI shall submit once a year throughout the clinical trial, or on request, an Annual Progress Report to the REC, host organisation and Sponsor. In addition, an End of Trial notification and final report will be submitted to the REC, host organisation and Sponsor.

15.5. Participant confidentiality

The study staff will ensure that participants' anonymity is maintained. Study participants will be identified by initials and a participant ID number on the CRF. Any electronic databases and documents with participant identifying details will be stored securely and will only be accessible by study staff and authorised personnel. The study will comply with the Data Protection Act, which requires data to be anonymised as soon as it is practical to do so.

15.6. Participant reimbursement

All participants will be reimbursed for their time, travel and for inconvenience based on the following figures:

- Travel expenses £15 per visit (total for 28 visits £420)

- Inconvenience of blood tests: £10 per blood donation (total for 27 blood tests £270)
- Time required for visit: £20 per visit (total for 28 visits £560)
- Time off work compensation: £150 per day for 10 days (total £1500).

Participants will receive a total of £2,750 if they remain in the study for the entire period. Payments will be made via bank transfer. Participants will be required to provide banking details including account name, sort code and account number. All personal banking details will be stored confidentially and retained by OVG while the participant is actively involved in the study. Consent will be obtained prior to requesting and storing personal bank account details.

Participant payments will be requested at the following visits: Screening, Day 14 (or TD+96), Day 28, Day 90, Day 180, and Day 365.

Due to the generous reimbursement for scheduled visits, participants will not be given extra reimbursement for unscheduled visits.

15.7. Other Ethical Considerations

There are no other ethical considerations for this study protocol.

16. FINANCE AND INSURANCE

16.1. Funding

Funding for the study has been provided by the Bill and Melinda Gates Foundation.

16.2. Insurance

The University has a specialist insurance policy in place, which would operate in the event of any participant suffering harm as a result of their involvement in the research (Newline Underwriting Management Ltd, at Lloyd's of London). NHS indemnity operates in respect of the clinical treatment that is provided.

17. PUBLICATION POLICY

The Chief Investigator will co-ordinate dissemination of data from this study. All publications (e.g., manuscripts, abstracts, oral/slide presentations, book chapters) based on this study will be reviewed by each sub-investigator and by the Sponsor prior to submission. All communication or publications concerning the project, including at a conference or seminar, shall acknowledge the Parties and BMGF financial contribution.

18. REFERENCES

1. Crump, J. a, Luby, S. P. & Mintz, E. D. The global burden of typhoid fever. *Bull. World Health Organ.* **82,** 346–53 (2004).

2. Bhan, M. K., Bahl, R. & Bhatnagar, S. Typhoid and paratyphoid fever. *Lancet* **366,** 749–762 (2005).

3. Simanjuntak, C. H. *et al.* Oral immunisation against typhoid fever in Indonesia with Ty21a vaccine. **338,** (1991).

4. Sinha, A. *et al.* Typhoid fever in children aged less than 5 years. *Lancet (London, England)* **354,** 734–7 (1999).

5. Ackers, M. L., Puhr, N. D., Tauxe, R. V & Mintz, E. D. Laboratory-based surveillance of Salmonella serotype Typhi infections in the United States: antimicrobial resistance on the rise. *JAMA* **283,** 2668–73

6. Steinberg, E. B. *et al.* Typhoid fever in travelers: who should be targeted for prevention? *Clin Infect Dis* **39,** 186–191 (2004).

7. Reller, M. E. *et al.* Sexual transmission of typhoid fever: a multistate outbreak among men who have sex with men. *Clin. Infect. Dis.* **37,** 141–4 (2003).

8. Dongol, S. *et al.* The microbiological and clinical characteristics of invasive salmonella in gallbladders from cholecystectomy patients in kathmandu, Nepal. *PLoS One* **7,** e47342 (2012).

9. Gonzalez-Escobedo, G. & Gunn, J. S. Gallbladder epithelium as a niche for chronic Salmonella carriage. *Infect. Immun.* **81,** 2920–30 (2013).

10. Balasegaram, S. *et al.* Guidelines for the public health management of typhoid and paratyphoid in England: practice guidelines from the National Typhoid and Paratyphoid Reference Group. *J. Infect.* **65,** 197–213 (2012).

11. Addiman, S. *et al.* Public health management of Salmonella Typhi/Paratyphi case and contact screening: lessons from North London. *Public Health* **127,** 207–13 (2013).

12. Wong, V. K. *et al.* Phylogeographical analysis of the dominant multidrug-resistant H58 clade of Salmonella Typhi identifies inter- and intracontinental transmission events. *Nat. Genet.* (2015). doi:10.1038/ng.3281

13. Parry, C. M. *et al.* The influence of reduced susceptibility to fluoroquinolones in Salmonella enterica serovar Typhi on the clinical response to ofloxacin therapy. *PLoS Negl. Trop. Dis.* **5,** e1163 (2011).

14. Humphries, R. M., Fang, F. C., Aarestrup, F. M. & Hindler, J. a. In vitro susceptibility testing of fluoroquinolone activity against salmonella: Recent changes to CLSI standards. *Clin. Infect. Dis.* **55,** 1107–1113 (2012).

15. Arjyal, A. *et al.* Gatifloxacin versus chloramphenicol for uncomplicated enteric fever: an open-label, randomised, controlled trial. *Lancet Infect. Dis.* **11,** 445–54 (2011).

16. Crump, J. A., Sjölund-Karlsson, M., Gordon, M. A. & Parry, C. M. Epidemiology, Clinical Presentation, Laboratory Diagnosis, Antimicrobial Resistance, and Antimicrobial Management of Invasive Salmonella Infections. *Clin. Microbiol. Rev.* **28,** 901–37 (2015).

17. Basnyat, B. The treatment of enteric fever. *J R Soc Med* **100,** 161–162 (2007).

18. Waddington, C. S. *et al.* An Outpatient, Ambulant Design, Controlled Human Infection Model Using Escalating Doses of Salmonella Typhi Challenge Delivered in Sodium Bicarbonate Solution. *Clin. Infect. Dis.* ciu078– (2014). doi:10.1093/cid/ciu078

19. Parry, C. M., Hien, T. T., Dougan, G., White, N. J. & Farrar, J. J. Typhoid fever. *N. Engl. J. Med.* **347,** 1770–1782 (2002).

20. Maskey, A. P. *et al.* Salmonella enterica serovar Paratyphi A and S. enterica serovar Typhi cause indistinguishable clinical syndromes in Kathmandu, Nepal. *Clin. Infect. Dis.* **42,** 1247–53 (2006).

21. Sabbagh, S. C., Forest, C. G., Lepage, C., Leclerc, J. M. & Daigle, F. So similar, yet so different: Uncovering distinctive features in the genomes of Salmonella enterica serovars Typhimurium and Typhi. *FEMS Microbiol Lett* **305,** 1–13 (2010).

22. Parkhill, J. *et al.* Complete genome sequence of a multiple drug resistant Salmonella enterica serovar Typhi CT18. *Nature* **413,** 848–52 (2001).

23. McClelland, M. *et al.* Comparison of genome degradation in Paratyphi A and Typhi, human-restricted serovars of Salmonella enterica that cause typhoid. *Nat. Genet.* **36,** 1268–74 (2004).

24. Spanò, S. & Galán, J. E. A Rab32-dependent pathway contributes to Salmonella typhi host restriction. *Science* **338,** 960–3 (2012).

25. Dougan, G. & Baker, S. Salmonella enterica serovar typhi and the pathogenesis of typhoid fever. *Annual Review of Microbiology* **68,** 317–336 (2014).

26. Rodriguez-Rivera, L. D., Bowen, B. M., den Bakker, H. C., Duhamel, G. E. & Wiedmann, M. Characterization of the cytolethal distending toxin (typhoid toxin) in non-typhoidal Salmonella serovars. *Gut Pathog.* **7,** 19 (2015).

27. Feasey, N. A., Dougan, G., Kingsley, R. A., Heyderman, R. S. & Gordon, M. A. Invasive non-typhoidal salmonella disease: an emerging and neglected tropical disease in Africa. *Lancet* **379,** 2489–2499 (2012).

28. Haghjoo, E. & Galán, J. E. Salmonella typhi encodes a functional cytolethal distending toxin that is delivered into host cells by a bacterial-internalization pathway. *Proc. Natl. Acad. Sci. U. S. A.* **101,** 4614–9 (2004).

29. Song, J., Gao, X. & Galán, J. E. Structure and function of the Salmonella Typhi chimaeric A(2)B(5) typhoid toxin. *Nature* **499,** 350–4 (2013).

30. Spanò, S., Ugalde, J. E. & Galán, J. E. Delivery of a Salmonella Typhi exotoxin from a host intracellular compartment. *Cell Host Microbe* **3,** 30–8 (2008).

31. Deng, L. *et al.* Host Adaptation of a Bacterial Toxin from the Human Pathogen Salmonella Typhi. *Cell* **159,** 1290–1299 (2014).

32. Liang, L. *et al.* Immune profiling with a Salmonella Typhi antigen microarray identifies new diagnostic biomarkers of human typhoid. *Sci Rep* **3,** 1043 (2013).

33. Charles, R. C. *et al.* Immunoproteomic analysis of antibody in lymphocyte supernatant in patients with typhoid fever in Bangladesh. *Clin. Vaccine Immunol.* **21,** 280–5 (2014).

34. Hornick, R. & Greisman, S. Typhoid fever: pathogenesis and immunologic control. 2. *New Engl. …* **283,** 739–46 (1970).

35. Waddington, C. S. *et al.* Advancing the management and control of typhoid fever: a review of the historical role of human challenge studies. *J. Infect.* **68,** 405–18 (2014).

36. Hornick, R. B. *et al.* Typhoid fever: pathogenesis and immunologic control. *N. Engl. J. Med.* **283,** 686–91 (1970).

37. Waddington, C. S. *et al.* An outpatient, ambulant-design, controlled human infection model using escalating doses of Salmonella Typhi challenge delivered in sodium bicarbonate solution. *Clin.*

Infect. Dis. **58,** 1230–40 (2014).

38. Ferreccio, C. *et al.* Efficacy of ciprofloxacin in the treatment of chronic typhoid carriers. *J. Infect. Dis.* **157,** 1235–9 (1988).

39. Black, R. E. *et al.* Case-control study to identify risk factors for paediatric endemic typhoid fever in Santiago, Chile. *Bull. World Health Organ.* **63,** 899–904 (1985).

40. Gasem, M. H., Dolmans, W. M., Keuter, M. M. & Djokomoeljanto, R. R. Poor food hygiene and housing as risk factors for typhoid fever in Semarang, Indonesia. *Trop. Med. Int. Health* **6,** 484–90 (2001).

41. Thomas, H. L., Addiman, S. & Mellanby, A. Evaluation of the effectiveness and efficiency of the public health management of cases of infection due to Salmonella typhi/paratyphi in North East London. *Public Health* **120,** 1188–93 (2006).

42. Braddick, M. R. & Sharp, J. C. Enteric fever in Scotland 1975-1990. *Public Health* **107,** 193–8 (1993).

43. Round, J. L. & Mazmanian, S. K. The gut microbiota shapes intestinal immune responses during health and disease. *Nat. Rev. Immunol.* **9,** 313–23 (2009).

44. Khader, S. A. & Gopal, R. IL-17 in protective immunity to intracellular pathogens. *Virulence* **1,** 423–7

45. McArthur, M. A. & Sztein, M. B. Heterogeneity of multifunctional IL-17A producing S. Typhi-specific CD8+ T cells in volunteers following Ty21a typhoid immunization. *PLoS One* **7,** (2012).

46. Lee, S.-J. *et al.* Temporal expression of bacterial proteins instructs host CD4 T cell expansion and Th17 development. *PLoS Pathog.* **8,** e1002499 (2012).

47. Raffatellu, M. *et al.* Simian immunodeficiency virus-induced mucosal interleukin-17 deficiency promotes Salmonella dissemination from the gut. *Nat. Med.* **14,** 421–8 (2008).

48. Sollid, J. U. E., Furberg, A. S., Hanssen, A. M. & Johannessen, M. Staphylococcus aureus: determinants of human carriage. *Infect. Genet. Evol.* **21,** 531–41 (2014).

49. Sztein, M. B. Cell-mediated immunity and antibody responses elicited by attenuated Salmonella enterica Serovar Typhi strains used as live oral vaccines in humans. *Clin. Infect. Dis.* **45 Suppl 1,** S15–9 (2007).

19. APPENDIX A: Grading the severity of solicited and unsolicited systemic Adverse Events

Adverse event	Grade	Definition (in degrees Celsius)
Temperature	0	< 37.6
	1	37.6 – 38.0
	2	38.1 – 39.0
	3	> 39

Adverse event	Grade	Definition
Any symptom	0	Absence or resolution of symptom
	1	Awareness of symptom but tolerated; transient or mild discomfort; little or no medical intervention required
	2	Discomfort enough to cause limitation of usual activity; some medical intervention or therapy required
	3	Significant interference with daily activity
	4	Emergency department visit or hospitalisation

20. APPENDIX B: Grading the severity of visit observed Adverse Events

Observation	Grade 1	Grade 2	Grade 3
Oral temperature (°C)	37.6 – 38.0	38.1 – 39.0	Greater or >39.1
Tachycardia (beats/min)	101-115	116-130	>130
Bradycardia (beats/min)	50-54	45-49	<45
Systolic hyper-tension (mmHg)	141-150	151-155	>155
Diastolic hyper-tension (mmHg)	91-95	96-100	>100
Systolic hypo-tension (mmHg)	85-89	80-84	<80

The following ranges are considered normal physiological ranges and are recorded as Grade 0:

- Oral temperature between 35.5 and 37.5 C
- Resting heart rate between 55 and 100 beats/minute
- Systolic blood pressure between 90 and 140 mmHg

21. APPENDIX C: Grading the severity of laboratory Adverse Events

Parameter	Grade 1	Grade 2	Grade 3	Grade 4*
**Haemoglobin: decrease from baseline value (gm/dl)**	< 1.5	1.5-2.0	2.1-5.0	>5
**White cell count: elevated (cell/mm^3)**	10,800–15,000	15,001–20,000	20,001–25,000	>25,000
**White cell count: depressed (cells/mm3)**	2500-3500	1500-2499	1000-1499	<1000
**Neutrophil count (cells/mm^3)**	1500-2000	1000-1499	500-999	<500
**Platelets (cells/mm^3)**	125,000-140,000	100,000-124,000	25,000-99,000	<25,000
**Sodium: hyponatraemia (mmol/L)**	132–134	130–131	125–129	<125
**Sodium: hypernatraemia (mmol/L)**	144–145	146–147	148–150	>150
**Potassium: hyperkalaemia (mmol/L)**	5.1–5.2	5.3–5.4	5.5–5.6	>5.6
**Potassium: hypokalaemia (mmol/L)**	3.5–3.6	3.3–3.4	3.1–3.2	<3.1
**Urea (mmol/L)**	8.2–8.9	9.0–11	>11	RRT
**Creatinine (µmol/L)**	132-150	151-176	177-221	>221 or RRT
**ALT and/or AST (IU/L)**	1.1–2.5 x ULN	>2.6–5.0 x ULN	5.1-10 x ULN	>10 x ULN
**Bilirubin, with increase in LFTs (µmol/L)**	1.1–1.25 x ULN	1.26–1.5 x ULN	1.51–1.75 x ULN	>1.75 x ULN
**Bilirubin, with normal LFTs (µmol/L)**	1.1–1.5 x ULN	1.6–2.0 x ULN	2.1–3.0 x ULN	>3.0 x ULN
**Alkaline phosphatase (IU/L)**	1.1–2.0 x ULN	2.1–3.0 x ULN	3.1–10 x ULN	>10 x ULN
**Amylase (IU/L)**	1.1–1.5 x ULN	1.6–2.0 x ULN	2.1–5.0 x ULN	>5.0 x ULN
**Albumin: hypoalbuminaemia (g/L)**	28–31	25–27	<25	Not applicable
**C-reactive protein**	>10-30	31-100	101-200	>200

Grade 4 Potentially life threatening*

22. APPENDIX D: AMENDMENT HISTORY

Amendment No.	Protocol Version No.	Date issued	Author(s) of changes	Details of Changes made
**1.0**	**2.0**	**28-Nov-2016**	**M Gibani, Claire Jones**	**Table 1 – Removal of D0+6 visit**<br><br>**Table 2 – Removal of D0+6h visit. Amendment to sample collection schedule.**<br><br>**Section 7.3 – Exclusion Criteria**<br>Amendments to exclusion criteria<br>Prior vaccination with an oral typhoid vaccines (e.g. Ty21a) or other investigational typhoid vaccine (e.g. Vi-conjugate vaccine, MO1ZH09)<br>Prior vaccination with a Vi-polysaccharide typhoid vaccine administered within 5 years from the time of screening.<br>Prior vaccination with a Vi-polysaccharide typhoid vaccine administered more than 5 years from the time of screening AND detectable Vi-antibody titre at screening (defined as IgG ≥7.4U/ml measured using the VaccZyme *Salmonella* Typhi Vi IgG kit, Binding Site ® UK).<br><br>**Section 8.3 - Antibiotic treatment**<br>Change of first line antibiotic from azithromycin to ciprofloxacin.<br><br>**Section 9.1 – *S.* Typhi challenge strain**<br>Correction to details on whole genome sequencing, performed at the Sanger Institute, UK<br><br>**Section 12.5 Definitions of secondary clinical and microbiological end-points** – Additional details on definitions of secondary clinical and microbiological endpoints<br><br>**Section 15.6 Participant reimbursement** – Correction to reimbursement amounts
**2.0**	**2.1**	**09-Feb-2017**	**M Gibani**	**Corrected reimbursement amounts**
**2.1**	**2.2**	**21-Mar-2017**	**C Boyer**	**Corrected version number and date**<br>**Added CT.gov number**

**3.0**	**3.0**	**17-Jul-2017**	**M Gibani**	Objectives: Error corrected, urine sample collected missed from exploratory objectives.<br><br>Change in molecular diagnostic tube at Typhoid Diagnosis (TD) to Streck tube (Table 2).<br><br>Section 10.3 - Additional information on molecular diagnostic testing.<br><br>Minor typographical corrections.
**4.0**	**3.0**	**19-Jan-2018**	**M Gibani, C Boyer, D Campbell**	**Section 2 & 5**<br>Addition of new exploratory objective 'To investigate recruitment methods and reasons for volunteer exclusions from typhoid challenge models'. Amendment to existing exploratory objective, to include urine samples for development of novel diagnostics.<br><br>**Section 4.5**<br>Rationale for new exploratory objective.<br>**Section 6**<br>Addition of an * to stool samples in Table 2 Sample Collection Plan which was missed in error.<br><br>**Section 12.2**<br>Analysis of new exploratory objective.
**5.0**	**4.0**	**16-Feb-2018**	**C Boyer, D Campbell**	Merging protocol v3.0 (17-Jul-2017) and protocol v3.0 (19-Jan-2018) due to errors in substantial amendment 4.0, whereby protocol version v2.2 (21-Mar-2017) was updated instead of v3.0 (17-Jul-2017)<br><br>No new information has been introduced.
